# Engineered endolysin-based “artilysins” for controlling the gram-negative pathogen *Helicobacter pylori*

**DOI:** 10.1186/s13568-021-01222-8

**Published:** 2021-04-28

**Authors:** Dengyuan Xu, Shanshan Zhao, Jun Dou, Xiaofeng Xu, Yanyan Zhi, Liangzhu Wen

**Affiliations:** 1 China Pharmaceutical University, Nanjing, 211100 China; 2 Wanbang Pharmatech Co., Ltd, Xuzhou, 221004 China

**Keywords:** *Escherichia coli* expression system, *Helicobacter pylori*, Holin and endolysin phage, In vitro antibacterial assays

## Abstract

*Helicobacter pylori* infection can cause a variety of gastrointestinal diseases. In severe cases, there is a risk of gastric cancer. Antibiotics are often used for clinical treatment of *H. pylori* infections. However, because of antibiotic overuse in recent years and the emergence of multidrug-resistant bacteria, there is an urgent need to develop new treatment methods and drugs to achieve complete eradication of *H. pylori*. Endolysins and holins encoded by bacterial viruses (i.e., phages) represent a promising avenue of investigation. These lyase-based antibacterial drugs act on the bacterial cell wall to destroy the bacteria. Currently, a type of endolysin that has been studied more frequently acts on the amide bond between peptidoglycans, and holin is a transmembrane protein that can punch holes in the cell membrane. However, as a Gram-negative bacterium, *H. pylori* possesses a layer of impermeable lipopolysaccharides on the cell wall, which prevents endolysin interaction with the cell wall. Therefore, we designed a genetic linkage between an endolysin enzyme and a holin enzyme with a section of polypeptides (e.g., polycations and hydrophobic peptides) that enable penetration of the outer membrane. These complexes were designated “artilysins” and were efficiently expressed in *Escherichia coli*. In vitro bacteriostasis experiments showed that the purified artilysins had strong bacteriostatic effects on *H. pylori*. In addition, the surface of *H. pylori* was perforated and destroyed, as confirmed by electron microscopy, which was proved that artilysins had bacteriolytic effect on *H. pylori*.

**Supplementary Information:**

The online version contains supplementary material available at 10.1186/s13568-021-01222-8.

## Key points

- *“*Artilysins” had a strong lytic effect on *Helicobacter pylori*.
- Artilysins had more specific effects than those of broad-spectrum antibiotics.
- The artilysin design could be used to treat most Gram-negative bacterial infections.

## Introduction

*Helicobacter pylori* (*H. pylori*) is a Gram-negative bacterium with a high infection rate and a strong association with stomach diseases (95% of stomach diseases, including stomach cancer). Children under 5 years of age represent a key population for transmission of *H. pylori*. However, the infection rate is positively correlated with age and is a cumulative process. Among individuals aged ≥ 19 years, the infection rate can reach 75% (Isaeva 2019; Lyu et al. 2020). Currently, antibiotics are used for clinical treatment of *H. pylori* infection, and the most commonly used treatments are standard triple and quadruple therapies. However, because of antibiotic overuse, *H. pylori* resistance to antibiotics is becoming an increasingly serious problem (You et al. 2016). Despite extensive research, effective new antibiotics are rare, and antibiotic treatments cannot completely cure *H. pylori* infections. Therefore, a new method is needed to develop antibacterial agents that can control *H. pylori* infection without drug resistance. Some studies have demonstrated successful use of phage endolysins as a treatment for specific host infections (e.g., *Mycobacterium tuberculosis*, *Staphylococcus aureus*, and *Escherichia coli*) (O'Flaherty et al. 2009; Harhala et al. 2018; Fraga et al. 2019; Kovalskaya et al. 2019; Yu et al. 2019; de Miguel et al. 2020; Kim et al. 2020). Endolysins act rapidly on bacteria, limiting their ability to develop resistance (Gondil et al. 2020). Moreover, these enzymes are easy to obtain, design, and modify (Borysowski et al. 2006; Fischetti 2008; Gondil et al. 2020). They can be improved via genetic engineering, and their ease of expression and purification is suitable for industrial production (Sao-Jose 2018).

Because *H. pylori* is a Gram-negative bacterium, it possesses an outer membrane outside the cell wall, which prevents endolysin interaction with the cell wall (Ghose and Euler 2020). Here, we designed a transmembrane peptide and connected it to a two-component cleavage system (endolysin–holin) using GAGA repeat sequences as a linker to construct artilysins (Yan et al. 2019). An *E. coli* expression system was used to express the target genes, and the protein products were purified by 3 K ultrafiltration and chromatography. Our analyses demonstrated that the purified product possessed strong antibacterial activity in vitro.

## Materials and methods

### Bacterial strains, media, and growth conditions

This study used *H. pylori* strain ATCC 700392 as a reference strain for in vitro bacteriostasis experiments. The *H. pylori* were cultured in solid and liquid media under microaerobic conditions at 37℃ for about 48–72 h. The solid medium was purchased from Qingdao Haibo Biotechnology Co., Ltd., which contained 4 g/l bovine brain extract powder, 4 g/l bovine heart extract powder, 5 g/l peptone, 16 g/l casein peptone, 2 g/l glucose, 5 g/l sodium chloride, 2.5 g/l disodium hydrogen phosphate, and 13.5 g/l agar. The liquid culture medium was also purchased from Qingdao Haibo Biotechnology Co., Ltd., which contained 10 g/l bovine brain extract powder, 9 g/l bovine heart extract powder, 10 g/l peptone, 2 g/l glucose, 5 g/l sodium chloride, and 2.5 g/l disodium hydrogen phosphate. After high-pressure steam sterilization at 121 °C for 15 min of the above two media, 7% sterile defibrinated sheep blood and *H. pylori* bacteriostatic agent (containing 1 mg nalidixic acid, 0.5 mg trimethoprim, 0.3 mg vancomycin, and 0.2 mg amphotericin B) were added. The above-mentioned sterile defibrinated sheep blood and *H. pylori* bacteriostatic agent were provided by the manufacturer when the mediums were purchased. *E. coli* strain BL21(DE3) was cultured in LB medium (15 g/l yeast extract powder, 1 g/l glucose, and 10 g/l sodium chloride) at 37 ℃. When necessary, an additional 40 µg/ml kanamycin was added to LB medium.

### Design of artilysin genes

Bioinformatics prediction and analysis were performed using the published complete genome sequence of *H. pylori* phage KHP30 (NC_019928.1) and 1961P (NC_019512.1) (Lehours et al. 2011; Luo et al. 2012; Uchiyama et al. 2012, 2013; Abdel-Haliem and Askora 2013; Takeuchi et al. 2018). Preliminary annotations and reports of *H. pylori* phage endolysin and holin were used in combination with the NCBI BLAST program (e-values ≤ 0.01 were considered credible); the smallest item in the credible range was subjected to the next round of BLAST. This was repeated until no obvious homologues appeared, and possible enzymatic functions were identified using the annotated *H. pylori* phage endolysin. The (ExPASy) Protparam software was used to analyze basic biochemical properties (e.g., amino acid length, molecular weight, isoelectric point, charge number, and hydrophilicity). Signal P3.0 software was used to analyze N-terminal amino acid sequences to determine whether they contained signal peptides. The SWISS-MODEL automatic matching method was used to predict and compare tertiary structures, using all default parameters. The above analysis and comparison were expected to identify multiple *H. pylori* bacteriophage endolysin and holin sequences, including Holin A, Holin B, Endolysin A and Endolysin B (see Additional file 1: Table S1). In addition, during the early stages of this study, extensive literature review and data analysis revealed several transmembrane peptides with different physical and chemical properties. Their sequences or optimized sequences (see Additional file 1: Table S2) were connected to *H. pylori* phage endolysin and holin using a linker (GAGA), thereby producing artilysin genes (see Additional file 1: File S1).

### Plasmid construction

Early experiments showed that endolysins formed inclusion bodies during prokaryotic expression in vitro, which reduced their cleavage activity. Therefore, the pSUMO soluble *E. coli* expression vector purchased from Miao Ling Biological Technology Co., Ltd. was used, which had His-Tag and could follow by nickel column affinity chromatography to capture the target protein. An enzyme digestion–enzyme ligation method was used to construct recombinant pSUMO-artilysin plasmids. For the artilysin genes and pSUMO, *Xho* I and *Sac* I were used for digestion. The optimized target genes did not contain *Xho* I or *Sac* I linearization sites.

### Construction and expression of engineered bacteria

For the *E. coli* expression system, competent *E. coli* BL21 cells were prepared using the calcium chloride method and then heat shocked for plasmid transformation. Kanamycin-containing agar was used to obtain clones expressing the plasmid. Sequences were verified to confirm the construction of BL21-pSUMO-artilysin bacteria.

Protein expression conditions (e.g., induction temperature, induction timing, induction time, inducing agent, medium composition, and nutrients) were adjusted as necessary to optimize expression in the engineered bacteria. Two recombinant engineered bacterias (BL21-pSUMO-artilysin) were cultured in LB liquid medium on a shaker at 37 ℃ and 250 rpm until the OD_600nm_ was between 0.6 and 1. Then add IPTG with a final concentration of 0.5 mM, and incubate on a shaker at 25 ℃ and 250 rpm for 16–20 h. SDS-PAGE was used to verify artilysin expression.

### Purification

In the *E. coli* expression system, the induced bacteria were centrifuged, and the supernatant were discarded. Bacterial cells were then crushed using a high pressure homogenizer at the condition of 850 bar. After further centrifugation at the condition of 8500 rpm and 30 min, the precipitate were discarded, and the supernatant were separated by nickel-chelating affinity chromatography to obtain purer target proteins (Ding et al. 2020). The Tris–HCl system was selected as the mobile phase, and 200 mM imidazole was added to the eluent, and the elution peak was collected by gradient elution. Because proteins expressed using the pSUMO vector was fused to the SUMO protein, they were digested with the SUMO enzyme (500 U/g) purchased from Solarbi Co., Ltd. to obtain the target proteins. The molecular weight and the pI of artilysin 1 were 30.69 kDa and 9.69, while the molecular weight and the pI of artilysin 2 were 22.02 kDa and 9.01. According to the protein content of the elution peak, add SUMO enzyme according to the enzyme activity of 500 u/g, and digest the collected elution peak for 16 h. SDS-PAGE was used for analysis. After the above purification, preliminary artilysins were obtained. A 3K hollow fiber column was used to concentrate the artilysins to 10^3^ μg/ml and replace the artilysins with 20 mM phosphate buffer.

### Antibacterial assays

The Kirby–Bauer test was used as an in vitro antibacterial assay. *H. pylori* was spread on solid medium, and sterile filter paper was attached to the plates after inoculation of the *H. pylori*. Subsequently, 20–30 µl artilysins with a concentration of 10^3^ μg/ml were added to the filter paper. Antibiotics and lysozyme were used as positive controls, while 20 mM phosphate buffer and the supernatant of BL21 broken bacteria with 500 u/g SUMO enzyme were used as negative controls. The antibiotic mixture was composed of gentamicin, levofloxacin and amoxicillin, and three antibiotics were formulated into a mixed solution with a final concentration of 10^3^ μg/ml in equal proportions. All three antibiotics were purchased from Shanghai Macleans Biochemical Technology Co., Ltd. Lysozyme was purchased from Solebold Technology Co., Ltd., and was prepared at 10^3^ μg/ml with purified water. Bacteria were incubated at 37℃ under microaerobic conditions for 48 h, and inhibition zones were examined at 48 h. The size of the inhibition zone was compared between artilysin and positive controls.

In addition, artilysins were added directly to *H. pylori* following centrifugation. After the bacteria had been incubated for 2 h on a shaker, they were observed under an electron microscope at 20 min, 1 h, and 2 h. The bacteria were collected by centrifugation, fixed with 2.5% glutaraldehyde and 1% osmium acid. After rinsing with phosphate buffer, dehydrating with ethanol, drying the sample with LEICA CPD-300 automatic critical point dryer, and then using ion sputtering to make the sample conductive with LEICA ACE-600 high vacuum coating machine, and finally can be observed by HITACHI SU8200 scanning electron microscope. The liquid method was also used to verify whether the bacterial concentration decreased after the addition of artilysins. Lysozyme was used as a positive control, and artilysin buffer was used as a negative control. Artilysins were added to robust *H. pylori* cultures at a final concentration of 100 or 500 μg/ml. The bacteria were cultivated for 30 h at 37 ℃ under microaerobic conditions with shaking at 150 rpm. At 0, 6, 24, and 30 h, samples of the bacteria were collected to measure the absorbance at 600 nm (Aiba et al. 2019; Knaack et al. 2019).

## Results

### Artilysins: proof of design

**Fig. 1:**
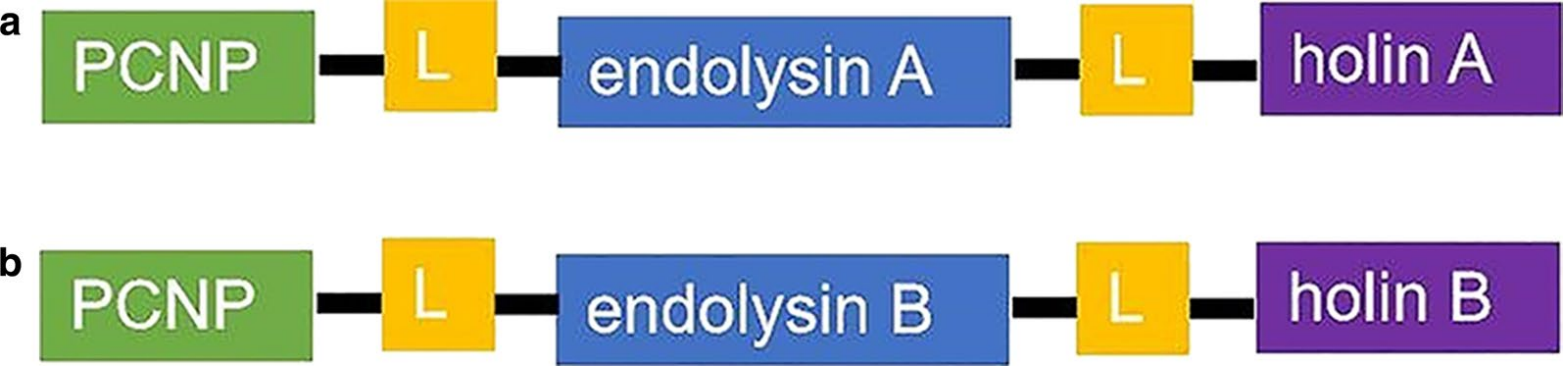
Visual representation of engineered artilysins. Peptides with LPS-destabilizing activity were selected for modification with endolysins and holins identified in this study. Abbreviations: PCNP ,  polycationic nonapeptide; L,  linker consisting of GAGA sequence. **a** The artilysin designed for use in the *E. coli* expression system BL21-pSUMO-artilysin1 is shown. **b** The artilysin designed for use in the *E. coli* expression system BL21-pSUMO-artilysin2 is shown

The critical aspect of the artilysin design involves modification of endolysin and holin with a lipopolysaccharide (LPS)-destabilizing peptide. The LPS layer was stabilized by ionic interactions between the dications and phosphate groups, as well as hydrophobic accumulation within the lipid A portion. Considering the physical and chemical properties (e.g., cationic, hydrophobic, or amphiphilic) that interfered with the stabilizing force, a peptide with proven LPS-destabilizing activity was selected. Bioinformatics analysis revealed two possible endolysin genes (endolysins A and B) and two possible holin genes (holins A and B) from the *H. pylori* bacteriophage genome. These were combined with LPS-destabilizing peptides to form two artilysins (Fig. 1; see also Additional file 1: Table S1).

**Fig. 2:**
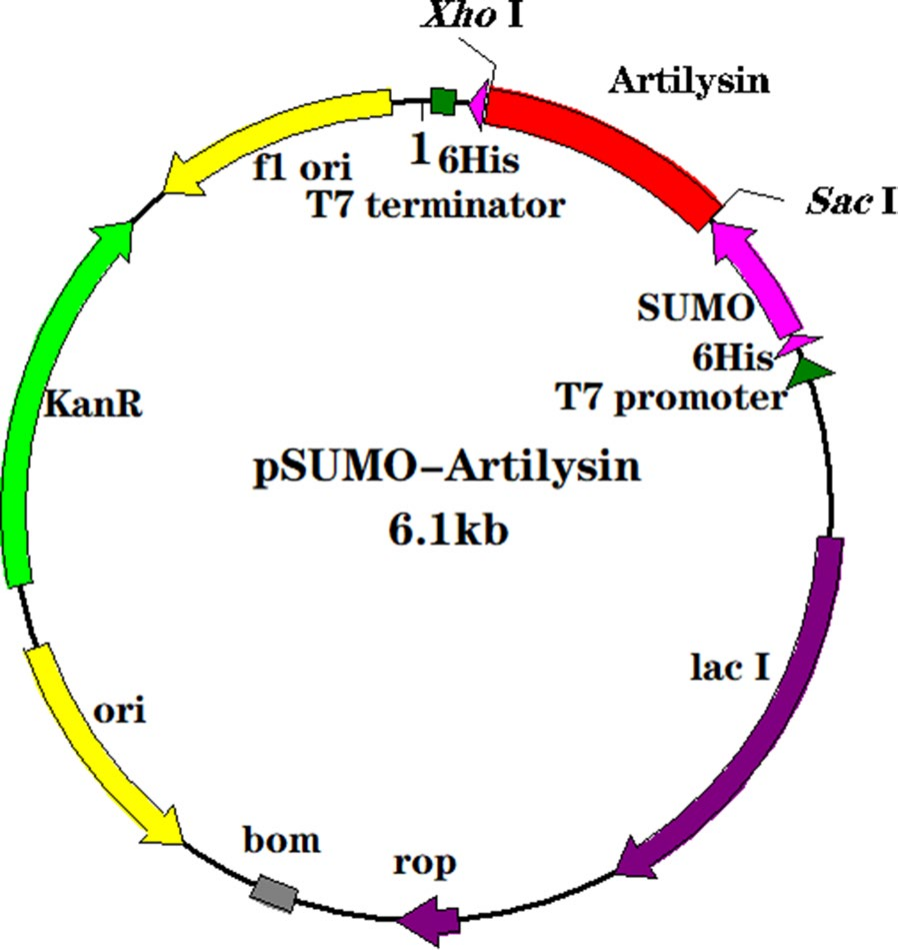
Recombinant plasmid map. pSUMO-artilysin is a recombinant plasmid designed for expression in *E. coli*. The red element represents artilysin 1 or 2. The restriction sites are *Xho* I and *Sac* I

After a series of enzyme digestion and enzyme linking processes, the designed artilysin gene sequences were successfully ligated to the plasmid vector to obtain the recombinant plasmids pSUMO-artilysin1 and pSUMO-artilysin2 (Fig. 2). These plasmids were transformed into host bacteria to obtain the engineered bacteria BL21-pSUMO-artilysin1 and BL21-pSUMO-artilysin2.

### Expression and purification of artilysins

**Fig. 3:**
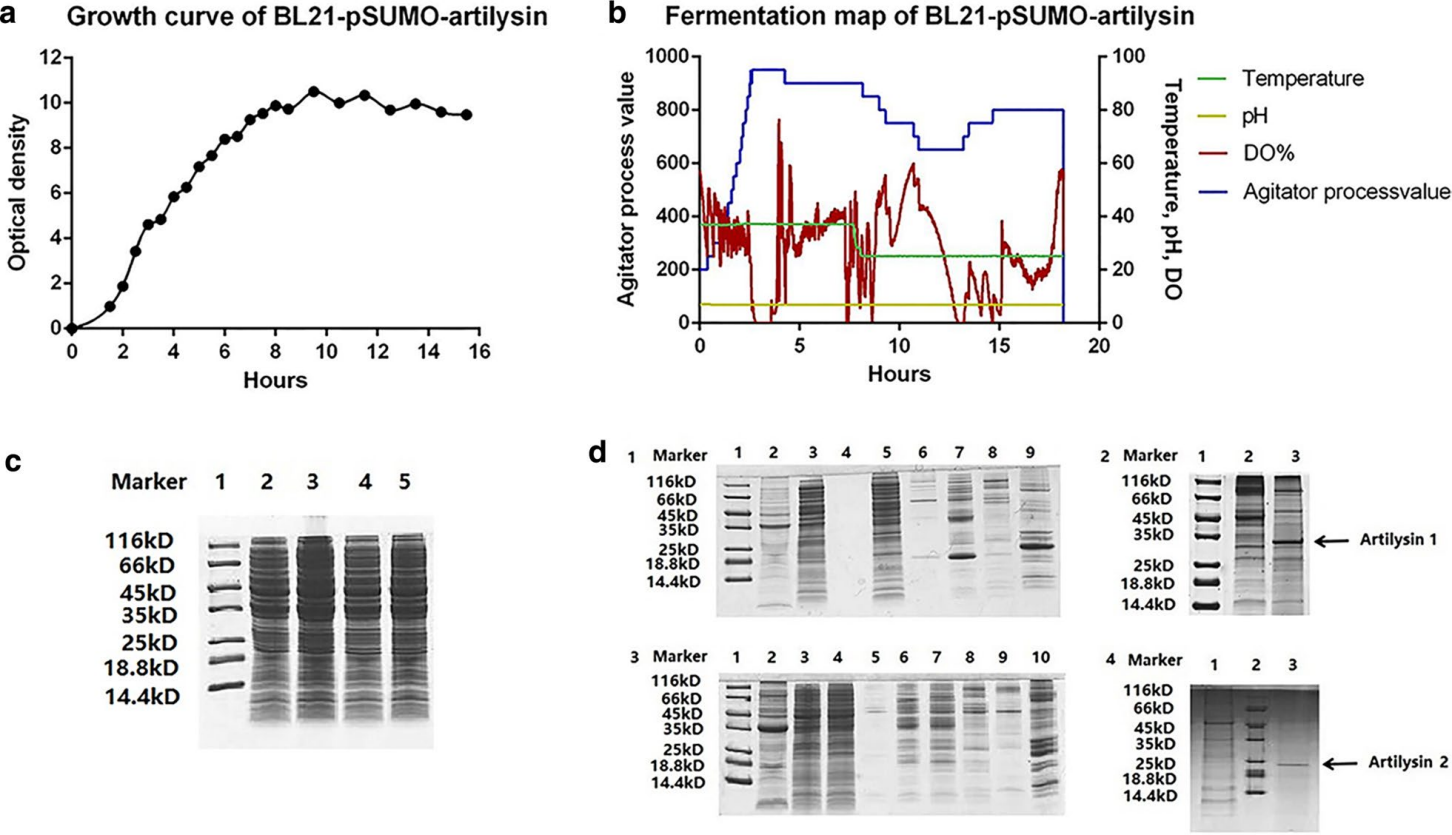
*E. coli* expression system using BL21-pSUMO-artilysin engineered bacteria for protein production and purification. **a** The growth curve for BL21-pSUMO-artilysin at 37 ℃. **b** Fermentation map following high-density fermentation in a 5-l fermenter. **c** SDS-PAGE gel of BL21-pSUMO-artilysin before and after the addition of isopropyl β-d-1-thiogalactopyranoside. 1: Marker; 2: BL21-pSUMO-artilysin1 bacteria before induction; 3: BL21-pSUMO-artilysin1 bacteria after induction; 4: BL21-pSUMO-artilysin2 bacteria before induction; 5: BL21-pSUMO-artilysin2 bacteria after induction. **d** SDS-PAGE gels (1 and 3) of the collected eluates. The later peak contains fewer impurities, and the target protein purity is greater. (1) 1: Marker; 2: Precipitate after lysis of BL21-pSUMO-artilysin1 bacteria; 3: Supernatant after lysis of BL21-pSUMO-artilysin1 bacteria; 4: Blank; 5: Flowthrough; 6: Postpeak of elution; 7: Middle peak of elution; 8: Prepeak of elution; 9: Alkaline wash. (3) 1: Marker; 2: Precipitate after lysis of BL21-pSUMO-artilysin2 bacteria; 3: Supernatant after lysis of BL21-pSUMO-artilysin2 bacteria; 4: Flowthrough; 5: Prepeak of elution; 6: Middle peak of elution-1; 7: Middle peak of elution-2; 8: Middle peak of elution-3; 9: Postpeak of elution; 10: Alkaline wash. SUMO enzyme was used to digest the EP-LP, thus producing artilysins. SDS-PAGE protein gels (2 and 4) showing artilysins after preliminary purification. (2) 1: Marker; 2: SUMO-artilysin1; 3: artilysin 1. (4) 1: Marker; 2: SUMO-artilysin2; 3: artilysin 2

For the *E. coli* expression system, the growth curve of the engineered bacteria BL21-pSUMO-artilysin was divided into four phases: lag, logarithmic growth, stationary, and decline (Fig. 3a). High-density fermentation conditions were optimized as follows: the bacteria were cultured at 37 °C for 3 h in a 5-l fermenter and then induced by the addition of isopropyl β-d-1-thiogalactopyranoside with final concentration of 0.5 mM to stimulate protein expression at 25 °C for 10 h. The fermentation profile involved modification of various parameters, such as temperature, pH, stirring speed, and dissolved oxygen (Fig. 3b; see also Additional file 1: Table S3). After induction, the cell concentration continued to increase slowly, but there was no obvious change in the position and density of the protein bands. SDS-PAGE analysis of proteins from whole bacteria did not demonstrate obvious differences and we could not effectively judge whether the target protein was expressed (Fig. 3c). After fermentation, the total bacterial weights were 509 and 596 g, with unit bacterial weights of 149.7 and 175.3 g/l. After preliminary purification using nickel column affinity chromatography, SUMO-artilysin proteins were obtained. Competitive elution was performed using imidazole. When the imidazole concentration was approximately 25 mM, the target protein was replaced by imidazole and eluted. The elution peaks were collected, protein concentrations were measured, and SDS-PAGE was performed to detect the proteins. Subsequently, the SUMO-artilysin proteins were activated by the SUMO enzyme, and electrophoresis was repeated. SDS-PAGE showed that the SUMO-artilysin proteins were cleaved by the SUMO enzyme, and the resulting artilysins were consistent with our expectation (Fig. 3d).

### Artilysins showed in vitro antibacterial efficacy

**Fig. 4:**
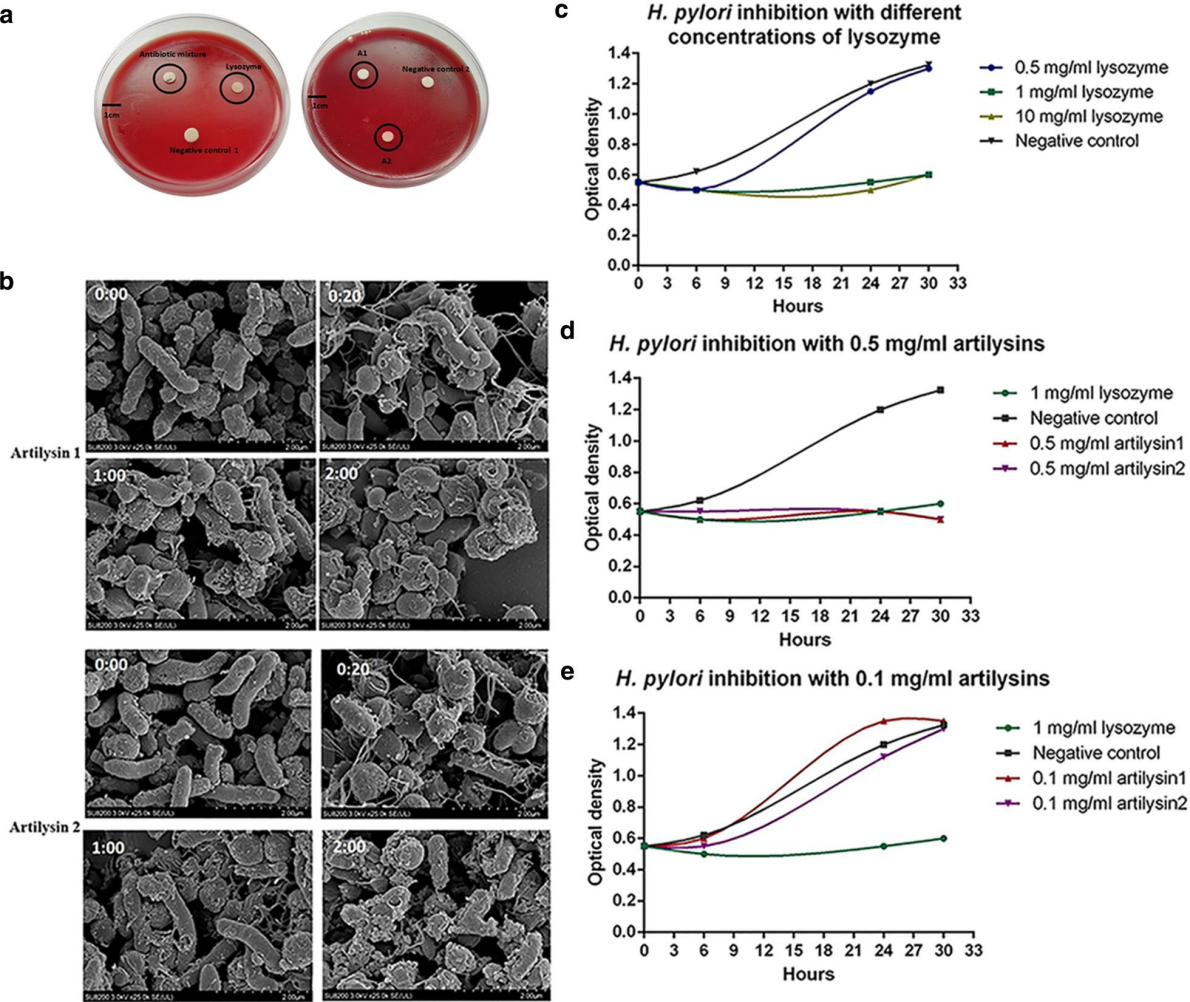
In vitro antibacterial activities of artilysins. **a** Inhibition zone measured by the filter paper method. The petri dish diameter is 9 mm. The spots within the black circle are the corresponding inhibition zones. The antibiotic mixture and lysozyme were specifically explained in the article. A1 was Artilysin 1, A2 was Artilysin 2, Negetive control 1 was 20 mM phosphate buffer, and Negetive control 2 was the supernatant of BL21 broken bacteria with 500 u/g SUMO enzyme. **b** Electron microscopy images of artificial enzyme activity on *H. pylori* over 2 h. Time points are expressed as h:min (scale bar, 2 µm). **c** In vitro antibacterial effects of 500, 10^3^, and 10^4^ μg/ml lysozyme on *H. pylori*. **d** In vitro antibacterial effects of 500 μg/ml artilysins on *H. pylori*. **e** In vitro antibacterial effects of 100 μg/ml artilysins on *H. pylori*

The Kirby–Bauer test was used to detect the antibacterial activity of artilysins in vitro, and the presence of the inhibition zone could be detected by the eyes alone. However, because artilysins were only crudely purified, and their concentrations were low, the effect was less robust than that of antibiotics. In addition, because *H. pylori* was transparent and did not grow in a single colony, it was not easily observed when grown on blood agar. Therefore, although we could visually observe the presence of a small, smooth, transparent inhibition zone around each filter paper, this zone was nearly invisible in photos (Fig. 4a). The inhibition zone diameters induced by antibiotics and lysozyme were approximately 1 and 1.5 cm, respectively, and those induced by artilysins 1 and 2 were approximately 0.75 and 0.78 cm, respectively. In the experiment, we dropped 20 µl of concentrations of 10^3^ μg/ml of artilysins on blank filter sheet, which indicated that only 20 µg of artilysins could play a significant antibacterial effect. Both negative controls had no inhibition zone, which indicate that there was no antibacterial effect in phosphate buffer of artilysin and SUMO enzyme.

Subsequently, we observed the microstructure of the bacteria using an electron microscope and found that the peptidoglycan structures of the cell wall and membrane were also degraded. Moreover, the cell surface was perforated, and the bacteria were eventually degraded (Fig. 4b). *H. pylori* treated with artilysins 1 and 2 gradually transitioned from a spiral to spherical shape, and the pores in the cell wall and membrane gradually increased, resulting in lysis. These observations are consistent with the artilysin mechanism of action we guess. First, the LPS layer is destroyed by polycationic nonapeptides and hydrophobic polypeptides; then, the peptidoglycan structure of the cell wall is degraded by endolysins, and the cell membrane is perforated by holins, which causes bacterial destruction. In order to confirm the above mechanism, we also needed a large number of tests to supplement.

In this experiment, we used the liquid method as a replacement for the Kirby–Bauer test to detect the antibacterial activity of artilysins in vitro. First, we used lysozyme to establish an evaluation method. A sterilized cotton swab was used to scrape established cultures of *H. pylori* from blood agar into the liquid medium, achieving an absorbance at 600 nm of approximately 1. After the addition of a stock lysozyme solution, the absorbance of the bacteria was 0.6 at 600 nm. The final lysozyme concentrations used were 500, 10^3^, and 10^4^ μg/ml. When cultured for 6 h, compared with the negative control, the bacteria treated with lysozyme showed growth inhibition (Fig. 4c). The 500 μg/ml lysozyme concentration caused minimal change in growth after 6 h of culture, whereas 10^3^ and 10^4^ μg/ml concentrations showed obvious antibacterial effects until 30 h of culture. The bacterial growth increased with increasing culture time. The rate of bacterial growth of cultures treated with 500 μg/ml lysozyme was similar to that in the negative control cultures, whereas those of cultures treated with 10^3^ and 10^4^ μg/ml lysozyme were significantly lower, indicating bacterial growth inhibition. Therefore, 500 μg/ml lysozyme had no antibacterial effect on *H. pylori* in vitro, but 10^3^ and 10^4^ μg/ml lysozyme had substantial antibacterial effects.

Next, the liquid method was used to detect the antibacterial activities of artilysins at 6, 24, and 30 h. Considering the growth cycle of *H. pylori*, we chose 6 h as the first sampling point (Aiba et al. 2019). The activity of antibiotics could be maintained for about 48 h under the culture condition, so the last sampling point was selected at 30 h. Artilysins 1 and 2 at a final concentration of 500 μg/ml induced antibacterial activity similar to that induced by lysozyme within 30 h of culture (Fig. 4d). Compared with the negative control of the supernatant of BL21 broken bacteria with 500 u/g SUMO enzyme, the *H. pylori* growth rate was reduced after the addition of artilysins 1 and 2. These antibacterial effects were consistent with those of lysozyme. Artilysins at a final concentration of 100 μg/ml induced no obvious antibacterial activity (Fig. 4e). The minimum antibacterial concentration (MIC) of artilysins were between 100 μg/ml to 500 μg/ml.

## Discussion

This investigation showed that artilysins effectively inhibit *H. pylori*. Kirby–Bauer test indicated that only 20 μg of artilysins could play a significant antibacterial effect. In addition, only added artilysins for 20 min, the surface of the *H. pylori* could observe the obvious perforation, and the effect was quite rapid. The Kirby–Bauer test and liquid method reflected the bacteriostatic effect of the artilysins, while the electron microscopy observes the surface perforation of *H. pylori* to reflect the bacteriolytic effect of the artilysins. The lysis of host bacteria by endolysins is either internal or external. The internal lysis process occurs after the phage has infected the bacteria under natural conditions. Usually, the holin–endolysin system has a synergistic effect. First, holin binds to the cell membrane and destroys its integrity; second, the endolysin passes through the cell membrane pores with holin and reaches the peptidoglycan target, where it degrades the cell wall and eventually causes bacterial lysis. In the external lysis pathway, lysins cause bacterial damage from the external environment. The external lysis route involves mainly Gram-positive bacteria because their peptidoglycan layer is directly exposed to the external environment, without an outer membrane. This enables direct contact and peptidoglycan modification by endolysins. Research on endolysins that target Gram-positive bacteria has been satisfactory, while research on endolysins that target Gram-negative bacteria is developing slightly slow because of interference by the outer membrane (Lai et al. 2020). However, with the development of recent years, the passage of endolysins targeting Gram-negative bacteria has been facilitated in the following ways: (1) identification of natural endolysins that can penetrate the outer membrane, (2) utilization of synergy between endolysins and outer membrane-penetrating agents (e.g., EDTA, citric acid, malic acid, and cationic peptides), and (3) expression of fusion proteins containing endolysins and peptides that can penetrate the outer membrane (Heselpoth et al. 2019; Blasco et al. 2020; Gutierrez and Briers 2020). Here, we constructed fusion proteins containing holin–endolysin and peptides (i.e., artilysins) to penetrate the outer membrane.

Notably, we used a prokaryotic expression system to express artilysins, involving transformation of pSUMO into *E. coli*. Previous studies have shown that SUMO can be used as a fusion tag and molecular chaperone for recombinant protein expression. Use of SUMO can increase the expression of fusion proteins, prevent protease hydrolysis, promote correct target protein folding, and improve solubility of the recombinant protein. In addition, SUMO proteolytic enzymes can efficiently cleave SUMO from the fusion protein to obtain artilysins, the target proteins of this assay (Li et al. 2011; Zhang et al. 2016; Sang et al. 2019). Here, we selected a polycationic nonapeptide to achieve the effect of outer membrane penetration. In future studies, we will design more membrane-permeable peptides and select artilysins with a better cleavage effect after fusion expression (Antonova et al. 2020).

In recent years, multidrug-resistant Gram-negative pathogens have demonstrated inherent or acquired resistance to nearly all available drugs suitable for clinical treatment (Ghosh et al. 2019; Villa and Sieiro 2020). The standard treatment for *H. pylori* is triple or quadruple antibiotic therapy, which is broad-spectrum and rapidly leads to resistance. Our in vitro study showed that both artilysin 1 and 2 exerted antibacterial effects similar to those of antibiotics and lysozyme. Electron microscopy revealed that the cell walls of *H. pylori* were destroyed and the cell membranes perforated, which led to bacterial lysis. These results confirm the ability of artilysins to target and destroy *H. pylori*, thus expanding the strategies available for the treatment of infections with *H. pylori* and other Gram-negative bacteria. In the future, we need more research to further verify the bacteriolytic effect of artilysins on *H. pylori.* For example, we can select probiotics in the gastrointestinal tract, such as *Bifidobacterium* and *Lactobacillus*, to verify the specificity of artilysins. It may prove whether it will destroy the probiotics. Afterwards, each component of artilysins can be expressed separately, and the bacteriolytic effect of each component will be tested. To further purify artilysins, obtain the minimum inhibitory concentration (MIC) and the minimum bactericidal concentration (MBC) by tube dilution method and the viable bacteria counting method. In addition, cytotoxicity tests and in vivo bacteriolytic assay of artilysins are required.

## Supplementary Information

**Additional file 1: Table S1.** Components of artilysins. **Table S2.** Sequence of LPS-destabilizing activity Peptides. **File S1.** The sequence of artilysins. **Table S3.** High-density fermentation data record sheet.

## Data Availability

All vectors generated in this study can be obtained from the corresponding author. Not applicable.
