# Supplementary material for: Engineered endolysin-based “artilysins” for controlling the gram-negative pathogen Helicobacter pylori
Source: AMB Express. 2021 Apr 28;11:63. doi: 10.1186/s13568-021-01222-8 (PMC8081812; doi:10.1186/s13568-021-01222-8)
Supplement: Supplementary file 1 — Additional file 1: Table S1. Components of artilysins. Table S2. Sequence of LPS-destabilizing activity Peptides. File S1. The sequence of artilysins. Table S3. High-density fermentation data record sheet. [file 13568_2021_1222_MOESM1_ESM.pdf]

Supplementary Material

Journal name:

AMB Express

Manuscript title:

Engineered Endolysin-based "Artilysins" for Controlling the Gram-negative Pathogen *Helicobacter pylori*

Authors names:

Dengyuan Xu [1,2], Shanshan Zhao [2], Jun Dou [1,2], Xiaofeng Xu [2], Yanyan Zhi [2], Liangzhu Wen [2,*]

Authors affiliation and address:

1 China Pharmaceutical University, Nanjing 211100, China

2 Wanbang Pharmatech Co., Ltd., Xuzhou 221004, China

Corresponding author:

Liangzhu Wen

E-Mail: wlz@wbpharma.com

Phone: 0086 18626008158

Supplement Table S1 Components of artilysins

Components of Artilysin	Protein_id	Amino acid sequence
LPS-destabilizing activity Peptides	Nothing	KRKKRKKRK(PCNP)
Holin A	YP_007237641.1	MQQHLVILGYETSKLIPYLLVATIGLFVGFLYVLRTIRPKELKNKTEKAFYIIQGVG
		SSMLITWISYEIADYFFKLPISLCVAISGGVGYLGSDSVSVLVLDILKKRL
Holin B	YP_007005685.1	MQQHLIILGYETSKIVPYILVMMIGLFVGFLYVLRAIRNEDFKNKTEKVFYIIQGV
		GSSMLITWISYEIENYFFNLPTSLCVAISGGVGYLGAERVSTLALDILKKRI
Endolysin A	YP_007237642.1	MTNLENALNNGNFKEQVYSSLEGVYQISKVLNQLDLLKNFSDHDLEINSFYQNK
		TANLRDGNKIVSTYRTTCIFETPSEQADYKIAVFARKHKDLWVNMNYTANTSGFE
		TSFLNNANFRGLTTQSIPTTYTTTGYFTSIPKRSFMKL
Endolysin B	YP_007005686.1	MDLTNLEDALNNGNFKEQVYSGLDGVYRISKVLNQLDLLKNFSEHDLEIVGGNG
		WVFHEHSQAIVYEILK

Supplement Table S2 Sequence of LPS-destabilizing activity Peptides

LPS-destabilizing activity Peptides	Polycationic nonapeptide (PCNP)
amino acid sequence	KRKKRKKRK
original nucleotide sequence	AAACGTAAAAAAACGTAAAAAAACGTAAA
optimized nucleotide sequence	AAACGTAAGAAACGTAAGAAACGTAAA

Supplement File S1 The sequence of artilysins

Artilysin 1 (Nucleotide sequence):

GTGGACGATGACGATAAGAAACGTAAGAAACGTAAGAAACGTAAAGGCGCGGGCGCGATGCAGCAACACCTGGTTATCCTGGGTTACGAGACCAGCAAGCTGATCCCGT

ACCTGCTGGTTGCGACCATTGGTCTGTTCGTTGGCTTTCTGTACGTGCTGCGTACCATTCGTCCGAAGGAGCTGAAGAACAAAACCGAAAAAGCGTTCTATATCATTCAGGG

TGTTGGCAGCAGCATGCTGATCACCTGGATTAGCTACGAAATCGCGGACTATTTCTTTAAGCTGCCGATCAGCCTGTGCGTTGCGATTAGCGGTGGCGTGGGTTACCTGGG

CAGCGACAGCGTGAGCGTTCTGGTGCTGGATATTCTGAAGAAACGTCTGGGTGCGGGCGCGATGACCAACCTGGAGAACGCGCTGAACAACGGTAACTTTAAAGAGCAA

GTTTACAGCAGCCTGGAAGGCGTTTATCAGATCAGCAAGGTGCTGAACCAACTGGACCTGCTGAAAAACTTCAGCGACCACGATCTGGAAATTAACAGCTTTTACCAGAA

CAAGACCGCGAACCTGCGTGATGGTAACAAAATCGTGAGCACCTACCGTACCACCTGCATTTTCGAGACCCCGAGCGAACAAGCGGACTATAAAATCGCGGTTTTTGCGCG

TAAGCACAAAGATCTGTGGGTGAACATGAACTATACCGCGAACACCAGCGGTTTCGAGACCAGCTTTCTGAACAACGCGAACTTCCGTGGCCTGACCACCCAGAGCATCC

CGACCACCTACACCACCACCGGCTATTTCACCAGC

ATTCCGAAGCGTAGCTTTATGAAACTGTAA

Artilysin 1 (amino acid sequence):

KRKKRKKRKGAGAMQQHLVILGYETSKLIPYLLVATIGLFVGFLYVLRTIRPKELKNKTEKAFYIIQGVGSSMLITWISYEIADYFFKLPISLCVAISGGVGYLGSDSVSVLVLDILKKRLG

AGAMTNLENALNNGNFKEQVYSSLEGVYQISKVLNQLDLLKNFSDHDLEINSFYQNKTANLRDGNKIVSTYRTTCIFETPSEQADYKIAVFARKHKDLWVNMNYTANTSGFETSF

LNNANFRGLTTQSIPTTYTTTGYFTSIPKRSFMKL*

Artilysin 2 (Nucleotide sequence):

GTGGACGATGACGATAAGAAACGTAAGAAACGTAAGAAACGTAAAGGCGCGGGCGCGATGCAGCAACACCTGATCATTCTGGGTTACGAGACCAGCAAAATCGTGCCGT

ATATTCTGGTTATGATGATCGGTCTGTTCGTGGGCTTTCTGTACGTTCTGCGTGCGATTCGTAACGAGGACTTCAAGAACAAGACCGAAAAGGTGTTTTATATCATCCAGGGT

GTTGGCAGCAGCATGCTGATCACCTGGATTAGCTACGAGATCGAAAACTATTTCTTTAACCTGCCGACCAGCCTGTGCGTGGCGATTAGCGGTGGCGTTGGTTATCTGGGTG

CGGAACGTGTGAGCACCCTGGCGCTGGACATCCTGAAGAAA

CGTATTGGTGCGGGCGCGATGGACCTGACCAACCTGGAGGATGCGCTGAACAACGGTAACTTCAAGGAACAGGTTTACAGCGGTCTGGATGGCGTGTATCGTATCAGCAA

GGTTCTGAACCAACTGGACCTGCTGAAAAACTTCAGCGAGCACGATCTGGAAATCGTGGGTGGCAACGGCTGGGTTTTTCACGAGCACAGCCAAGCGATCGTTTATGAAA

TTCTGAAATAA

Artilysin 2 (amino acid sequence):

KRKKRKKRKGAGAMQQHLIILGYETSKIVPYILVMMIGLFVGFLYVLRAIRNEDFKNKTEKVFYIIQGVGSSMLITWISYEIENYFFNLPTSLCVAISGGVGYLGAERVSTLALDILKKR

IGAGAMDLTNLEDALNNGNFKEQVYSGLDGVYRISKVLNQLDLLKNFSEHDLEIVGGNGWVFHEHSQAIVYEILK*

Supplement Table S3 High-density fermentation data record sheet

MCGS_Time	MCGS_TIMEMS	RUNTIME1	Temperature(℃)	pH	DO(%)	Agitator processvalue(rpm)	Acid volume	Lye volume	Feed volume	Defoamer volume
2019/8/15 13:50	366	0	36.8	6.96885	57.6523	200	1.42	10	0	0
2019/8/15 13:50	665	0.01	36.8	6.96885	57.3551	200	1.42	10	0	0
2019/8/15 13:51	748	0.02	36.8	6.96885	56.6122	200	1.42	10	0	0
2019/8/15 13:52	831	0.03	36.7	6.96885	56.0178	200	1.42	10	0	0
2019/8/15 13:52	126	0.04	36.9	7.05191	55.7206	200	1.42	10.8401	0	0
2019/8/15 13:53	216	0.05	37	7.06229	55.2749	200	1.42	10.8401	0	0
2019/8/15 13:53	300	0.06	37	7.05191	54.3834	200	1.42	10.8401	0	0
2019/8/15 13:54	49	0.07	37.1	7.05191	53.4918	200	1.42	10.8401	0	0
2019/8/15 13:55	385	0.08	37.1	7.05191	52.6003	200	1.42	10.8401	0	0
2019/8/15 13:55	471	0.09	37	7.05191	52.0059	200	1.42	10.8401	0	0
2019/8/15 13:56	556	0.1	37	7.04153	51.1144	200	1.42	10.8401	0	0
2019/8/15 13:56	642	0.11	37	7.04153	50.3715	200	1.42	10.8401	0	0
2019/8/15 13:57	727	0.12	36.9	7.04153	49.6285	200	1.42	10.8401	0	0
2019/8/15 13:58	812	0.13	36.9	7.04153	49.3313	200	1.42	10.8401	0	0
2019/8/15 13:58	675	0.14	36.9	7.04153	47.8455	200	1.42	10.8401	0	0
2019/8/15 13:59	978	0.15	36.9	7.03115	46.9539	200	1.42	10.8401	0	0
2019/8/15 13:59	57	0.16	36.8	7.03115	46.3596	200	1.42	10.8401	0	0
2019/8/15 14:00	141	0.17	36.8	7.03115	44.5765	200	1.42	10.8401	0	0
2019/8/15 14:01	224	0.18	36.7	7.03115	44.1308	200	1.42	10.8401	0	0
2019/8/15 14:01	306	0.19	36.8	7.03115	43.3878	200	1.42	10.8401	0	0
2019/8/15 14:02	390	0.2	36.9	7.03115	42.7935	200	1.42	10.8401	0	0
2019/8/15 14:02	476	0.21	37	7.02076	41.4562	200	1.42	10.8401	0	0
2019/8/15 14:03	557	0.22	37	7.02076	39.5245	200	1.42	10.8401	0	0
2019/8/15 14:04	642	0.23	37	7.02076	38.3358	200	1.42	10.8401	0	0
2019/8/15 14:04	726	0.24	37	7.02076	36.7013	200	1.42	10.8401	0	0
2019/8/15 14:05	816	0.25	37	7.02076	35.6612	200	1.42	10.8401	0	0
2019/8/15 14:05	897	0.26	37	7.02076	34.7697	200	1.42	10.8401	0	0
2019/8/15 14:06	977	0.27	37	7.02076	34.6211	200	1.42	10.8401	0	0
2019/8/15 14:07	65	0.28	37	7.01038	34.4725	200	1.42	10.8401	0	0
2019/8/15 14:07	148	0.29	36.9	7.01038	32.9866	200	1.42	10.8401	0	0
2019/8/15 14:08	231	0.3	36.9	7.02076	31.7979	200	1.42	10.8401	0	0

2019/8/15 14:08	313	0.31	36.8	7.02076	31.2036	200	1.42	10.8401	0	0
2019/8/15 14:09	404	0.32	36.8	7.01038	30.6092	200	1.42	10.8401	0	0
2019/8/15 14:10	490	0.33	36.7	7.01038	30.0149	200	1.42	10.8401	0	0
2019/8/15 14:10	573	0.34	36.8	7.01038	28.529	200	1.42	10.8401	0	0
2019/8/15 14:11	661	0.35	36.9	7.01038	27.9346	200	1.42	10.8401	0	0
2019/8/15 14:11	742	0.36	37	7.01038	27.9346	200	1.42	10.8401	0	0
2019/8/15 14:12	825	0.37	37	7.01038	25.7058	200	1.42	10.8401	0	0
2019/8/15 14:13	908	0.38	37.1	7.01038	25.1114	200	1.42	10.8401	0	0
2019/8/15 14:13	996	0.39	37	7	24.9629	250	1.42	10.8401	0	0
2019/8/15 14:14	78	0.4	37	7	32.0951	250	1.42	10.8401	0	0
2019/8/15 14:14	165	0.41	37	7.01038	40.416	250	1.42	10.8401	0	0
2019/8/15 14:15	247	0.42	36.9	7.01038	45.9138	250	1.42	10.8401	0	0
2019/8/15 14:16	330	0.43	36.9	7	48.737	250	1.42	10.8401	0	0
2019/8/15 14:16	410	0.44	36.9	7	50.3715	250	1.42	10.8401	0	0
2019/8/15 14:17	490	0.45	36.8	7	50.8172	250	1.42	10.8401	0	0
2019/8/15 14:17	574	0.46	36.8	7.01038	50.5201	250	1.42	10.8401	0	0
2019/8/15 14:18	658	0.47	36.7	7	50.8172	250	1.42	10.8401	0	0
2019/8/15 14:19	740	0.48	36.7	7	50.3715	250	1.42	10.8401	0	0
2019/8/15 14:19	819	0.49	36.9	7.01038	50.3715	250	1.42	10.8401	0	0
2019/8/15 14:20	654	0.5	37	7	48.737	250	1.42	10.8401	0	0
2019/8/15 14:20	988	0.51	37	7	47.3997	250	1.42	10.8401	0	0
2019/8/15 14:21	73	0.52	37.1	7	46.3596	250	1.42	10.8401	0	0
2019/8/15 14:22	157	0.53	37	7	45.6166	250	1.42	10.8401	0	0
2019/8/15 14:22	249	0.54	37	7	44.2793	250	1.54	10.8401	0	0
2019/8/15 14:23	343	0.55	37	6.95847	42.7935	250	1.9	10.8401	0	0
2019/8/15 14:23	277	0.56	37	6.92733	42.1991	250	1.9	10.8401	0	0
2019/8/15 14:24	357	0.57	37	6.92733	40.416	250	1.9	10.8401	0	0
2019/8/15 14:25	595	0.58	37	6.92733	39.8217	250	1.9	10.8401	0	0
2019/8/15 14:25	682	0.59	36.9	6.92733	38.633	250	1.9	10.8401	0	0
2019/8/15 14:26	764	0.6	36.9	6.92733	37.7415	250	1.9	10.8401	0	0
2019/8/15 14:26	843	0.61	36.8	6.92733	37.1471	250	1.9	10.8401	0	0
2019/8/15 14:27	928	0.62	36.8	6.92733	36.107	250	1.9	10.8401	0	0
2019/8/15 14:28	12	0.63	36.8	6.92733	35.6612	250	1.9	10.8401	0	0
2019/8/15 14:28	99	0.64	36.8	6.92733	34.0267	250	1.9	10.8401	0	0
2019/8/15 14:29	185	0.65	36.9	6.92733	31.6493	250	1.9	10.8401	0	0

2019/8/15 14:29	269	0.66	37	6.92733	30.1634	250	1.9	10.8401	0	0
2019/8/15 14:30	358	0.67	37.1	6.92733	29.2719	250	1.9	10.8401	0	0
2019/8/15 14:31	443	0.68	37.1	6.92733	27.4889	250	1.9	10.8401	0	0
2019/8/15 14:31	282	0.69	37.1	6.92733	26.4487	250	1.9	10.8401	0	0
2019/8/15 14:32	291	0.7	37.1	6.92733	24.6657	300	1.9	10.8401	0	0
2019/8/15 14:32	675	0.71	37	6.92733	31.055	300	1.9	10.8401	0	0
2019/8/15 14:33	762	0.72	37	6.92733	41.3076	300	1.9	10.8401	0	0
2019/8/15 14:34	847	0.73	37	6.92733	45.9138	300	1.9	10.8401	0	0
2019/8/15 14:34	934	0.74	36.9	6.92733	48.4398	300	1.9	10.8401	0	0
2019/8/15 14:35	40	0.75	36.9	6.92733	49.0342	300	1.9	10.8401	0	0
2019/8/15 14:35	122	0.76	36.9	6.92733	48.4398	300	1.9	10.8401	0	0
2019/8/15 14:36	211	0.77	36.8	6.92733	48.1426	300	1.9	10.8401	0	0
2019/8/15 14:37	299	0.78	36.7	6.92733	47.2511	300	1.9	10.8401	0	0
2019/8/15 14:37	379	0.79	36.7	6.92733	46.0624	300	1.9	10.8401	0	0
2019/8/15 14:38	462	0.8	36.9	6.92733	44.5765	300	1.9	10.8401	0	0
2019/8/15 14:38	550	0.81	37	6.92733	44.5765	300	1.9	10.8401	0	0
2019/8/15 14:39	636	0.82	37	6.92733	43.5364	300	1.9	10.8401	0	0
2019/8/15 14:40	734	0.83	37	6.92733	41.4562	300	1.9	10.8401	0	0
2019/8/15 14:40	816	0.839999	37	6.91694	41.159	300	1.9	10.8401	0	0
2019/8/15 14:41	906	0.849999	37	6.92733	40.5646	300	1.9	10.8401	0	0
2019/8/15 14:41	991	0.859999	37	6.92733	39.9703	300	1.9	10.8401	0	0
2019/8/15 14:42	75	0.869999	37	6.91694	40.2675	300	1.9	10.8401	0	0
2019/8/15 14:43	163	0.879999	36.9	6.92733	41.4562	300	1.9	10.8401	0	0
2019/8/15 14:43	245	0.889999	36.9	6.91694	42.6449	300	1.9	10.8401	0	0
2019/8/15 14:44	331	0.899999	36.9	6.92733	44.1308	300	1.9	10.8401	0	0
2019/8/15 14:44	414	0.909999	36.8	6.91694	45.3195	300	1.9	10.8401	0	0
2019/8/15 14:45	500	0.919999	36.7	6.92733	45.6166	300	1.9	10.8401	0	0
2019/8/15 14:46	583	0.929999	36.7	6.92733	45.3195	300	1.9	10.8401	0	0
2019/8/15 14:46	669	0.939999	36.9	6.92733	45.0223	300	1.9	10.8401	0	0
2019/8/15 14:47	753	0.949999	37	6.92733	42.1991	300	1.9	10.8401	0	0
2019/8/15 14:47	836	0.959999	37	6.91694	38.7816	300	1.9	10.8401	0	0
2019/8/15 14:48	917	0.969999	37	6.92733	34.6211	300	1.9	10.8401	0	0
2019/8/15 14:49	2	0.979999	37	6.92733	31.2036	300	1.9	10.8401	0	0
2019/8/15 14:49	87	0.989999	37	6.91694	26.8945	300	1.9	10.8401	0	0
2019/8/15 14:50	165	0.999999	37	6.91694	24.8143	300	1.9	10.8401	0	0

2019/8/15 14:50	250	1.01	36.9	6.91694	23.9227	300	1.9	10.8401	0	0
2019/8/15 14:51	333	1.02	36.9	6.91694	23.3284	300	1.9	10.8401	0	0
2019/8/15 14:52	415	1.03	36.9	6.91694	21.3967	300	1.9	10.8401	0	0
2019/8/15 14:52	499	1.04	36.8	6.92733	21.2481	300	1.9	10.8401	0	0
2019/8/15 14:53	363	1.05	36.8	6.91694	22.1397	350	1.9	10.8401	0	0
2019/8/15 14:53	666	1.06	36.7	6.92733	34.0267	350	1.9	10.8401	0	0
2019/8/15 14:54	755	1.07	36.8	6.92733	40.8618	350	1.9	10.8401	0	0
2019/8/15 14:55	657	1.08	36.9	6.92733	42.7935	350	1.9	10.8401	0	0
2019/8/15 14:55	914	1.09	37	6.92733	41.7533	350	1.9	10.8401	0	0
2019/8/15 14:56	8	1.1	37	6.92733	41.4562	350	1.9	10.8401	0	0
2019/8/15 14:56	87	1.11	37	6.92733	39.2273	350	1.9	10.8401	0	0
2019/8/15 14:57	175	1.12	37	6.92733	37.5929	350	1.9	10.8401	0	0
2019/8/15 14:58	260	1.13	37	6.92733	36.2556	350	1.9	10.8401	0	0
2019/8/15 14:58	353	1.14	36.9	6.92733	35.6612	350	1.9	10.8401	0	0
2019/8/15 14:59	438	1.15	36.9	6.92733	34.6211	350	1.9	10.8401	0	0
2019/8/15 14:59	525	1.16	36.9	6.92733	33.8782	350	1.9	10.8401	0	0
2019/8/15 15:00	609	1.17	36.8	6.92733	32.5409	350	1.9	10.8401	0	0
2019/8/15 15:01	696	1.18	36.8	6.92733	31.2036	350	1.9	10.8401	0	0
2019/8/15 15:01	778	1.19	36.8	6.92733	30.6092	350	1.9	10.8401	0	0
2019/8/15 15:02	861	1.2	36.8	6.92733	28.9747	350	1.9	10.8401	0	0
2019/8/15 15:02	948	1.21	36.9	6.92733	27.4889	350	1.9	10.8401	0	0
2019/8/15 15:03	34	1.22	37	6.92733	26.4487	400	1.9	10.8401	0	0
2019/8/15 15:04	116	1.23	37	6.92733	37.2957	400	1.9	10.8401	0	0
2019/8/15 15:04	193	1.24	37	6.92733	42.3477	400	1.9	10.8401	0	0
2019/8/15 15:05	283	1.25	37	6.92733	42.942	400	1.9	10.8401	0	0
2019/8/15 15:05	363	1.26	37	6.92733	43.9822	400	1.9	10.8401	0	0
2019/8/15 15:06	450	1.27	36.9	6.92733	42.6449	400	1.9	10.8401	0	0
2019/8/15 15:07	535	1.28	36.9	6.92733	41.7533	400	1.9	10.8401	0	0
2019/8/15 15:07	619	1.29	36.9	6.92733	40.5646	400	1.9	10.8401	0	0
2019/8/15 15:08	703	1.3	36.8	6.92733	39.5245	400	1.9	10.8401	0	0
2019/8/15 15:08	785	1.31	36.8	6.92733	38.1872	400	1.9	10.8401	0	0
2019/8/15 15:09	874	1.32	36.7	6.92733	36.9985	400	1.9	10.8401	0	0
2019/8/15 15:10	954	1.33	36.8	6.92733	36.4042	400	1.9	10.8401	0	0
2019/8/15 15:10	35	1.34	37	6.92733	35.364	400	1.9	10.8401	0	0
2019/8/15 15:11	121	1.35	37	6.92733	33.4324	400	1.9	10.8401	0	0

2019/8/15 15:11	206	1.36	37	6.92733	31.3522	400	1.9	10.8401	0	0
2019/8/15 15:12	289	1.37	37	6.92733	30.1634	400	1.9	10.8401	0	0
2019/8/15 15:13	373	1.38	37	6.92733	29.8663	400	1.9	10.8401	0	0
2019/8/15 15:13	457	1.39	37	6.92733	27.6374	400	1.9	10.8401	0	0
2019/8/15 15:14	541	1.4	36.9	6.92733	27.0431	400	1.9	10.8401	0	0
2019/8/15 15:14	624	1.41	36.9	6.92733	25.8544	400	1.9	10.8401	0	0
2019/8/15 15:15	707	1.42	36.8	6.92733	24.9629	450	1.9	10.8401	0	0
2019/8/15 15:16	788	1.43	36.8	6.92733	33.1352	450	1.9	10.8401	0	0
2019/8/15 15:16	873	1.44	36.8	6.92733	38.7816	450	1.9	10.8401	0	0
2019/8/15 15:17	959	1.45	36.8	6.92733	39.9703	450	1.9	10.8401	0	0
2019/8/15 15:17	40	1.46	36.9	6.92733	39.6731	450	1.9	10.8401	0	0
2019/8/15 15:18	127	1.47	37	6.91694	38.4844	450	1.9	10.8401	0	0
2019/8/15 15:19	208	1.48	37	6.91694	36.9985	450	1.9	10.8401	0	0
2019/8/15 15:19	298	1.49	37	6.91694	36.2556	450	1.9	10.8401	0	0
2019/8/15 15:20	389	1.5	37	6.91694	35.8098	450	1.9	10.8401	0	0
2019/8/15 15:20	477	1.51	37	6.91694	34.4725	450	1.9	10.8401	0	0
2019/8/15 15:21	560	1.52	37	6.91694	32.838	450	1.9	10.8401	0	0
2019/8/15 15:22	645	1.53	37	6.91694	31.9465	450	1.9	10.8401	0	0
2019/8/15 15:22	729	1.54	36.9	6.91694	31.3522	450	1.9	10.8401	0	0
2019/8/15 15:23	817	1.55	36.9	6.91694	30.312	450	1.9	10.8401	0	0
2019/8/15 15:23	901	1.56	36.8	6.91694	29.5691	450	1.9	10.8401	0	0
2019/8/15 15:24	980	1.57	36.8	6.91694	28.6776	450	1.9	10.8401	0	0
2019/8/15 15:25	59	1.58	36.7	6.91694	27.4889	450	1.9	10.8401	0	0
2019/8/15 15:25	147	1.59	36.8	6.91694	26.3001	450	1.9	10.8401	0	0
2019/8/15 15:26	235	1.6	36.9	6.91694	24.2199	450	1.9	10.8401	0	0
2019/8/15 15:26	318	1.61	37	6.91694	28.9747	500	1.9	10.8401	0	0
2019/8/15 15:27	404	1.62	37	6.91694	35.2155	500	1.9	10.8401	0	0
2019/8/15 15:28	489	1.63	37	6.91694	35.0669	500	1.9	10.8401	0	0
2019/8/15 15:28	576	1.64	37	6.91694	34.4725	500	1.9	10.8401	0	0
2019/8/15 15:29	358	1.65	37	6.91694	32.838	500	1.9	10.8401	0	0
2019/8/15 15:29	707	1.66	37	6.91694	31.6493	500	1.9	10.8401	0	0
2019/8/15 15:30	788	1.67	37	6.91694	31.055	500	1.9	10.8401	0	0
2019/8/15 15:31	871	1.68	36.9	6.91694	28.529	500	1.9	10.8401	0	0
2019/8/15 15:31	997	1.69	36.9	6.91694	27.9346	500	1.9	10.8401	0	0
2019/8/15 15:32	82	1.7	36.9	6.91694	26.7459	550	1.9	10.8401	0	0

2019/8/15 15:32	174	1.71	36.9	6.91694	33.2838	550	1.9	10.8401	0	0
2019/8/15 15:33	259	1.72	36.9	6.91694	38.1872	550	1.9	10.8401	0	0
2019/8/15 15:34	349	1.73	36.8	6.91694	38.9302	550	1.9	10.8401	0	0
2019/8/15 15:34	430	1.74	36.8	6.92733	38.4844	550	1.9	10.8401	0	0
2019/8/15 15:35	520	1.75	36.8	6.92733	37.89	550	1.9	10.8401	0	0
2019/8/15 15:35	602	1.76	36.8	6.92733	37.2957	550	1.9	10.8401	0	0
2019/8/15 15:36	684	1.77	36.8	6.92733	36.107	550	1.9	10.8401	0	0
2019/8/15 15:37	766	1.78	36.8	6.92733	35.5126	550	1.9	10.8401	0	0
2019/8/15 15:37	847	1.79	36.8	6.92733	34.6211	550	1.9	10.8401	0	0
2019/8/15 15:38	932	1.8	36.8	6.92733	33.8782	550	1.9	10.8401	0	0
2019/8/15 15:38	17	1.81	37	6.92733	32.5409	550	1.9	10.8401	0	0
2019/8/15 15:39	100	1.82	37.1	6.92733	30.7578	550	1.9	10.8401	0	0
2019/8/15 15:40	183	1.83	37.1	6.92733	28.6776	550	1.9	10.8401	0	0
2019/8/15 15:40	264	1.84	37.1	6.92733	26.8945	550	1.9	10.8401	0	0
2019/8/15 15:41	347	1.85	37.2	6.92733	25.5572	600	1.9	10.8401	0	0
2019/8/15 15:41	431	1.86	37.2	6.92733	33.7296	600	1.9	10.8401	0	0
2019/8/15 15:42	516	1.87	37.2	6.92733	38.7816	600	1.9	10.8401	0	0
2019/8/15 15:43	598	1.88	37.2	6.92733	39.9703	600	1.9	10.8401	0	0
2019/8/15 15:43	684	1.89	37.2	6.93771	40.1189	600	1.9	10.8401	0	0
2019/8/15 15:44	766	1.9	37.2	6.93771	39.0788	600	1.9	10.8401	0	0
2019/8/15 15:44	857	1.91	37.1	6.93771	38.7816	600	1.9	10.8401	0	0
2019/8/15 15:45	945	1.92	37.2	6.93771	38.1872	600	1.9	10.8401	0	0
2019/8/15 15:46	39	1.93	37.1	6.93771	37.5929	600	1.9	10.8401	0	0
2019/8/15 15:46	124	1.94	37.1	6.93771	36.7013	600	1.92	10.8401	0	0
2019/8/15 15:47	210	1.95	37.1	6.93771	36.2556	600	1.92	10.8401	0	0
2019/8/15 15:47	296	1.96	37.1	6.93771	34.9183	600	1.92	10.8401	0	0
2019/8/15 15:48	376	1.97	37.1	6.93771	34.0267	600	1.96	10.8401	0	0
2019/8/15 15:49	458	1.98	37.1	6.93771	32.838	600	1.96	10.8401	0	0
2019/8/15 15:49	539	1.99	37.1	6.93771	32.2437	600	1.96	10.8401	0	0
2019/8/15 15:50	625	2	37.1	6.93771	30.7578	600	1.96	10.8401	0	0
2019/8/15 15:50	717	2.01	37.1	6.93771	29.2719	600	1.96	10.8401	0	0
2019/8/15 15:51	801	2.02	37.1	6.93771	27.1917	650	1.98	10.8401	0	0
2019/8/15 15:52	884	2.03	37.1	6.93771	34.0267	650	1.98	10.8401	0	0
2019/8/15 15:52	686	2.04	37	6.93771	36.8499	650	0	0	0	0
2019/8/15 15:53	52	2.05	37	6.93771	36.8499	650	0	0	0	0

2019/8/15 15:53	135	2.06	37	6.93771	35.364	650	0	0	0	0
2019/8/15 15:54	221	2.07	37	6.93771	33.7296	650	0	0	0	0
2019/8/15 15:55	307	2.08	37	6.93771	32.0951	650	0	0	0	0
2019/8/15 15:55	392	2.09	37.1	6.93771	30.6092	650	0	0	0	0
2019/8/15 15:56	480	2.1	37.1	6.93771	29.1233	650	0	0	0	0
2019/8/15 15:56	564	2.11	37.1	6.93771	35.5126	700	0	0	0	0
2019/8/15 15:57	634	2.12	37.1	6.93771	37.2957	700	0.04	0	0	0
2019/8/15 15:58	653	2.13	37.1	6.92733	36.7013	700	0.2	0	0	0
2019/8/15 15:58	740	2.14	37.2	6.92733	36.2556	700	0.2	0	0	0
2019/8/15 15:59	829	2.15	37.1	6.92733	34.3239	700	0.2	0	0	0
2019/8/15 15:59	0	2.16	37.2	6.93771	32.5409	700	0.2	0	0	0
2019/8/15 16:00	83	2.17	37.2	6.93771	31.3522	700	0.2	0	0	0
2019/8/15 16:01	164	2.18	37.2	6.93771	29.5691	700	0.2	0	0	0
2019/8/15 16:01	0	2.19	37.2	6.93771	28.3804	750	0.2	0	0	0
2019/8/15 16:02	341	2.2	37.2	6.93771	34.4725	750	0.2	0	0	0
2019/8/15 16:02	421	2.21	37.3	6.93771	35.8098	750	0.22	0	0	0
2019/8/15 16:03	502	2.22	37.3	6.93771	35.2155	750	0.22	0	0	0
2019/8/15 16:04	357	2.23	37.2	6.92733	34.0267	750	0.36	0	0	0
2019/8/15 16:04	686	2.24	37.3	6.93771	32.5409	750	0.36	0	0	0
2019/8/15 16:05	768	2.25	37.3	6.93771	31.6493	750	0.36	0	0	0
2019/8/15 16:05	854	2.26	37.3	6.93771	30.312	750	0.36	0	0	0
2019/8/15 16:06	935	2.27	37.4	6.93771	28.9747	750	0.36	0	0	0
2019/8/15 16:07	19	2.28	37.4	6.93771	27.786	750	0.48	0	0	0
2019/8/15 16:07	104	2.29	37.4	6.92733	26.8945	750	0.48	0	0	0
2019/8/15 16:08	187	2.3	37.4	6.93771	25.1114	750	0.48	0	0	0
2019/8/15 16:08	123	2.31	37.4	6.93771	30.312	800	0.48	0	0	0
2019/8/15 16:09	357	2.32	37.5	6.93771	32.2437	800	0.48	0	0	0
2019/8/15 16:10	440	2.33	37.4	6.93771	31.6493	800	0.48	0	0	0
2019/8/15 16:10	527	2.34	37.5	6.93771	30.7578	800	0.54	0	0	0
2019/8/15 16:11	612	2.35	37.5	6.93771	28.9747	800	0.54	0	0	0
2019/8/15 16:11	697	2.36	37.5	6.93771	28.0832	800	0.54	0	0	0
2019/8/15 16:12	776	2.37	37.5	6.92733	26.5973	800	0.64	0	0	0
2019/8/15 16:13	860	2.38	37.4	6.92733	25.4086	800	0.64	0	0	0
2019/8/15 16:13	948	2.39	37.4	6.92733	25.4086	800	0.64	0	0	0
2019/8/15 16:14	31	2.4	37.5	6.93771	35.9584	850	0.64	0	0	0

2019/8/15 16:14	119	2.41	37.4	6.93771	41.7533	850	0.82	0	0	0
2019/8/15 16:15	200	2.42	37.4	6.93771	42.3477	850	0.82	0	0	0
2019/8/15 16:16	286	2.43	37.4	6.93771	40.8618	850	0.94	0	0	0
2019/8/15 16:16	368	2.44	37.4	6.93771	39.6731	850	0.94	0	0	0
2019/8/15 16:17	457	2.45	37.4	6.93771	37.89	850	1.08	0	0	0
2019/8/15 16:17	545	2.46	37.4	6.93771	36.5527	850	1.08	0	0	0
2019/8/15 16:18	628	2.47	37.4	6.93771	34.0267	850	1.2	0	0	0
2019/8/15 16:19	713	2.48	37.4	6.93771	31.5007	850	1.2	0	0	0
2019/8/15 16:19	908	2.49	37.4	6.93771	28.3804	850	1.3	0	0	0
2019/8/15 16:20	918	2.5	37.4	6.93771	25.8544	850	1.3	0	0	0
2019/8/15 16:20	668	2.51	37.3	6.94809	22.5854	850	1.3	0	0	0
2019/8/15 16:21	47	2.52	37.4	6.93771	24.3685	900	1.46	0	0	0
2019/8/15 16:22	127	2.53	37.3	6.93771	25.1114	900	1.46	0	0	0
2019/8/15 16:22	212	2.54	37.3	6.93771	22.8826	900	1.6	0	0	0
2019/8/15 16:23	306	2.55	37.4	6.93771	22.2883	950	1.6	0	0	0
2019/8/15 16:23	388	2.56	37.3	6.92733	22.734	900	1.72	0	0	0
2019/8/15 16:24	470	2.57	37.3	6.93771	19.3165	900	1.72	0	0	0
2019/8/15 16:25	548	2.58	37.3	6.93771	16.1961	900	1.9	0	0	0
2019/8/15 16:25	629	2.59	37.3	6.93771	13.9673	900	1.9	0	0	0
2019/8/15 16:26	717	2.6	37.3	6.93771	11.2927	900	1.9	0	0	0
2019/8/15 16:26	803	2.61	37.3	6.93771	8.9153	900	2.02	0	0	0
2019/8/15 16:27	885	2.62	37.3	6.93771	6.24071	900	2.02	0	0	0
2019/8/15 16:28	972	2.63	37.3	6.93771	4.45765	950	2.14	0	0	0
2019/8/15 16:28	55	2.64	37.3	6.93771	8.76672	950	2.14	0	0	0
2019/8/15 16:29	142	2.65	37.3	6.93771	8.61813	950	2.14	0	0	0
2019/8/15 16:29	221	2.66	37.3	6.93771	7.13224	950	2.3	0	0	0
2019/8/15 16:30	308	2.67	37.3	6.93771	5.94354	950	2.3	0	0	0
2019/8/15 16:31	391	2.68	37.3	6.93771	5.20059	950	2.34	0	0	0
2019/8/15 16:31	424	2.69	37.3	6.93771	4.01189	950	2.48	0	0	0
2019/8/15 16:32	509	2.7	37.3	6.93771	3.71471	950	2.48	0	0	0
2019/8/15 16:32	600	2.71	37.2	6.94809	3.12036	950	2.5	0	0	0
2019/8/15 16:33	733	2.72	37.2	6.93771	2.37741	950	2.64	0	0	0
2019/8/15 16:34	812	2.73	37.2	6.93771	2.08024	950	2.64	0	0	0
2019/8/15 16:34	906	2.74	37.2	6.93771	1.78306	950	2.76	0	0	0
2019/8/15 16:35	993	2.75	37.2	6.93771	1.48588	950	2.76	0	0	0

2019/8/15 16:35	75	2.76	37.2	6.92733	1.18871	950	2.88	0	0	0
2019/8/15 16:36	164	2.77	37.2	6.93771	0.89153	950	2.88	0	0	0
2019/8/15 16:37	248	2.78	37.2	6.93771	0.89153	950	2.88	0	0	0
2019/8/15 16:37	336	2.79	37.2	6.93771	0.594354	950	3.02	0	0	0
2019/8/15 16:38	420	2.8	37.2	6.93771	0.445765	950	3.02	0	0	0
2019/8/15 16:38	504	2.81	37.2	6.93771	0.445765	950	3.02	0	0	0
2019/8/15 16:39	596	2.82	37.2	6.93771	0.445765	950	3.02	0	0	0
2019/8/15 16:40	678	2.83	37.2	6.93771	0.445765	950	3.14	0	0	0
2019/8/15 16:40	761	2.84	37.3	6.93771	0.148588	950	3.14	0	0	0
2019/8/15 16:41	850	2.85	37.2	6.93771	0.148588	950	3.14	0	0	0
2019/8/15 16:41	153	2.86	37.2	6.93771	0.148588	950	3.14	0	0	0
2019/8/15 16:42	240	2.87	37.2	6.93771	0.148588	950	3.24	0	0	0
2019/8/15 16:43	321	2.88	37.2	6.93771	0.148588	950	3.24	0	0	0
2019/8/15 16:43	406	2.89	37.2	6.93771	0.148588	950	3.24	0	0	0
2019/8/15 16:44	491	2.9	37.1	6.92733	0.148588	950	3.38	0	0	0
2019/8/15 16:44	576	2.91	37.2	6.92733	0.148588	950	3.38	0	0	0
2019/8/15 16:45	659	2.92	37.2	6.92733	0.148588	950	3.38	0	0	0
2019/8/15 16:46	745	2.93	37.2	6.93771	0.148588	950	3.38	0	0	0
2019/8/15 16:46	828	2.94	37.2	6.93771	0.148588	950	3.38	0	0	0
2019/8/15 16:47	910	2.95	37.2	6.93771	0.148588	950	3.38	0	0	0
2019/8/15 16:47	995	2.96	37.2	6.92733	0.148588	950	3.5	0	0	0
2019/8/15 16:48	80	2.97	37.3	6.92733	0.148588	950	3.5	0	0	0
2019/8/15 16:49	165	2.98	37.1	6.93771	0.148588	950	3.5	0	0	0
2019/8/15 16:49	250	2.99	37.2	6.93771	0.148588	950	3.5	0	0	0
2019/8/15 16:50	333	3	37.2	6.93771	0.148588	950	3.5	0	0	0
2019/8/15 16:50	423	3.01	37.2	6.93771	0.148588	950	3.5	0	0	0
2019/8/15 16:51	512	3.02	37.1	6.93771	0.148588	950	3.5	0	0	0
2019/8/15 16:52	394	3.03	37.3	6.93771	0.148588	950	3.5	0	0	0
2019/8/15 16:52	677	3.04	37.2	6.93771	0.148588	950	3.5	0	0	0
2019/8/15 16:53	758	3.05	37.2	6.93771	0.148588	950	3.5	0	0	0
2019/8/15 16:53	842	3.06	37.3	6.93771	0.148588	950	3.5	0	0	0
2019/8/15 16:54	925	3.07	37.2	6.93771	0.148588	950	3.5	0	0	0
2019/8/15 16:55	10	3.08	37.2	6.93771	0.148588	950	3.5	0	0	0
2019/8/15 16:55	91	3.09	37.2	6.93771	0.148588	950	3.5	0	0	0
2019/8/15 16:56	179	3.1	37.2	6.92733	0.148588	950	3.5	0	0	0

2019/8/15 16:56	259	3.11	37.3	6.92733	0.148588	950	3.5	0	0	0
2019/8/15 16:57	344	3.12	37.2	6.92733	0.148588	950	3.5	0	0	0
2019/8/15 16:58	430	3.13	37.1	6.92733	0.148588	950	3.5	0	0	0
2019/8/15 16:58	512	3.14	37.2	6.92733	0.148588	950	3.5	0	0	0
2019/8/15 16:59	301	3.15	37.2	6.92733	0.148588	950	3.5	0	0	0
2019/8/15 16:59	684	3.16	37.1	6.91694	0.148588	950	3.5	0	0	0
2019/8/15 17:00	766	3.17	37.2	6.91694	0.148588	950	3.5	0	0	0
2019/8/15 17:01	848	3.18	37.2	6.91694	0.148588	950	3.5	0	0	0
2019/8/15 17:01	934	3.19	37.1	6.91694	0.148588	950	3.5	0	0	0
2019/8/15 17:02	23	3.2	37.2	6.90656	0.148588	950	3.5	0	0	0
2019/8/15 17:02	105	3.21	37.2	6.90656	0.148588	950	3.5	0	0	0
2019/8/15 17:03	186	3.22	37.1	6.90656	0.148588	950	3.5	0	0	0
2019/8/15 17:04	268	3.23	37.2	6.90656	0.148588	950	3.5	0.06	0	0
2019/8/15 17:04	351	3.24	37.2	6.90656	0.148588	950	3.5	0.14	0	0
2019/8/15 17:05	438	3.25	37.1	6.90656	0.148588	950	3.5	0.14	0	0
2019/8/15 17:05	523	3.26	37.2	6.90656	0.148588	950	3.5	0.28	0	0
2019/8/15 17:06	604	3.27	37.2	6.90656	0.148588	950	3.5	0.28	0	0
2019/8/15 17:07	687	3.28	37.1	6.90656	0.148588	950	3.5	0.28	0	0
2019/8/15 17:07	768	3.29	37.2	6.90656	0.148588	950	3.5	0.34	0	0
2019/8/15 17:08	852	3.3	37.2	6.90656	0.148588	950	3.5	0.52	0	0
2019/8/15 17:08	940	3.31	37.1	6.90656	0.148588	950	3.5	0.52	0	0
2019/8/15 17:09	21	3.32	37.2	6.90656	0.148588	950	3.5	0.66	0	0
2019/8/15 17:10	109	3.33	37.2	6.90656	0.148588	950	3.5	0.68	0	0
2019/8/15 17:10	188	3.34	37.1	6.90656	0.148588	950	3.5	1.02	0	0
2019/8/15 17:11	272	3.35	37.2	6.91694	0.148588	950	3.5	1.4	0	0
2019/8/15 17:11	358	3.36	37.2	6.90656	0.148588	950	3.5	1.4	0	0
2019/8/15 17:12	442	3.37	37.1	6.89618	0.148588	950	3.5	1.62	0	0
2019/8/15 17:13	530	3.38	37.2	6.90656	0.148588	950	3.5	1.86	0	0
2019/8/15 17:13	612	3.39	37.2	6.90656	0.148588	950	3.5	2.02	0	0
2019/8/15 17:14	696	3.4	37.1	6.90656	0.148588	950	3.5	2.66	0	0
2019/8/15 17:14	784	3.41	37.2	6.90656	0.148588	950	3.5	2.68	0	0
2019/8/15 17:15	869	3.42	37.1	6.90656	0.148588	950	3.5	2.96	0	0
2019/8/15 17:16	637	3.43	37.1	6.90656	0.148588	950	3.5	3.42	0	0
2019/8/15 17:16	34	3.44	37.2	6.90656	0.148588	950	3.5	3.44	0	0
2019/8/15 17:17	114	3.45	37.1	6.90656	0.148588	950	3.5	3.8	0	0

2019/8/15 17:17	199	3.46	37.2	6.90656	0.148588	950	3.5	3.94	0	0
2019/8/15 17:18	282	3.47	37.2	6.90656	0.148588	950	3.5	4.14	0	0
2019/8/15 17:19	373	3.48	37.1	6.90656	0.148588	950	3.5	4.26	0	0
2019/8/15 17:19	454	3.49	37.1	6.90656	0.148588	950	3.5	4.26	0	0
2019/8/15 17:20	541	3.5	37.3	6.91694	0.148588	950	3.5	4.26	0	0
2019/8/15 17:20	624	3.51	37.1	6.93771	0.148588	950	3.5	4.26	0	0
2019/8/15 17:21	704	3.52	37.1	6.93771	0.148588	950	3.62	4.26	0	0
2019/8/15 17:22	788	3.53	37.2	6.94809	0.148588	950	3.76	4.26	0	0
2019/8/15 17:22	871	3.54	37.3	6.94809	0.148588	950	3.78	4.26	0	0
2019/8/15 17:23	952	3.55	37	6.93771	0.148588	950	3.9	4.26	0	0
2019/8/15 17:23	37	3.56	37.1	6.93771	0.148588	950	4.02	4.26	0	0
2019/8/15 17:24	124	3.57	37.2	6.93771	0.148588	950	4.14	4.26	0	0
2019/8/15 17:25	208	3.58	37.1	6.93771	2.22883	950	4.26	4.26	0	0
2019/8/15 17:25	299	3.59	37	6.94809	6.98366	950	4.4	4.26	0	0
2019/8/15 17:26	386	3.6	37.1	6.94809	8.9153	950	4.4	4.26	0	0
2019/8/15 17:26	332	3.61	37.2	6.93771	7.42942	950	4.5	4.26	0	0
2019/8/15 17:27	419	3.62	37.1	6.93771	5.79495	950	4.62	4.26	0	0
2019/8/15 17:28	645	3.63	37.1	6.93771	5.34918	950	4.62	4.26	0	0
2019/8/15 17:28	725	3.64	37.2	6.93771	4.16048	950	4.74	4.26	0	0
2019/8/15 17:29	805	3.65	37.2	6.93771	3.56612	950	4.84	4.26	0	0
2019/8/15 17:29	680	3.66	37.1	6.93771	3.56612	950	4.96	4.26	0	0
2019/8/15 17:30	774	3.67	37.2	6.93771	3.8633	950	4.96	4.26	0	0
2019/8/15 17:31	57	3.68	37.3	6.93771	3.26894	950	5.08	4.26	0	0
2019/8/15 17:31	144	3.69	37.1	6.94809	3.26894	950	5.2	4.26	0	0
2019/8/15 17:32	236	3.7	37.1	6.93771	3.8633	950	5.2	4.26	0	0
2019/8/15 17:32	317	3.71	37.1	6.93771	4.16048	950	5.3	4.26	0	0
2019/8/15 17:33	395	3.72	37.2	6.93771	3.8633	950	5.42	4.26	0	0
2019/8/15 17:34	480	3.73	37.1	6.93771	3.41753	950	5.42	4.26	0	0
2019/8/15 17:34	563	3.74	37	6.93771	3.8633	950	5.54	4.26	0	0
2019/8/15 17:35	646	3.75	37.2	6.93771	3.56612	950	5.66	4.26	0	0
2019/8/15 17:35	730	3.76	37.2	6.94809	3.56612	950	5.66	4.26	0	0
2019/8/15 17:36	818	3.77	37.1	6.93771	3.56612	950	5.78	4.26	0	0
2019/8/15 17:37	904	3.78	37.1	6.93771	3.8633	950	5.9	4.26	0	0
2019/8/15 17:37	999	3.79	37.2	6.93771	4.16048	950	6.02	4.26	0	0
2019/8/15 17:38	254	3.8	37.3	6.93771	4.16048	950	6.02	4.26	0	0

2019/8/15 17:38	338	3.81	37	6.93771	4.45765	950	6.12	4.26	0	0
2019/8/15 17:39	422	3.82	37	6.93771	5.20059	950	6.24	4.26	0	0
2019/8/15 17:40	355	3.83	37.2	6.94809	5.64636	950	6.24	4.26	0	0
2019/8/15 17:40	439	3.84	37.2	6.93771	6.3893	950	6.33999	4.26	0	0
2019/8/15 17:41	676	3.85	37.1	6.92733	9.06389	950	6.45999	4.26	0	0
2019/8/15 17:41	760	3.86	37	6.93771	11.7385	950	6.45999	4.26	0	0
2019/8/15 17:42	843	3.87	37.2	6.93771	16.0475	950	6.59999	4.26	0	0
2019/8/15 17:43	927	3.88	37.2	6.93771	20.5052	950	6.71999	4.26	0	0
2019/8/15 17:43	16	3.89	37.1	6.93771	26.5973	950	6.71999	4.26	0	0
2019/8/15 17:44	97	3.9	37.1	6.93771	32.3923	950	6.83999	4.26	0	0
2019/8/15 17:44	15	3.91	37.1	6.93771	36.5527	950	6.83999	4.26	0	0
2019/8/15 17:45	100	3.92	37.2	6.93771	39.3759	950	6.95999	4.26	0	0
2019/8/15 17:46	352	3.93	37.2	6.93771	43.9822	950	6.97999	4.26	0.0430556	0.42
2019/8/15 17:46	434	3.94	37.1	6.93771	58.2467	950	7.07999	4.26	0.143056	0.42
2019/8/15 17:47	513	3.95	37	6.93771	70.5795	950	7.19999	4.26	0.243056	0.886667
2019/8/15 17:47	591	3.96	37	6.94809	74.8886	950	7.19999	4.26	0.345833	0.886667
2019/8/15 17:48	740	3.97	37	6.93771	76.8202	950	7.31999	4.26	0.649999	1.35333
2019/8/15 17:49	567	3.98	37.1	6.93771	68.4993	950	7.31999	4.26	0.949999	1.82
2019/8/15 17:49	725	3.99	37.2	6.92733	7.87519	950	7.31999	4.26	1.225	1.82
2019/8/15 17:50	932	4	37.2	6.92733	24.6657	950	7.31999	4.26	1.225	2.28667
2019/8/15 17:50	14	4.01	37	6.92733	57.0579	950	7.31999	4.26	1.225	2.28667
2019/8/15 17:51	31	4.02	37	6.93771	61.6642	950	7.31999	4.26	1.275	2.75333
2019/8/15 17:52	209	4.03	37	6.93771	51.1144	950	7.31999	4.26	1.375	3.22
2019/8/15 17:52	294	4.04	37.1	6.93771	39.9703	950	7.31999	4.26	1.47501	3.22
2019/8/15 17:53	377	4.05	37.2	6.93771	50.9658	950	7.31999	4.26	1.48612	3.64
2019/8/15 17:53	350	4.06	37.2	6.93771	62.8529	950	7.37999	4.26	1.48612	3.64
2019/8/15 17:54	500	4.07	37.2	6.94809	63.893	950	7.53999	4.26	1.48612	4.06
2019/8/15 17:55	586	4.08	37	6.92733	68.2021	950	7.53999	4.26	1.48612	4.48
2019/8/15 17:55	671	4.09	37	6.93771	65.8247	950	7.53999	4.26	1.48612	4.48
2019/8/15 17:56	812	4.1	37	6.93771	65.3789	950	7.53999	4.26	1.48612	4.9
2019/8/15 17:56	903	4.11	37.1	6.93771	65.2303	950	7.57999	4.26	1.48612	4.9
2019/8/15 17:57	987	4.12	37.1	6.92733	63.2987	950	7.71999	4.26	1.48612	5.36666
2019/8/15 17:58	25	4.13	37.2	6.92733	48.4398	950	7.71999	4.26	1.60278	5.83333
2019/8/15 17:58	167	4.14	37.2	6.92733	31.6493	950	7.71999	4.26	1.70418	5.83333
2019/8/15 17:59	253	4.15	37	6.93771	36.107	950	7.71999	4.26	1.80418	6.3

2019/8/15 17:59	341	4.16	37	6.93771	35.364	950	7.71999	4.26	1.90418	6.3
2019/8/15 18:00	424	4.17	37.1	6.93771	34.3239	950	7.71999	4.26	2.00418	6.76666
2019/8/15 18:01	513	4.18	37.2	6.93771	35.9584	950	7.71999	4.26	2.10417	7.23333
2019/8/15 18:01	541	4.19	37.2	6.93771	31.5007	950	7.73999	4.26	2.20416	7.23333
2019/8/15 18:02	626	4.2	37	6.93771	32.2437	950	7.85999	4.26	2.30555	7.65333
2019/8/15 18:02	710	4.21	37.1	6.93771	30.7578	950	7.85999	4.26	2.40554	7.65333
2019/8/15 18:03	847	4.22	37.1	6.93771	26.003	950	7.85999	4.26	2.50553	8.07333
2019/8/15 18:04	929	4.23	37.2	6.93771	26.5973	950	7.85999	4.26	0.0736111	0.14
2019/8/15 18:04	11	4.24	37.1	6.93771	24.5171	950	7.85999	4.26	0.170834	0.606667
2019/8/15 18:05	66	4.25	37	6.93771	26.3001	950	7.93999	4.26	0.272223	0.606667
2019/8/15 18:05	194	4.26	37.1	6.93771	25.1114	950	7.95999	4.26	0.372222	1.10667
2019/8/15 18:06	92	4.27	37.2	6.93771	26.7459	900	8.08	4.26	0.472221	1.60667
2019/8/15 18:07	180	4.28	37.2	6.93771	13.6701	900	8.08	4.26	0.572222	1.60667
2019/8/15 18:07	456	4.29	37	6.93771	12.7786	900	8.08	4.26	0.672223	2.55667
2019/8/15 18:08	535	4.3	37	6.93771	6.3893	900	8.1	4.26	0.0722222	0
2019/8/15 18:08	415	4.31	37.1	6.93771	15.0074	900	8.22	4.26	0.133333	0.5
2019/8/15 18:09	649	4.32	37.2	6.93771	29.4205	900	8.22	4.26	0.183333	0.5
2019/8/15 18:10	796	4.33	37.1	6.93771	26.3001	900	8.22	4.26	0.233333	1
2019/8/15 18:10	882	4.34	37	6.93771	31.7979	900	8.32	4.26	0.283333	1.45
2019/8/15 18:11	964	4.35	37	6.93771	29.4205	900	8.32	4.26	0.333334	1.45
2019/8/15 18:11	45	4.36	37.1	6.93771	30.0149	900	8.34	4.26	0.38264	1.9
2019/8/15 18:12	131	4.37001	37.2	6.93771	32.2437	900	8.48	4.26	0.43264	1.9
2019/8/15 18:13	223	4.38001	37.2	6.93771	26.4487	900	8.48	4.26	0.482641	2.35
2019/8/15 18:13	309	4.39001	37	6.93771	35.2155	900	8.48	4.26	0.532642	2.75
2019/8/15 18:14	151	4.40001	37	6.93771	27.6374	900	8.5	4.26	0.582642	2.8
2019/8/15 18:14	468	4.41001	37.1	6.93771	34.0267	900	8.64001	4.26	0.632643	3.25
2019/8/15 18:15	551	4.42001	37.1	6.93771	28.2318	900	8.64001	4.26	0.683338	3.25
2019/8/15 18:16	636	4.43001	37.2	6.93771	29.1233	900	8.64001	4.26	0.733339	3.7
2019/8/15 18:16	433	4.44001	37	6.93771	34.1753	900	8.64001	4.26	0.78334	4.2
2019/8/15 18:17	805	4.45001	37	6.93771	28.3804	900	8.70001	4.26	0.83334	4.2
2019/8/15 18:17	892	4.46001	37.1	6.93771	35.5126	900	8.82001	4.26	0.883341	4.7
2019/8/15 18:18	975	4.47001	37.1	6.93771	29.5691	900	8.82001	4.26	0.933342	4.7
2019/8/15 18:19	34	4.48001	37.2	6.93771	35.0669	900	8.82001	4.26	0.981953	5.2
2019/8/15 18:19	155	4.49001	37.2	6.93771	37.2957	900	8.82001	4.26	1.03265	5.55
2019/8/15 18:20	248	4.50001	37.1	6.93771	44.5765	900	8.96002	4.26	1.08264	5.7

2019/8/15 18:20	271	4.51001	37	6.93771	59.584	900	8.96002	4.26	1.13264	6.20001
2019/8/15 18:21	428	4.52001	37	6.93771	56.7608	900	8.96002	4.26	1.20014	6.20001
2019/8/15 18:22	514	4.53001	37.1	6.93771	57.5037	900	8.96002	4.26	1.28014	6.65001
2019/8/15 18:22	592	4.54001	37.1	6.93771	59.2868	900	8.96002	4.26	1.36709	7.05001
2019/8/15 18:23	676	4.55001	37.2	6.93771	55.2749	900	8.96002	4.26	1.46709	7.10001
2019/8/15 18:23	757	4.56001	37.2	6.93771	53.4918	900	8.96002	4.26	1.56709	7.55001
2019/8/15 18:24	843	4.57001	37.2	6.93771	52.0059	900	8.96002	4.26	1.66709	7.55001
2019/8/15 18:25	931	4.58001	37	6.93771	54.8291	900	8.96002	4.26	1.76709	8.00001
2019/8/15 18:25	2	4.59001	37	6.93771	53.9376	900	8.96002	4.26	1.88182	8.30001
2019/8/15 18:26	100	4.60001	37.1	6.93771	45.7652	900	8.96002	4.26	2.00182	8.45001
2019/8/15 18:26	184	4.61001	37.1	6.93771	46.8053	900	8.96002	4.26	2.1414	8.95002
2019/8/15 18:27	270	4.62001	37.1	6.93771	36.9985	900	8.96002	4.26	2.2914	8.95002
2019/8/15 18:28	82	4.63001	37.2	6.93771	34.0267	900	8.96002	4.26	2.4414	9.45002
2019/8/15 18:28	448	4.64001	37.1	6.93771	35.9584	900	8.96002	4.26	2.59139	9.85002
2019/8/15 18:29	532	4.65001	37	6.93771	32.9866	900	8.96002	4.26	2.74347	9.95002
2019/8/15 18:29	617	4.66001	37.1	6.92733	32.3923	900	8.96002	4.26	2.89347	10.45
2019/8/15 18:30	702	4.67001	37.1	6.93771	32.0951	900	8.96002	4.26	3.04347	10.45
2019/8/15 18:31	784	4.68001	37.2	6.92733	32.2437	900	8.96002	4.26	3.19347	10.95
2019/8/15 18:31	871	4.69001	37.2	6.92733	29.5691	900	8.96002	4.26	3.34347	11.45
2019/8/15 18:32	962	4.70001	37	6.92733	27.786	900	8.96002	4.26	3.49346	11.45
2019/8/15 18:32	46	4.71001	37	6.92733	33.4324	900	8.96002	4.26	3.64138	11.9
2019/8/15 18:33	134	4.72001	37.1	6.92733	29.8663	900	8.96002	4.26	3.79138	11.9
2019/8/15 18:34	217	4.73001	37.2	6.92733	27.0431	900	8.96002	4.26	3.94137	12.35
2019/8/15 18:34	307	4.74001	37.2	6.92733	29.4205	900	8.96002	4.26	4.09137	12.65
2019/8/15 18:35	164	4.75001	37	6.92733	29.8663	900	8.96002	4.26	4.24137	12.8
2019/8/15 18:35	473	4.76001	37	6.92733	30.1634	900	8.96002	4.26	4.39345	13.25
2019/8/15 18:36	559	4.77001	37.1	6.92733	27.786	900	8.96002	4.26	4.54345	13.25
2019/8/15 18:37	645	4.78001	37.2	6.92733	28.9747	900	8.96002	4.26	4.69345	13.7
2019/8/15 18:37	729	4.79001	37.2	6.92733	29.5691	900	8.96002	4.26	4.84344	14.1
2019/8/15 18:38	816	4.80001	37.1	6.92733	25.1114	900	8.96002	4.26	4.99344	14.2
2019/8/15 18:38	898	4.81002	37	6.92733	29.1233	900	8.96002	4.26	5.14344	14.7
2019/8/15 18:39	983	4.82002	37	6.92733	28.3804	900	8.96002	4.26	5.29552	14.7
2019/8/15 18:40	64	4.83002	37.1	6.92733	24.6657	900	8.96002	4.26	5.44135	15.2
2019/8/15 18:40	152	4.84002	37.2	6.92733	27.1917	900	8.96002	4.26	5.59135	15.45
2019/8/15 18:41	235	4.85002	37.1	6.92733	27.9346	900	8.96002	4.26	5.74135	15.7

2019/8/15 18:41	321	4.86002	37	6.92733	29.7177	900	8.96002	4.26	5.89134	16.2
2019/8/15 18:42	404	4.87002	37	6.91694	25.5572	900	8.96002	4.26	6.04134	16.2
2019/8/15 18:43	492	4.88002	37.1	6.91694	27.1917	900	8.96002	4.26	6.19134	16.7
2019/8/15 18:43	577	4.89002	37.2	6.91694	29.1233	900	8.96002	4.26	6.34342	17.05
2019/8/15 18:44	661	4.90002	37.2	6.91694	26.3001	900	8.96002	4.26	6.49342	17.15
2019/8/15 18:44	746	4.91002	37	6.91694	27.3403	900	8.96002	4.26	6.64342	17.6
2019/8/15 18:45	834	4.92002	37.1	6.91694	27.4889	900	8.96002	4.26	6.79341	17.6
2019/8/15 18:46	919	4.93002	37.1	6.91694	26.003	900	8.96002	4.26	6.94341	18.05
2019/8/15 18:46	3	4.94002	37.2	6.91694	25.7058	900	8.96002	4.26	7.08924	18.25
2019/8/15 18:47	90	4.95002	37.2	6.91694	25.8544	900	8.96002	4.26	7.24132	18.5
2019/8/15 18:47	179	4.96002	37	6.91694	30.6092	900	8.96002	4.26	7.39132	18.95
2019/8/15 18:48	259	4.97002	37	6.91694	25.26	900	8.96002	4.26	7.54132	18.95
2019/8/15 18:49	342	4.98002	37.1	6.91694	24.9629	900	8.96002	4.26	7.69132	19.45
2019/8/15 18:49	427	4.99002	37.2	6.91694	28.8262	900	8.96002	4.26	7.84131	19.75
2019/8/15 18:50	510	5.00002	37.2	6.91694	25.7058	900	8.96002	4.26	7.99339	19.95
2019/8/15 18:50	599	5.01002	37	6.91694	27.3403	900	8.96002	4.26	8.14342	20.45
2019/8/15 18:51	684	5.02002	37.1	6.90656	28.529	900	8.96002	4.26	8.29346	20.45
2019/8/15 18:52	763	5.03002	37.2	6.91694	29.4205	900	8.96002	4.26	8.44349	20.95
2019/8/15 18:52	850	5.04002	37.2	6.91694	26.8945	900	8.96002	4.26	8.59352	21.35
2019/8/15 18:53	941	5.05002	37	6.91694	25.5572	900	8.96002	4.26	8.74564	21.45
2019/8/15 18:53	22	5.06002	37	6.90656	32.9866	900	8.96002	4.26	8.8915	21.95
2019/8/15 18:54	109	5.07002	37	6.91694	31.3522	900	8.96002	4.26	9.04153	21.95
2019/8/15 18:55	196	5.08002	37.1	6.91694	27.9346	900	8.96002	4.26	9.19157	22.3999
2019/8/15 18:55	284	5.09002	37.2	6.90656	30.6092	900	8.96002	4.26	9.3416	22.5999
2019/8/15 18:56	367	5.10002	37.2	6.91694	30.6092	900	8.96002	4.26	9.49163	22.8499
2019/8/15 18:56	451	5.11002	36.9	6.90656	33.581	900	8.96002	4.26	9.64375	23.2999
2019/8/15 18:57	538	5.12002	36.9	6.90656	31.5007	900	8.96002	4.26	9.79378	23.2999
2019/8/15 18:58	623	5.13002	37	6.90656	32.838	900	8.96002	4.26	9.94381	23.7499
2019/8/15 18:58	707	5.14002	37.1	6.90656	34.6211	900	8.96002	4.26	10.0938	24.0999
2019/8/15 18:59	791	5.15002	37.2	6.90656	30.0149	900	8.96002	4.26	10.2439	24.1999
2019/8/15 18:59	877	5.16002	37.2	6.90656	33.4324	900	8.96002	4.26	10.3939	24.6999
2019/8/15 19:00	959	5.17002	37	6.90656	34.4725	900	8.96002	4.26	10.546	24.6999
2019/8/15 19:01	42	5.18002	37	6.90656	32.2437	900	8.96002	4.26	10.6919	25.1999
2019/8/15 19:01	0	5.19002	37.1	6.90656	34.4725	900	8.96002	4.26	10.8419	25.3999
2019/8/15 19:02	219	5.20002	37.1	6.90656	34.1753	900	8.96002	4.26	10.992	25.6999

2019/8/15 19:02	302	5.21002	37.2	6.90656	36.4042	900	8.96002	4.32	11.142	26.1999
2019/8/15 19:03	392	5.22002	37	6.90656	32.2437	900	8.96002	4.34	11.2941	26.1999
2019/8/15 19:04	476	5.23002	37	6.90656	34.0267	900	8.96002	4.38	11.4441	26.6999
2019/8/15 19:04	561	5.24003	37	6.90656	36.8499	900	8.96002	4.64	11.5942	26.9999
2019/8/15 19:05	647	5.25003	37.1	6.90656	31.7979	900	8.96002	4.64	11.7442	27.1999
2019/8/15 19:05	727	5.26003	37.2	6.90656	35.0669	900	8.96002	4.64	11.8942	27.6499
2019/8/15 19:06	813	5.27003	37.2	6.90656	35.9584	900	8.96002	4.64	12.0443	27.6499
2019/8/15 19:07	897	5.28003	37	6.90656	36.4042	900	8.96002	4.64	12.1943	28.0999
2019/8/15 19:07	983	5.29003	37	6.90656	35.9584	900	8.96002	4.64	12.3464	28.4499
2019/8/15 19:08	65	5.30003	37.1	6.90656	34.9183	900	8.96002	4.66	12.4923	28.5499
2019/8/15 19:08	147	5.31003	37.1	6.90656	38.3358	900	8.96002	4.68	12.6423	28.9998
2019/8/15 19:09	225	5.32003	37.2	6.90656	33.7296	900	8.96002	4.86	12.7923	28.9998
2019/8/15 19:10	310	5.33003	37.2	6.90656	32.5409	900	8.96002	5.66	12.9424	29.4498
2019/8/15 19:10	397	5.34003	37	6.90656	36.9985	900	8.96002	5.84	13.0945	29.6498
2019/8/15 19:11	481	5.35003	37	6.90656	34.3239	900	8.96002	5.84	13.2445	29.9498
2019/8/15 19:11	568	5.36003	37.1	6.90656	34.7697	900	8.96002	5.84	13.3945	30.4498
2019/8/15 19:12	657	5.37003	37.1	6.90656	34.7697	900	8.96002	5.86	13.5446	30.4498
2019/8/15 19:13	742	5.38003	37.2	6.90656	34.7697	900	8.96002	5.86	13.6946	30.9498
2019/8/15 19:13	821	5.39003	37.2	6.90656	33.8782	900	8.96002	5.9	13.8446	31.2498
2019/8/15 19:14	907	5.40003	37	6.90656	32.6894	900	8.96002	5.92	13.9947	31.4498
2019/8/15 19:14	988	5.41003	37	6.90656	38.1872	900	8.96002	5.92	14.1468	31.9498
2019/8/15 19:15	0	5.42003	37.1	6.90656	34.6211	900	8.96002	5.98	14.2927	31.9498
2019/8/15 19:16	0	5.43003	37.2	6.90656	31.6493	900	8.96002	5.98	14.4427	32.3998
2019/8/15 19:16	245	5.44003	37.2	6.90656	35.364	900	8.96002	5.98	14.5927	32.4998
2019/8/15 19:17	322	5.45003	37	6.90656	34.9183	900	8.96002	6	14.7428	32.8498
2019/8/15 19:17	405	5.46003	37	6.90656	36.4042	900	8.96002	6	14.8949	33.2998
2019/8/15 19:18	493	5.47003	37	6.90656	32.9866	900	8.96002	6.04	15.0449	33.2998
2019/8/15 19:19	577	5.48003	37.1	6.90656	34.3239	900	8.96002	6.1	15.1949	33.7498
2019/8/15 19:19	662	5.49003	37.2	6.90656	34.9183	900	8.96002	6.1	15.345	33.9498
2019/8/15 19:20	749	5.50003	37.1	6.90656	30.6092	900	8.96002	6.1	15.495	34.1998
2019/8/15 19:20	831	5.51003	37	6.90656	36.107	900	8.96002	6.1	15.645	34.6498
2019/8/15 19:21	915	5.52003	37	6.90656	36.5527	900	8.96002	6.18	15.7971	34.6498
2019/8/15 19:22	0	5.53003	37.1	6.90656	33.8782	900	8.96002	6.18	15.9472	35.1497
2019/8/15 19:22	87	5.54003	37.2	6.91694	34.6211	900	8.96002	6.41999	16.093	35.2497
2019/8/15 19:23	169	5.55003	37.2	6.91694	32.6894	900	8.96002	6.41999	16.243	35.6497

2019/8/15 19:23	250	5.56003	37	6.90656	34.4725	900	8.96002	6.41999	16.3929	36.1497
2019/8/15 19:24	333	5.57003	37	6.90656	32.0951	900	8.96002	6.41999	16.5429	36.1497
2019/8/15 19:25	415	5.58003	37.1	6.90656	34.6211	900	8.96002	6.41999	16.6949	36.6497
2019/8/15 19:25	502	5.59003	37.1	6.90656	35.9584	900	8.96002	6.41999	16.8449	36.8497
2019/8/15 19:26	591	5.60003	37.2	6.90656	32.2437	900	8.96002	6.41999	16.9949	37.1497
2019/8/15 19:26	675	5.61003	37.2	6.90656	33.581	900	8.96002	6.41999	17.1448	37.5997
2019/8/15 19:27	757	5.62003	37	6.90656	33.4324	900	8.96002	6.41999	17.2948	37.5997
2019/8/15 19:28	840	5.63003	37	6.90656	33.1352	900	8.96002	6.41999	17.4468	38.0497
2019/8/15 19:28	919	5.64003	37	6.90656	33.2838	900	8.96002	6.41999	17.5968	38.2997
2019/8/15 19:29	3	5.65003	37.1	6.90656	32.3923	900	8.96002	6.41999	17.7468	38.4997
2019/8/15 19:29	86	5.66003	37.2	6.90656	37.1471	900	8.96002	6.41999	17.8967	38.9497
2019/8/15 19:30	165	5.67003	37.2	6.90656	33.2838	900	8.96002	6.45999	18.0467	38.9497
2019/8/15 19:31	248	5.68004	37	6.90656	32.5409	900	8.96002	6.45999	18.1966	39.3997
2019/8/15 19:31	332	5.69004	37	6.90656	37.2957	900	8.96002	6.45999	18.3466	39.7497
2019/8/15 19:32	414	5.70004	37.1	6.90656	35.2155	900	8.96002	6.45999	18.4987	39.8497
2019/8/15 19:32	497	5.71004	37.1	6.90656	35.364	900	8.96002	6.45999	18.6486	40.3497
2019/8/15 19:33	581	5.72004	37.2	6.90656	32.3923	900	8.96002	6.45999	18.7986	40.3497
2019/8/15 19:34	667	5.73004	37.1	6.90656	33.2838	900	8.96002	6.47999	18.9485	40.8497
2019/8/15 19:34	751	5.74004	37	6.90656	36.8499	900	8.96002	6.49999	19.0985	41.2997
2019/8/15 19:35	841	5.75004	37.1	6.90656	31.5007	900	8.96002	6.59999	19.2485	41.3497
2019/8/15 19:35	923	5.76004	37.1	6.90656	35.6612	900	8.96002	6.61999	19.4005	41.8496
2019/8/15 19:36	6	5.77004	37.2	6.90656	33.581	900	8.96002	6.67999	19.5463	41.8496
2019/8/15 19:37	85	5.78004	37	6.90656	30.9064	900	8.96002	6.67999	19.6963	42.3496
2019/8/15 19:37	168	5.79004	37	6.90656	39.0788	900	8.96002	6.67999	19.8462	42.6996
2019/8/15 19:38	253	5.80004	37	6.90656	38.3358	900	8.96002	6.67999	19.9962	42.8496
2019/8/15 19:38	335	5.81004	37.1	6.90656	41.3076	900	8.96002	6.67999	20.1462	43.3496
2019/8/15 19:39	417	5.82004	37.2	6.90656	37.5929	900	8.96002	6.71999	20.2982	43.3496
2019/8/15 19:40	500	5.83004	37.2	6.90656	39.9703	900	8.96002	6.71999	20.4482	43.7996
2019/8/15 19:40	590	5.84004	37	6.90656	43.0906	900	8.96002	6.71999	20.5981	44.1496
2019/8/15 19:41	682	5.85004	37	6.90656	39.2273	900	8.96002	6.73999	20.7481	44.2496
2019/8/15 19:41	766	5.86004	37	6.90656	43.0906	900	8.96002	6.73999	20.8981	44.6996
2019/8/15 19:42	854	5.87004	37.1	6.90656	43.0906	900	8.96002	6.73999	21.0501	44.6996
2019/8/15 19:43	935	5.88004	37.2	6.90656	41.4562	900	8.96002	6.77999	21.2001	45.1496
2019/8/15 19:43	18	5.89004	37.2	6.90656	41.9019	900	8.96002	6.79999	21.3459	45.3996
2019/8/15 19:44	100	5.90004	37	6.90656	40.7132	900	8.96002	6.81999	21.4958	45.5996

2019/8/15 19:44	192	5.91004	36.9	6.90656	45.4681	900	8.96002	6.81999	21.6458	46.0996
2019/8/15 19:45	277	5.92004	37	6.90656	42.6449	900	8.96002	6.81999	21.7958	46.0996
2019/8/15 19:46	369	5.93004	37	6.90656	42.942	900	8.96002	6.81999	21.9587	46.5996
2019/8/15 19:46	454	5.94004	37.1	6.90656	36.5527	900	8.96002	6.83999	22.1387	46.9496
2019/8/15 19:47	538	5.95004	37.2	6.90656	31.055	900	8.96002	6.97999	22.3188	47.0996
2019/8/15 19:47	625	5.96004	37	6.90656	34.3239	900	8.96002	6.97999	22.4988	47.5996
2019/8/15 19:48	706	5.97004	37	6.90656	32.6894	900	8.96002	6.97999	22.6788	47.5996
2019/8/15 19:49	789	5.98004	37.1	6.90656	32.5409	900	8.96002	6.97999	22.8614	48.0996
2019/8/15 19:49	880	5.99004	37.1	6.90656	35.8098	900	8.96002	7.03999	23.0414	48.4995
2019/8/15 19:50	968	6.00004	37.2	6.90656	33.581	900	8.96002	7.11999	23.2214	48.5495
2019/8/15 19:50	51	6.01004	37	6.90656	35.0669	900	8.96002	7.11999	23.3965	48.9995
2019/8/15 19:51	131	6.02004	36.9	6.90656	35.8098	900	8.96002	7.11999	23.5765	48.9995
2019/8/15 19:52	214	6.03004	36.9	6.90656	34.3239	900	8.96002	7.13999	23.7566	49.4495
2019/8/15 19:52	302	6.04004	37.1	6.90656	36.8499	900	8.96002	7.15999	23.9366	49.6995
2019/8/15 19:53	389	6.05004	37.2	6.90656	34.1753	900	8.96002	7.23999	24.1191	49.8995
2019/8/15 19:53	470	6.06004	37.2	6.90656	36.2556	900	8.96002	7.25999	24.2992	50.3495
2019/8/15 19:54	558	6.07004	37	6.90656	35.364	900	8.96002	7.25999	24.4792	50.3495
2019/8/15 19:55	640	6.08004	37	6.90656	35.0669	900	8.96002	7.59999	24.6593	50.8495
2019/8/15 19:55	723	6.09004	37	6.90656	36.2556	900	8.96002	7.59999	24.8393	51.1995
2019/8/15 19:56	804	6.10004	37.1	6.90656	34.1753	900	8.96002	7.59999	25.0218	51.3495
2019/8/15 19:56	889	6.11004	37.2	6.90656	35.2155	900	8.96002	7.59999	25.2019	51.8495
2019/8/15 19:57	973	6.12005	37	6.90656	34.0267	900	8.96002	7.63999	25.3819	51.8495
2019/8/15 19:58	51	6.13005	37	6.90656	34.0267	900	8.96002	7.63999	25.5569	52.3495
2019/8/15 19:58	134	6.14005	37.1	6.90656	35.6612	900	8.96002	7.63999	25.737	52.5995
2019/8/15 19:59	220	6.15005	37.2	6.90656	32.5409	900	8.96002	7.65999	25.917	52.8495
2019/8/15 19:59	305	6.16005	37.2	6.90656	36.8499	900	8.96002	7.75999	26.0971	53.3495
2019/8/15 20:00	385	6.17005	37	6.90656	35.2155	900	8.96002	7.75999	26.2796	53.3495
2019/8/15 20:01	469	6.18005	37	6.90656	34.9183	900	8.96002	7.75999	26.4596	53.7995
2019/8/15 20:01	551	6.19005	37.1	6.90656	37.5929	900	8.96002	7.75999	26.6397	54.0495
2019/8/15 20:02	639	6.20005	37.2	6.90656	35.5126	900	8.96002	7.75999	26.8197	54.2495
2019/8/15 20:02	723	6.21005	37.2	6.90656	37.5929	900	8.96002	7.75999	26.9998	54.6995
2019/8/15 20:03	804	6.22005	36.9	6.90656	37.1471	900	8.96002	7.75999	27.1823	54.6995
2019/8/15 20:04	888	6.23005	37	6.90656	36.9985	900	8.96002	7.75999	27.3623	55.1494
2019/8/15 20:04	970	6.24005	37	6.90656	38.7816	900	8.96002	7.79999	27.5424	55.5494
2019/8/15 20:05	56	6.25005	37.1	6.90656	36.7013	900	8.96002	7.81999	27.7174	55.5994

2019/8/15 20:05	141	6.26005	37.2	6.90656	37.7415	900	8.96002	7.83999	27.8974	56.0994
2019/8/15 20:06	230	6.27005	37	6.90656	35.5126	900	8.96002	7.97999	28.08	56.0994
2019/8/15 20:07	322	6.28005	36.9	6.90656	36.107	900	8.96002	7.97999	28.26	56.5994
2019/8/15 20:07	406	6.29005	37	6.90656	36.7013	900	8.96002	7.97999	28.4401	56.8494
2019/8/15 20:08	491	6.30005	37.1	6.90656	34.3239	900	8.96002	7.97999	28.6201	57.0994
2019/8/15 20:08	573	6.31005	37.2	6.90656	37.1471	900	8.96002	7.97999	28.8001	57.5994
2019/8/15 20:09	658	6.32005	37.2	6.90656	36.4042	900	8.96002	7.97999	28.9802	57.5994
2019/8/15 20:10	744	6.33005	36.9	6.90656	35.6612	900	8.96002	7.97999	29.1627	58.0994
2019/8/15 20:10	827	6.34005	36.9	6.90656	38.1872	900	8.96002	7.97999	29.3428	58.3994
2019/8/15 20:11	912	6.35005	37.1	6.90656	36.2556	900	8.96002	8.01999	29.5228	58.5494
2019/8/15 20:11	995	6.36005	37.1	6.90656	38.4844	900	8.96002	8.05999	29.7028	58.9994
2019/8/15 20:12	78	6.37005	37.2	6.90656	37.89	900	8.96002	8.05999	29.8779	58.9994
2019/8/15 20:13	160	6.38005	37	6.90656	36.9985	900	8.96002	8.08	30.0579	59.4494
2019/8/15 20:13	246	6.39005	36.9	6.90656	38.9302	900	8.96002	8.1	30.2404	59.5994
2019/8/15 20:14	333	6.40005	37	6.90656	36.8499	900	8.96002	8.18	30.4205	59.8994
2019/8/15 20:14	417	6.41005	37.1	6.90656	39.6731	900	8.96002	8.18	30.6005	60.3494
2019/8/15 20:15	503	6.42005	37.2	6.90656	37.1471	900	8.96002	8.18	30.7806	60.3494
2019/8/15 20:16	585	6.43005	37.1	6.90656	36.8499	900	8.96002	8.18	30.9606	60.7994
2019/8/15 20:16	668	6.44005	36.9	6.90656	39.2273	900	8.96002	8.2	31.1406	61.0494
2019/8/15 20:17	753	6.45005	37	6.90656	36.5527	900	8.96002	8.2	31.3232	61.2994
2019/8/15 20:17	836	6.46005	37	6.90656	39.5245	900	8.96002	8.22	31.5032	61.7993
2019/8/15 20:18	921	6.47005	37.1	6.90656	36.9985	900	8.96002	8.22	31.6833	61.7993
2019/8/15 20:19	0	6.48005	37.2	6.90656	36.5527	900	8.96002	8.22	31.8583	62.2993
2019/8/15 20:19	87	6.49005	37	6.90656	38.1872	900	8.96002	8.28	32.0383	62.3993
2019/8/15 20:20	167	6.50005	36.9	6.90656	36.5527	900	8.96002	8.28	32.2182	62.7993
2019/8/15 20:20	250	6.51005	37	6.90656	39.5245	900	8.96002	8.28	32.4006	63.2993
2019/8/15 20:21	335	6.52005	37.1	6.90656	38.7816	900	8.96002	8.32	32.5805	63.2993
2019/8/15 20:22	418	6.53005	37.2	6.90656	37.4443	900	8.96002	8.38	32.7604	63.7493
2019/8/15 20:22	504	6.54005	37.1	6.90656	38.7816	900	8.96002	8.38	32.9403	63.9493
2019/8/15 20:23	592	6.55006	36.9	6.90656	38.7816	900	8.96002	8.38	33.1202	64.1993
2019/8/15 20:23	678	6.56006	37	6.90656	41.159	900	8.96002	8.38	33.3026	64.6493
2019/8/15 20:24	762	6.57006	37.1	6.90656	38.7816	900	8.96002	8.44	33.4825	64.6493
2019/8/15 20:25	846	6.58006	37.2	6.90656	39.6731	900	8.96002	8.44	33.6624	65.0994
2019/8/15 20:25	933	6.59006	37.1	6.90656	40.416	900	8.96002	8.44	33.8423	65.3994
2019/8/15 20:26	19	6.60006	37	6.90656	39.3759	900	8.96002	8.44	34.0172	65.5494

2019/8/15 20:26	100	6.61006	37	6.90656	42.0505	900	8.96002	8.46	34.1971	65.9994
2019/8/15 20:27	190	6.62006	37.1	6.90656	39.2273	900	8.96002	8.46	34.377	65.9994
2019/8/15 20:28	271	6.63006	37.2	6.90656	38.1872	900	8.96002	8.48	34.5594	66.4995
2019/8/15 20:28	361	6.64006	36.9	6.90656	38.9302	900	8.96002	8.48	34.7393	66.6495
2019/8/15 20:29	442	6.65006	36.9	6.90656	37.5929	900	8.96002	8.5	34.9192	66.9995
2019/8/15 20:29	529	6.66006	37	6.90656	40.8618	900	8.96002	8.56001	35.0991	67.4995
2019/8/15 20:30	612	6.67006	37.1	6.90656	38.1872	900	8.96002	8.56001	35.279	67.4995
2019/8/15 20:31	697	6.68006	37.2	6.90656	37.7415	900	8.96002	8.60001	35.4589	67.9995
2019/8/15 20:31	779	6.69006	37.2	6.90656	39.0788	900	8.96002	8.60001	35.6413	68.2496
2019/8/15 20:32	865	6.70006	37	6.90656	38.3358	900	8.96002	8.60001	35.8212	68.4996
2019/8/15 20:32	955	6.71006	36.9	6.90656	41.9019	900	8.96002	8.64001	36.0011	68.9996
2019/8/15 20:33	379	6.72006	37	6.90656	41.6048	900	8.96002	8.72001	36.1785	68.9996
2019/8/15 20:34	468	6.73006	37.1	6.90656	41.0104	900	8.96002	8.72001	36.3584	69.4996
2019/8/15 20:34	371	6.74006	37.2	6.90656	42.3477	900	8.96002	8.72001	36.5383	69.6497
2019/8/15 20:35	456	6.75006	37.1	6.90656	39.8217	900	8.96002	8.78001	36.7207	69.9497
2019/8/15 20:35	723	6.76006	37	6.90656	43.2392	900	8.96002	8.78001	36.9006	70.3997
2019/8/15 20:36	810	6.77006	37	6.90656	40.1189	900	8.96002	9.20002	37.0805	70.3997
2019/8/15 20:37	893	6.78006	37.1	6.90656	39.2273	900	8.96002	9.20002	37.2604	70.8497
2019/8/15 20:37	977	6.79006	37.2	6.90656	40.416	900	8.96002	9.20002	37.4403	71.0997
2019/8/15 20:38	58	6.80006	37.1	6.90656	36.9985	900	8.96002	9.20002	37.6152	71.2998
2019/8/15 20:38	147	6.81006	37	6.90656	41.4562	900	8.96002	9.20002	37.7976	71.7498
2019/8/15 20:39	229	6.82006	37	6.90656	38.7816	900	8.96002	9.24002	37.9775	71.7498
2019/8/15 20:40	308	6.83006	37.1	6.90656	38.3358	900	8.96002	9.24002	38.1574	72.2498
2019/8/15 20:40	394	6.84006	37.2	6.90656	40.2675	900	8.96002	9.24002	38.3373	72.3498
2019/8/15 20:41	477	6.85006	37.3	6.90656	38.0386	900	8.96002	9.30002	38.5172	72.7498
2019/8/15 20:41	563	6.86006	36.9	6.90656	41.6048	900	8.96002	9.30002	38.6971	73.2499
2019/8/15 20:42	647	6.87006	36.9	6.90656	40.8618	900	8.96002	9.30002	38.877	73.2499
2019/8/15 20:43	733	6.88006	37	6.90656	39.9703	900	8.96002	9.40003	39.0594	73.7499
2019/8/15 20:43	816	6.89006	37.1	6.90656	41.6048	900	8.96002	9.40003	39.2393	73.9499
2019/8/15 20:44	904	6.90006	37.2	6.90656	41.0104	900	8.96002	9.42003	39.4192	74.2499
2019/8/15 20:44	987	6.91006	37.2	6.90656	42.7935	900	8.96002	9.44003	39.5991	74.75
2019/8/15 20:45	70	6.92006	37	6.90656	40.5646	900	8.96002	9.50003	39.774	74.75
2019/8/15 20:46	153	6.93006	37	6.90656	40.7132	900	8.96002	9.54003	39.9564	75.25
2019/8/15 20:46	244	6.94006	37.1	6.90656	42.1991	900	8.96002	9.64003	40.1363	75.25
2019/8/15 20:47	327	6.95006	37.2	6.90656	39.5245	900	8.96002	9.72003	40.3162	75.7

2019/8/15 20:47	409	6.96006	37.1	6.90656	41.6048	900	8.96002	9.72003	40.4962	76.15
2019/8/15 20:48	495	6.97006	36.9	6.90656	40.8618	900	8.96002	9.72003	40.6761	76.15
2019/8/15 20:49	578	6.98006	37	6.90656	40.8618	900	8.96002	9.94004	40.856	76.6001
2019/8/15 20:49	666	6.99007	37.1	6.90656	41.6048	900	8.96002	9.94004	41.0359	76.7501
2019/8/15 20:50	757	7.00007	37.1	6.90656	38.7816	900	8.96002	9.94004	41.2183	77.0501
2019/8/15 20:50	838	7.01007	37.2	6.90656	40.1189	900	8.96002	9.94004	41.3982	77.5001
2019/8/15 20:51	921	7.02007	36.9	6.90656	39.6731	900	8.96002	9.94004	41.5781	77.5001
2019/8/15 20:52	5	7.03007	36.9	6.90656	39.2273	900	8.96002	9.96004	41.753	78.0002
2019/8/15 20:52	86	7.04007	37	6.90656	40.416	900	8.96002	10	41.9329	78.0002
2019/8/15 20:53	172	7.05007	37.1	6.90656	38.9302	900	8.96002	10.1	42.1153	78.5002
2019/8/15 20:53	253	7.06007	37.2	6.90656	41.4562	900	8.96002	10.12	42.2952	79.0002
2019/8/15 20:54	336	7.07007	37	6.90656	40.416	900	8.96002	10.2	42.4751	79.0002
2019/8/15 20:55	417	7.08007	37	6.90656	40.7132	900	8.96002	10.24	42.655	79.5003
2019/8/15 20:55	505	7.09007	37	6.90656	41.7533	900	8.96002	10.26	42.8349	79.6003
2019/8/15 20:56	591	7.10007	37.2	6.90656	39.9703	900	8.96002	10.28	43.0148	80.0003
2019/8/15 20:56	674	7.11007	37.2	6.90656	42.0505	900	8.96002	10.28	43.1972	80.5003
2019/8/15 20:57	756	7.12007	37	6.90656	40.5646	900	8.96002	10.32	43.3771	80.5003
2019/8/15 20:58	842	7.13007	36.9	6.90656	40.8618	900	8.96002	10.36	43.557	80.9503
2019/8/15 20:58	926	7.14007	37	6.90656	41.6048	900	8.96002	10.38	43.7369	81.1003
2019/8/15 20:59	8	7.15007	37.1	6.90656	39.0788	900	8.96002	10.38	43.9118	81.4004
2019/8/15 20:59	93	7.16007	37.2	6.90656	40.5646	900	8.96002	10.46	44.0917	81.8504
2019/8/15 21:00	180	7.17007	37	6.90656	39.3759	900	8.96002	10.46	44.2741	81.8504
2019/8/15 21:01	265	7.18007	37	6.90656	39.2273	900	8.96002	10.46	44.454	82.3004
2019/8/15 21:01	352	7.19007	37	6.90656	40.5646	900	8.96002	10.5001	44.6339	82.3004
2019/8/15 21:02	441	7.20007	37.1	6.90656	38.7816	900	8.96002	10.5001	44.8138	82.7505
2019/8/15 21:02	526	7.21007	37.2	6.90656	40.5646	900	8.96002	10.6001	44.9937	83.2505
2019/8/15 21:03	625	7.22007	37.1	6.90656	39.0788	900	8.96002	10.6001	45.1761	83.2505
2019/8/15 21:04	715	7.23007	36.9	6.90656	39.9703	900	8.96002	10.6001	45.356	83.7505
2019/8/15 21:04	807	7.24007	37	6.90656	42.3477	900	8.96002	10.6201	45.5359	83.9005
2019/8/15 21:05	897	7.25007	37	6.90656	41.159	900	8.96002	10.6601	45.7158	84.2505
2019/8/15 21:05	990	7.26007	37.1	6.90656	43.5364	900	8.96002	10.7001	45.8957	84.7506
2019/8/15 21:06	71	7.27007	37.2	6.90656	40.8618	900	8.96002	10.7001	46.0731	84.7506
2019/8/15 21:07	158	7.28007	37	6.90656	40.8618	900	8.96002	10.7001	46.2563	85.2506
2019/8/15 21:07	0	7.29007	37	6.90656	36.8499	900	8.96002	10.7401	46.4813	85.2506
2019/8/15 21:08	329	7.30007	37.1	6.90656	19.3165	900	8.96002	10.9001	46.7312	85.7506

2019/8/15 21:08	416	7.31007	37.2	6.90656	16.3447	900	8.96002	10.9401	46.9812	86.2507
2019/8/15 21:09	305	7.32007	37	6.90656	16.4933	900	8.96002	10.9801	47.2311	86.2507
2019/8/15 21:10	454	7.33007	36.9	6.90656	19.1679	900	8.96002	10.9801	47.481	86.7007
2019/8/15 21:10	693	7.34007	37	6.90656	21.2481	900	8.96002	11.0801	47.7344	86.7507
2019/8/15 21:11	784	7.35007	37.2	6.90656	18.425	900	8.96002	11.0801	47.9844	87.1507
2019/8/15 21:11	872	7.36007	37.2	6.90656	19.0193	900	8.96002	11.2001	48.2343	89.8009
2019/8/15 21:12	955	7.37007	37	6.90656	0.297177	900	8.96002	11.5201	48.4843	97.4013
2019/8/15 21:13	39	7.38007	37	6.90656	0	900	8.96002	12.1801	48.7273	104.902
2019/8/15 21:13	127	7.39007	37.2	6.90656	0	900	8.96002	12.7201	48.9751	112.502
2019/8/15 21:14	162	7.40007	37	6.90656	0	900	8.96002	12.7801	49.0418	113.602
2019/8/15 21:14	305	7.41007	37	6.91694	0	900	8.96002	12.7801	49.0418	113.602
2019/8/15 21:15	401	7.42007	37	6.92733	0	900	8.96002	12.7801	49.0418	113.602
2019/8/15 21:16	501	7.43008	37.2	6.93771	0	900	8.96002	12.7801	49.0418	113.602
2019/8/15 21:16	591	7.44008	37	6.93771	0	900	9.06002	12.7801	49.0418	113.602
2019/8/15 21:17	587	7.45008	36.9	6.93771	0	900	9.16002	12.7801	49.0418	113.602
2019/8/15 21:17	674	7.46008	37	6.93771	23.0312	900	9.34002	12.7801	49.0973	113.602
2019/8/15 21:18	759	7.47008	37.1	6.93771	20.3566	900	9.34002	12.7801	49.2973	113.602
2019/8/15 21:19	945	7.48008	37.2	6.93771	14.8588	900	9.48003	12.7801	49.4911	113.602
2019/8/15 21:19	29	7.49008	37	6.93771	17.682	900	9.48003	12.7801	49.666	113.602
2019/8/15 21:20	0	7.50008	36.9	6.93771	20.951	900	9.52003	12.7801	49.8484	113.602
2019/8/15 21:20	87	7.51008	37	6.93771	20.0594	900	9.56003	12.7801	50.0283	113.602
2019/8/15 21:21	292	7.52008	37.1	6.93771	17.3848	900	9.72003	12.7801	50.2082	113.602
2019/8/15 21:22	376	7.53008	37.2	6.93771	19.7623	900	9.72003	12.7801	50.3881	113.602
2019/8/15 21:22	459	7.54008	37	6.93771	37.5929	900	9.72003	12.7801	50.568	113.602
2019/8/15 21:23	542	7.55008	36.9	6.93771	45.9138	900	9.72003	12.7801	50.7504	113.602
2019/8/15 21:23	857	7.56008	37	6.93771	45.7652	900	9.72003	12.7801	50.9464	113.602
2019/8/15 21:24	669	7.57008	37.1	6.93771	43.8336	900	9.72003	12.7801	51.1464	113.602
2019/8/15 21:25	24	7.58008	37.1	6.93771	36.9985	900	9.72003	12.7801	51.3408	114.702
2019/8/15 21:25	105	7.59008	37.2	6.92733	28.9747	900	9.72003	12.7801	51.5435	115.352
2019/8/15 21:26	189	7.60008	36.9	6.92733	30.312	900	9.72003	12.7801	51.7435	116.152
2019/8/15 21:26	277	7.61008	36.9	6.92733	31.6493	900	9.72003	12.7801	51.9434	116.903
2019/8/15 21:27	361	7.62008	37	6.92733	29.2719	900	9.72003	12.7801	52.1434	117.553
2019/8/15 21:28	397	7.63008	37.1	6.92733	27.786	900	9.72003	12.7801	52.3433	118.203
2019/8/15 21:28	480	7.64008	37.2	6.92733	26.3001	900	9.72003	12.7801	52.5433	118.903
2019/8/15 21:29	328	7.65008	36.9	6.92733	24.3685	900	9.72003	12.7801	52.746	119.603

2019/8/15 21:29	697	7.66008	36.9	6.92733	22.734	900	9.72003	12.7801	52.9459	120.303
2019/8/15 21:30	784	7.67008	37	6.91694	23.477	900	9.72003	12.7801	53.1459	121.003
2019/8/15 21:31	680	7.68008	37.2	6.91694	23.1798	900	9.72003	12.7801	53.3459	121.703
2019/8/15 21:31	658	7.69008	37.2	6.91694	20.8024	900	9.72003	12.7801	53.5433	122.303
2019/8/15 21:32	46	7.70008	36.9	6.91694	22.2883	900	9.72003	12.7801	53.7405	123.003
2019/8/15 21:32	135	7.71008	36.7	6.91694	22.4368	900	9.72003	12.7801	53.9404	123.653
2019/8/15 21:33	32	7.72008	35.6	6.91694	26.3001	900	9.72003	12.7801	54.1404	124.453
2019/8/15 21:34	312	7.73008	34.5	6.91694	32.2437	900	9.72003	12.7801	54.3403	125.153
2019/8/15 21:34	396	7.74008	33.9	6.92733	36.9985	900	9.72003	12.7801	54.5403	125.853
2019/8/15 21:35	477	7.75008	33.3	6.92733	41.4562	900	9.72003	12.7801	54.7402	126.453
2019/8/15 21:35	562	7.76008	32.8	6.92733	44.7251	900	9.72003	12.7801	54.943	127.053
2019/8/15 21:36	651	7.77008	32.4	6.92733	46.211	900	9.72003	12.7801	55.1429	127.853
2019/8/15 21:37	732	7.78008	32	6.92733	49.0342	900	9.72003	12.7801	55.3429	128.603
2019/8/15 21:37	623	7.79008	31.7	6.91694	49.1828	900	9.72003	12.7801	55.5428	129.303
2019/8/15 21:38	708	7.80008	30.9	6.91694	49.7771	900	9.72003	12.7801	55.765	129.953
2019/8/15 21:38	0	7.81008	30.1	6.91694	45.9138	900	9.72003	12.7801	56.0051	130.603
2019/8/15 21:39	78	7.82008	29.6	6.91694	42.4963	900	9.72003	12.7801	56.2418	131.253
2019/8/15 21:40	164	7.83008	29.1	6.91694	41.0104	900	9.72003	12.7801	56.4819	131.953
2019/8/15 21:40	252	7.84008	28.8	6.91694	42.0505	900	9.72003	12.7801	56.7219	132.653
2019/8/15 21:41	339	7.85008	28.5	6.91694	44.2793	900	9.72003	12.7801	56.962	133.304
2019/8/15 21:41	422	7.86009	28.2	6.90656	45.6166	900	9.72003	12.7801	57.202	133.954
2019/8/15 21:42	508	7.87009	28.2	6.90656	43.5364	900	9.72003	12.7801	57.4753	134.654
2019/8/15 21:43	593	7.88009	28.2	6.90656	35.9584	900	9.72003	12.7801	57.759	135.404
2019/8/15 21:43	676	7.89009	28.1	6.90656	32.9866	900	9.72003	12.7801	58.0389	136.104
2019/8/15 21:44	758	7.90009	28.3	6.90656	27.9346	900	9.72003	12.9001	58.336	136.754
2019/8/15 21:44	842	7.91009	28	6.90656	24.6657	900	9.72003	12.9201	58.636	137.404
2019/8/15 21:45	648	7.92009	28.2	6.90656	21.2481	900	9.72003	13.0201	58.9359	138.004
2019/8/15 21:46	10	7.93009	28.2	6.90656	17.0877	900	9.72003	13.0601	59.2316	138.704
2019/8/15 21:46	100	7.94009	28	6.90656	17.3848	900	9.72003	13.0801	59.5316	139.454
2019/8/15 21:47	0	7.95009	28.2	6.90656	14.4131	900	9.72003	13.2001	59.8315	140.204
2019/8/15 21:47	276	7.96009	28.1	6.90656	12.0357	900	9.72003	13.2801	60.1314	140.854
2019/8/15 21:48	367	7.97009	28.2	6.90656	10.6984	900	9.72003	13.3001	60.4314	141.454
2019/8/15 21:49	273	7.98009	28.2	6.90656	9.36107	900	9.72003	13.4201	60.7313	142.154
2019/8/15 21:49	362	7.99009	28.1	6.90656	9.21248	900	9.72003	13.4801	61.0354	142.904
2019/8/15 21:50	635	8.00009	28.3	6.90656	7.42942	900	9.72003	13.5001	61.3353	143.604

2019/8/15 21:50	717	8.01009	28.1	6.90656	6.68648	900	9.72003	13.5601	61.6352	144.254
2019/8/15 21:51	803	8.02009	28.3	6.90656	6.53789	900	9.72003	13.6001	61.9352	144.954
2019/8/15 21:52	891	8.03009	28.1	6.90656	5.20059	900	9.72003	13.6601	62.2351	145.654
2019/8/15 21:52	977	8.04009	27.7	6.90656	6.68648	900	9.72003	13.6601	62.535	146.304
2019/8/15 21:53	63	8.05009	27.1	6.90656	9.21248	900	9.72003	13.6601	62.8266	146.904
2019/8/15 21:53	146	8.06009	26.7	6.90656	16.4933	900	9.72003	13.6601	63.0765	147.654
2019/8/15 21:54	14	8.07009	26.2	6.90656	22.1397	900	9.72003	13.6801	63.3265	148.354
2019/8/15 21:55	319	8.08009	25.8	6.90656	26.4487	900	9.72003	13.7001	63.5764	149.104
2019/8/15 21:55	404	8.09009	25.6	6.90656	29.5691	900	9.72003	13.7201	63.8264	149.705
2019/8/15 21:56	485	8.10009	25.3	6.90656	31.6493	900	9.72003	13.7201	64.0798	150.305
2019/8/15 21:56	308	8.11009	25.1	6.90656	33.2838	900	9.72003	13.7401	64.3297	150.955
2019/8/15 21:57	658	8.12009	25.3	6.90656	32.5409	900	9.72003	13.8401	64.5797	151.705
2019/8/15 21:58	743	8.13009	25.1	6.90656	31.5007	900	9.72003	13.8401	64.8296	152.505
2019/8/15 21:58	827	8.14009	25.2	6.90656	31.055	900	9.72003	13.8401	65.0795	153.255
2019/8/15 21:59	910	8.15009	25.1	6.90656	30.0149	900	9.72003	13.8601	65.3295	153.955
2019/8/15 21:59	995	8.16009	25.2	6.90656	25.4086	850	9.72003	13.9201	65.5794	154.605
2019/8/15 22:00	77	8.17009	25.3	6.90656	20.0594	850	9.72003	13.9201	65.8259	155.305
2019/8/15 22:01	157	8.18009	25.1	6.90656	19.1679	850	9.72003	14.0201	66.0758	156.005
2019/8/15 22:01	252	8.19009	25.3	6.90656	17.0877	850	9.72003	14.0601	66.3258	156.755
2019/8/15 22:02	342	8.20009	25.1	6.90656	16.6419	850	9.72003	14.1601	66.5757	157.455
2019/8/15 22:02	427	8.21009	25.3	6.90656	15.7504	850	9.72003	14.1601	66.8291	158.155
2019/8/15 22:03	512	8.22009	25.1	6.90656	14.7103	850	9.72003	14.2401	67.079	158.805
2019/8/15 22:04	602	8.23009	25.2	6.90656	13.2244	850	9.72003	14.2601	67.329	159.455
2019/8/15 22:04	686	8.24009	25.2	6.90656	12.3328	850	9.72003	14.2601	67.5789	160.105
2019/8/15 22:05	768	8.25009	25.1	6.90656	12.63	850	9.72003	14.2601	67.8289	160.855
2019/8/15 22:05	852	8.26009	25.2	6.90656	10.6984	850	9.72003	14.3801	68.0788	161.605
2019/8/15 22:06	939	8.27009	25.1	6.90656	11.2927	850	9.72003	14.3801	68.3322	162.305
2019/8/15 22:07	23	8.28009	25.2	6.90656	9.65825	850	9.72003	14.4001	68.5752	162.955
2019/8/15 22:07	112	8.29009	25.1	6.90656	9.80684	850	9.72003	14.4001	68.8155	163.605
2019/8/15 22:08	193	8.30009	25.2	6.90656	12.4814	850	9.72003	14.4001	69.0352	164.305
2019/8/15 22:08	275	8.31009	25.1	6.90656	13.8187	850	9.72003	14.5201	69.2549	164.905
2019/8/15 22:09	358	8.3201	25.2	6.90656	16.0475	850	9.72003	14.5201	69.4681	165.656
2019/8/15 22:10	445	8.3301	25.2	6.90656	18.2764	850	9.72003	14.5201	69.668	166.356
2019/8/15 22:10	528	8.3401	25.1	6.90656	22.2883	850	9.72003	14.5201	69.8708	167.006
2019/8/15 22:11	610	8.3501	25.3	6.90656	25.4086	850	9.72003	14.5201	70.0707	167.656

2019/8/15 22:11	690	8.3601	25.1	6.90656	29.1233	850	9.72003	14.5201	70.2707	168.306
2019/8/15 22:12	773	8.3701	25.2	6.90656	32.2437	850	9.72003	14.5201	70.4706	169.006
2019/8/15 22:13	862	8.3801	25.1	6.90656	34.4725	850	9.72003	14.5201	70.6706	169.756
2019/8/15 22:13	946	8.3901	25.2	6.90656	35.6612	850	9.72003	14.5201	70.8733	170.506
2019/8/15 22:14	29	8.4001	25.2	6.90656	35.5126	850	9.72003	14.5401	71.0677	171.156
2019/8/15 22:14	109	8.4101	25.1	6.90656	37.5929	850	9.72003	14.5401	71.2676	171.806
2019/8/15 22:15	197	8.4201	25.2	6.90656	37.89	850	9.72003	14.7801	71.4676	172.456
2019/8/15 22:16	282	8.4301	25.1	6.90656	37.1471	850	9.72003	14.7801	71.6675	173.206
2019/8/15 22:16	363	8.4401	25.1	6.90656	37.2957	850	9.72003	14.8001	71.8675	173.956
2019/8/15 22:17	448	8.4501	25.3	6.90656	35.5126	850	9.72003	14.8602	72.0702	174.656
2019/8/15 22:17	527	8.4601	25.1	6.90656	35.5126	850	9.72003	14.9002	72.2702	175.306
2019/8/15 22:18	616	8.4701	25.3	6.90656	34.0267	850	9.72003	14.9802	72.4701	175.956
2019/8/15 22:19	701	8.4801	25.1	6.90656	31.7979	850	9.72003	15.0602	72.6701	176.706
2019/8/15 22:19	788	8.4901	25.2	6.90656	30.7578	850	9.72003	15.0802	72.87	177.456
2019/8/15 22:20	866	8.5001	25.2	6.90656	27.1917	850	9.72003	15.1002	73.07	178.156
2019/8/15 22:20	954	8.5101	25.1	6.90656	26.3001	850	9.72003	15.1802	73.2727	178.806
2019/8/15 22:21	35	8.5201	25.3	6.90656	23.7741	850	9.72003	15.2202	73.4671	179.456
2019/8/15 22:22	118	8.5301	25.1	6.90656	22.2883	850	9.72003	15.2402	73.6671	180.206
2019/8/15 22:22	206	8.5401	25.1	6.90656	20.208	850	9.72003	15.2802	73.867	180.906
2019/8/15 22:23	292	8.5501	25.2	6.90656	16.4933	850	9.72003	15.3002	74.067	181.556
2019/8/15 22:23	378	8.5601	25.1	6.90656	15.3046	850	9.72003	15.3402	74.2669	182.207
2019/8/15 22:24	466	8.5701	25.3	6.90656	10.847	850	9.72003	15.4602	74.4696	182.857
2019/8/15 22:25	550	8.5801	25.1	6.90656	8.9153	850	9.72003	15.4802	74.6696	183.557
2019/8/15 22:25	634	8.5901	25.2	6.90656	6.68648	850	9.72003	15.5202	74.8695	184.307
2019/8/15 22:26	724	8.6001	25.2	6.90656	2.97177	850	9.72003	15.6402	75.0582	184.957
2019/8/15 22:26	804	8.6101	25.2	6.90656	3.12036	850	9.72003	15.6402	75.2384	185.607
2019/8/15 22:27	795	8.6201	25.3	6.90656	1.48588	850	9.72003	15.7202	75.4211	186.407
2019/8/15 22:28	979	8.6301	25.1	6.90656	0.89153	850	9.72003	15.7202	75.5894	187.357
2019/8/15 22:28	65	8.6401	25.3	6.90656	0.742942	850	9.72003	15.7402	75.7448	187.657
2019/8/15 22:29	149	8.6501	25.1	6.90656	2.97177	850	9.72003	15.7402	75.9047	187.657
2019/8/15 22:29	0	8.6601	25.3	6.90656	3.41753	850	9.72003	15.7402	76.0645	187.657
2019/8/15 22:30	316	8.6701	25.1	6.90656	4.90342	850	9.72003	15.7402	76.2185	187.657
2019/8/15 22:31	399	8.6801	25.2	6.91694	9.50966	850	9.72003	15.7402	76.3705	187.657
2019/8/15 22:31	494	8.6901	25.2	6.91694	11.4413	850	9.72003	15.7402	76.5205	187.657
2019/8/15 22:32	579	8.7001	25.1	6.92733	15.156	850	9.72003	15.7402	76.6704	187.657

2019/8/15 22:32	668	8.7101	25.3	6.92733	17.5334	850	9.72003	15.7402	76.8204	187.657
2019/8/15 22:33	753	8.7201	25.1	6.92733	19.7623	850	9.72003	15.7402	76.9704	187.657
2019/8/15 22:34	846	8.7301	25.2	6.93771	23.1798	850	9.72003	15.7402	77.1203	187.657
2019/8/15 22:34	931	8.7401	25.2	6.93771	22.5854	850	9.72003	15.7402	77.2724	187.657
2019/8/15 22:35	18	8.7501	25.1	6.93771	24.0713	850	9.72003	15.7402	77.4182	187.657
2019/8/15 22:35	0	8.76011	25.2	6.93771	24.8143	850	9.72003	15.7402	77.5681	187.657
2019/8/15 22:36	178	8.77011	25.1	6.93771	26.4487	850	9.78003	15.7402	77.7181	187.657
2019/8/15 22:37	258	8.78011	25.2	6.93771	27.0431	850	9.78003	15.7402	77.8681	187.657
2019/8/15 22:37	129	8.79011	25.1	6.93771	27.786	850	9.78003	15.7402	78.0201	187.657
2019/8/15 22:38	208	8.80011	25.1	6.93771	29.4205	850	9.78003	15.7402	78.1701	187.657
2019/8/15 22:38	518	8.81011	25.2	6.93771	28.529	850	9.80003	15.7402	78.32	187.657
2019/8/15 22:39	603	8.82011	25.1	6.93771	34.1753	850	9.84004	15.7402	78.4242	187.657
2019/8/15 22:40	693	8.83011	25.1	6.93771	41.6048	850	9.96004	15.7402	78.5241	187.657
2019/8/15 22:40	780	8.84011	25.2	6.93771	43.2392	850	9.96004	15.7402	78.6241	187.657
2019/8/15 22:41	864	8.85011	25.1	6.93771	45.0223	850	10	15.7402	78.7255	187.657
2019/8/15 22:41	952	8.86011	25.3	6.93771	42.1991	850	10.06	15.7402	78.8255	187.657
2019/8/15 22:42	36	8.87011	25.1	6.93771	40.2675	850	10.06	15.7402	78.9227	187.657
2019/8/15 22:43	116	8.88011	25.2	6.93771	42.942	850	10.06	15.7402	79.0226	187.657
2019/8/15 22:43	203	8.89011	25.2	6.93771	43.9822	850	10.06	15.7402	79.1226	187.657
2019/8/15 22:44	295	8.90011	25.1	6.93771	46.3596	850	10.06	15.7402	79.2226	187.657
2019/8/15 22:44	376	8.91011	25.1	6.93771	46.8053	850	10.06	15.7402	79.324	187.657
2019/8/15 22:45	469	8.92011	25.2	6.93771	43.685	850	10.06	15.7402	79.4239	187.657
2019/8/15 22:46	553	8.93011	25.1	6.93771	45.1709	850	10.06	15.7402	79.5239	187.657
2019/8/15 22:46	446	8.94011	25.2	6.93771	46.211	850	10.06	15.7402	79.6239	187.657
2019/8/15 22:47	581	8.95011	25.1	6.93771	46.9539	850	10.06	15.7402	79.7239	187.657
2019/8/15 22:47	807	8.96011	25.1	6.93771	48.8856	850	10.06	15.7402	79.8238	187.657
2019/8/15 22:48	894	8.97011	25.3	6.93771	48.8856	850	10.06	15.7402	79.9252	187.657
2019/8/15 22:49	976	8.98011	25.1	6.93771	49.0342	850	10.06	15.7402	80.0252	187.657
2019/8/15 22:49	60	8.99011	25.2	6.93771	49.9257	800	10.06	15.7402	80.1224	187.657
2019/8/15 22:50	143	9.00011	25.3	6.93771	43.685	800	10.06	15.7402	80.2223	187.657
2019/8/15 22:50	226	9.01011	25.1	6.93771	43.685	800	10.06	15.7402	80.3223	187.657
2019/8/15 22:51	307	9.02011	25.1	6.93771	45.0223	800	10.06	15.7402	80.4237	187.657
2019/8/15 22:52	398	9.03011	25.3	6.93771	44.7251	800	10.06	15.7402	80.5237	187.657
2019/8/15 22:52	481	9.04011	25.1	6.93771	45.0223	800	10.06	15.7402	80.6236	187.657
2019/8/15 22:53	564	9.05011	25.1	6.93771	46.3596	800	10.06	15.7402	80.7236	187.657

2019/8/15 22:53	649	9.06011	25.3	6.93771	45.3195	800	10.06	15.7402	80.8236	187.657
2019/8/15 22:54	732	9.07011	25.1	6.93771	44.7251	800	10.06	15.7402	80.9236	187.657
2019/8/15 22:55	816	9.08011	25.1	6.93771	45.7652	800	10.06	15.7402	81.0249	187.657
2019/8/15 22:55	897	9.09011	25.2	6.93771	47.8455	800	10.08	15.7402	81.1249	187.657
2019/8/15 22:56	979	9.10011	25.1	6.93771	47.3997	800	10.08	15.7402	81.2249	187.657
2019/8/15 22:56	58	9.11011	25.1	6.93771	48.2912	800	10.08	15.7402	81.3221	187.657
2019/8/15 22:57	145	9.12011	25.2	6.93771	48.8856	800	10.12	15.7402	81.4221	187.657
2019/8/15 22:58	223	9.13011	25.2	6.93771	47.6969	800	10.12	15.7402	81.522	187.657
2019/8/15 22:58	310	9.14011	25	6.93771	48.5884	800	10.14	15.7402	81.622	187.657
2019/8/15 22:59	395	9.15011	25.2	6.93771	49.1828	800	10.14	15.7402	81.7234	187.657
2019/8/15 22:59	484	9.16011	25.2	6.93771	50.5201	800	10.14	15.7402	81.8233	187.657
2019/8/15 23:00	569	9.17011	25.1	6.93771	51.5602	800	10.14	15.7402	81.9233	187.657
2019/8/15 23:01	656	9.18011	25.1	6.93771	51.7088	800	10.14	15.7402	82.0233	187.657
2019/8/15 23:01	741	9.19011	25.2	6.93771	51.7088	800	10.14	15.7402	82.1233	187.657
2019/8/15 23:02	826	9.20012	25.1	6.93771	51.4116	800	10.14	15.7402	82.2246	187.657
2019/8/15 23:02	916	9.21012	25.2	6.93771	51.7088	800	10.14	15.7402	82.3246	187.657
2019/8/15 23:03	996	9.22012	25.2	6.93771	52.6003	800	10.14	15.7402	82.4246	187.657
2019/8/15 23:04	86	9.23012	25.1	6.93771	53.1946	800	10.14	15.7402	82.5218	187.657
2019/8/15 23:04	169	9.24012	25.1	6.93771	54.0862	800	10.16	15.7402	82.6218	187.657
2019/8/15 23:05	249	9.25012	25.2	6.93771	54.6805	800	10.16	15.7402	82.7217	187.657
2019/8/15 23:05	332	9.26012	25.1	6.93771	54.3834	800	10.16	15.7402	82.8231	187.657
2019/8/15 23:06	413	9.27012	25.1	6.93771	54.6805	800	10.18	15.7402	82.9231	187.657
2019/8/15 23:07	498	9.28012	25.3	6.93771	53.9376	800	10.2	15.7402	83.023	187.657
2019/8/15 23:07	589	9.29012	25.1	6.93771	53.9376	800	10.2	15.7402	83.123	187.657
2019/8/15 23:08	502	9.30012	25.1	6.93771	55.8692	800	10.2	15.7402	83.223	187.907
2019/8/15 23:08	656	9.31012	25.2	6.93771	54.0862	750	10.2	15.7402	83.323	188.507
2019/8/15 23:09	845	9.32012	25.2	6.93771	46.6568	750	10.2	15.7402	83.4243	189.107
2019/8/15 23:10	935	9.33012	25.1	6.93771	43.8336	750	10.2	15.7402	83.5243	189.557
2019/8/15 23:10	16	9.34012	25.1	6.93771	42.6449	750	10.2	15.7402	83.6215	190.157
2019/8/15 23:11	102	9.35012	25.3	6.93771	40.7132	750	10.2	15.7402	83.7215	190.607
2019/8/15 23:11	192	9.36012	25.1	6.92733	40.5646	750	10.2	15.7402	83.8215	191.207
2019/8/15 23:12	276	9.37012	25.1	6.92733	42.0505	750	10.2	15.7402	83.9214	191.657
2019/8/15 23:13	363	9.38012	25.2	6.92733	41.0104	750	10.2	15.7402	84.0228	192.257
2019/8/15 23:13	447	9.39012	25.1	6.92733	42.1991	750	10.2	15.7402	84.1228	192.907
2019/8/15 23:14	532	9.40012	25.1	6.92733	42.1991	750	10.2	15.7402	84.2228	193.357

2019/8/15 23:14	621	9.41012	25.3	6.92733	41.6048	750	10.2	15.7402	84.3227	193.957
2019/8/15 23:15	704	9.42012	25.1	6.92733	41.7533	750	10.2	15.7402	84.4227	194.457
2019/8/15 23:16	785	9.43012	25.1	6.92733	41.9019	750	10.2	15.7402	84.5227	195.007
2019/8/15 23:16	869	9.44012	25.2	6.92733	42.4963	750	10.2	15.7402	84.624	195.607
2019/8/15 23:17	446	9.45012	25.1	6.92733	42.3477	750	10.2	15.7402	84.7226	196.057
2019/8/15 23:17	530	9.46012	25.2	6.92733	43.2392	750	10.2	15.7402	84.8226	196.707
2019/8/15 23:18	610	9.47012	25.2	6.92733	42.4963	750	10.2	15.7402	84.9226	197.157
2019/8/15 23:19	697	9.48012	25.1	6.92733	42.3477	750	10.2	15.7402	85.0226	197.757
2019/8/15 23:19	781	9.49012	25.1	6.92733	43.685	750	10.2	15.7402	85.1239	198.357
2019/8/15 23:20	863	9.50012	25.2	6.92733	42.6449	750	10.2	15.7402	85.2239	198.808
2019/8/15 23:20	966	9.51012	25.1	6.92733	41.4562	750	10.2	15.7402	85.3406	199.408
2019/8/15 23:21	54	9.52012	25.1	6.91694	39.8217	750	10.2	15.7402	85.4768	199.908
2019/8/15 23:22	138	9.53012	25.1	6.91694	36.107	750	10.2	15.7402	85.6168	200.508
2019/8/15 23:22	224	9.54012	25.1	6.91694	32.838	750	10.2	15.7402	85.7569	201.108
2019/8/15 23:23	311	9.55012	25	6.91694	32.2437	750	10.2	15.7402	85.8989	201.558
2019/8/15 23:23	401	9.56012	25.1	6.90656	31.3522	750	10.2	15.7402	86.039	202.158
2019/8/15 23:24	485	9.57012	25.3	6.90656	29.4205	750	10.2	15.7402	86.1791	202.608
2019/8/15 23:25	573	9.58012	25.1	6.90656	29.8663	750	10.2	15.7402	86.3192	203.208
2019/8/15 23:25	654	9.59012	25.1	6.90656	29.8663	750	10.2	15.7402	86.4592	203.808
2019/8/15 23:26	741	9.60012	25.3	6.90656	28.9747	750	10.2	15.7402	86.6013	204.258
2019/8/15 23:26	826	9.61012	25.1	6.90656	29.2719	750	10.2	15.8202	86.7413	204.858
2019/8/15 23:27	908	9.62012	25.2	6.90656	29.2719	750	10.2	15.8202	86.8814	205.308
2019/8/15 23:28	992	9.63013	25.2	6.90656	28.6776	750	10.2	15.8802	87.0215	205.908
2019/8/15 23:28	75	9.64013	25.1	6.90656	30.1634	750	10.2	15.9802	87.1577	206.358
2019/8/15 23:29	165	9.65013	25.1	6.90656	30.7578	750	10.2	15.9802	87.2978	206.958
2019/8/15 23:29	249	9.66013	25.2	6.90656	29.8663	750	10.2	16.0802	87.4378	207.558
2019/8/15 23:30	330	9.67013	25.1	6.90656	30.7578	750	10.2	16.1002	87.5798	208.008
2019/8/15 23:31	413	9.68013	25.1	6.90656	31.6493	750	10.2	16.1402	87.7199	208.608
2019/8/15 23:31	501	9.69013	25.2	6.90656	30.9064	750	10.2	16.2202	87.86	209.108
2019/8/15 23:32	586	9.70013	25	6.90656	31.2036	750	10.2	16.2802	88.0001	209.658
2019/8/15 23:32	674	9.71013	25.1	6.90656	31.6493	750	10.2	16.3002	88.1402	210.258
2019/8/15 23:33	756	9.72013	25.2	6.90656	31.2036	750	10.2	16.3402	88.2822	210.708
2019/8/15 23:34	834	9.73013	25.2	6.90656	30.6092	750	10.2	16.4202	88.4222	211.308
2019/8/15 23:34	918	9.74013	25.1	6.90656	31.3522	750	10.2	16.4602	88.5623	211.808
2019/8/15 23:35	4	9.75013	25.2	6.90656	31.3522	750	10.2	16.4602	88.6985	212.358

2019/8/15 23:35	89	9.76013	25.1	6.90656	30.7578	750	10.2	16.5002	88.8386	213.008
2019/8/15 23:36	170	9.77013	25.1	6.90656	32.2437	750	10.2	16.6402	88.9787	213.458
2019/8/15 23:37	252	9.78013	25.2	6.90656	32.9866	750	10.2	16.6402	89.1207	214.058
2019/8/15 23:37	337	9.79013	25.1	6.90656	32.2437	750	10.2	16.7002	89.2608	214.508
2019/8/15 23:38	427	9.80013	25.1	6.90656	33.1352	750	10.2	16.7202	89.4008	215.109
2019/8/15 23:38	513	9.81013	25.2	6.90656	33.1352	750	10.2	16.8602	89.5409	215.709
2019/8/15 23:39	599	9.82013	25.2	6.90656	32.838	750	10.2	16.9202	89.681	216.159
2019/8/15 23:40	681	9.83013	25	6.90656	33.7296	750	10.2	16.9402	89.823	216.759
2019/8/15 23:40	767	9.84013	25.1	6.90656	34.3239	750	10.2	17.1002	89.9631	217.209
2019/8/15 23:41	855	9.85013	25.3	6.90656	34.3239	750	10.2	17.1802	90.1032	217.809
2019/8/15 23:41	937	9.86013	25.1	6.90656	34.3239	750	10.2	17.3002	90.2432	218.409
2019/8/15 23:42	21	9.87013	25.2	6.90656	35.2155	750	10.2	17.3002	90.3794	218.859
2019/8/15 23:43	105	9.88013	25.3	6.90656	35.5126	750	10.2	17.3002	90.5195	219.459
2019/8/15 23:43	186	9.89013	25.1	6.90656	35.9584	750	10.2	17.3402	90.6615	219.909
2019/8/15 23:44	268	9.90013	25.1	6.90656	36.9985	750	10.2	17.3802	90.8016	220.509
2019/8/15 23:44	355	9.91013	25.2	6.90656	36.9985	750	10.2	17.5602	90.9417	221.109
2019/8/15 23:45	437	9.92013	25.1	6.90656	36.7013	750	10.2	17.5602	91.0817	221.559
2019/8/15 23:46	519	9.93013	25.1	6.90656	37.5929	750	10.2	17.5602	91.2218	222.159
2019/8/15 23:46	486	9.94013	25.1	6.90656	37.89	750	10.2	17.5802	91.3619	222.609
2019/8/15 23:47	698	9.95013	25.2	6.90656	36.9985	750	10.2	17.6402	91.502	223.209
2019/8/15 23:47	779	9.96013	25.1	6.90656	38.1872	750	10.2	17.7002	91.644	223.809
2019/8/15 23:48	868	9.97013	25.1	6.90656	38.4844	750	10.2	18.0202	91.7841	224.309
2019/8/15 23:49	951	9.98013	25.2	6.90656	38.1872	750	10.2	18.0202	91.9241	224.909
2019/8/15 23:49	38	9.99013	25.1	6.90656	39.0788	750	10.2	18.0202	92.0603	225.359
2019/8/15 23:50	125	10.0001	25.2	6.90656	39.9703	750	10.2	18.0602	92.2004	225.959
2019/8/15 23:50	213	10.0101	25.2	6.90656	39.8217	750	10.2	18.0802	92.3424	226.409
2019/8/15 23:51	303	10.0201	25.1	6.90656	40.8618	750	10.2	18.1602	92.4825	227.009
2019/8/15 23:52	387	10.0301	25.1	6.90656	41.7533	750	10.2	18.4402	92.6226	227.609
2019/8/15 23:52	466	10.0401	25.3	6.90656	41.4562	750	10.2	18.6402	92.7626	228.059
2019/8/15 23:53	552	10.0501	25	6.90656	41.7533	750	10.2	18.6402	92.9027	228.659
2019/8/15 23:53	641	10.0601	25.1	6.90656	43.2392	750	10.2	18.6602	93.0428	229.109
2019/8/15 23:54	726	10.0701	25.2	6.90656	43.5364	750	10.2	18.7802	93.1848	229.709
2019/8/15 23:55	809	10.0801	25.2	6.90656	42.6449	750	10.2	18.8202	93.3249	230.309
2019/8/15 23:55	893	10.0901	25.1	6.90656	43.8336	750	10.2	18.8202	93.465	230.759
2019/8/15 23:56	981	10.1001	25.2	6.90656	45.0223	750	10.2	18.8402	93.605	231.36

2019/8/15 23:56	64	10.1101	25.3	6.90656	44.7251	750	10.2	18.9802	93.7412	231.81
2019/8/15 23:57	146	10.1201	25.1	6.90656	45.1709	750	10.2	18.9802	93.8813	232.41
2019/8/15 23:58	227	10.1301	25.1	6.90656	46.3596	750	10.2	18.9802	94.0233	233.01
2019/8/15 23:58	59	10.1401	25.2	6.90656	45.9138	750	10.2	18.9802	94.1634	233.46
2019/8/15 23:59	398	10.1501	25.1	6.90656	45.6166	750	10.2	19.1202	94.3035	234.06
2019/8/15 23:59	480	10.1601	25.1	6.90656	46.8053	750	10.2	19.1202	94.4436	234.51
2019/8/16 0:00	561	10.1701	25.2	6.90656	46.8053	750	10.2	19.1202	94.5836	235.11
2019/8/16 0:01	639	10.1801	25.2	6.90656	46.8053	750	10.2	19.1602	94.7257	235.76
2019/8/16 0:01	725	10.1901	25.1	6.90656	47.6969	750	10.2	19.3603	94.8657	236.21
2019/8/16 0:02	812	10.2001	25.1	6.90656	48.4398	750	10.2	19.3603	95.0058	236.81
2019/8/16 0:02	893	10.2101	25.2	6.90656	47.9941	750	10.2	19.3603	95.1459	237.26
2019/8/16 0:03	972	10.2201	25.1	6.90656	48.5884	750	10.2	19.4203	95.286	237.86
2019/8/16 0:04	60	10.2301	25.1	6.90656	49.4799	750	10.2	19.4203	95.4221	238.36
2019/8/16 0:04	145	10.2401	25.3	6.90656	49.1828	750	10.2	19.4203	95.5642	238.91
2019/8/16 0:05	239	10.2501	25.1	6.90656	49.7771	750	10.2	19.4803	95.7042	239.51
2019/8/16 0:05	324	10.2601	25.1	6.90656	50.8172	750	10.2	19.4803	95.8443	239.96
2019/8/16 0:06	412	10.2701	25.2	6.90656	50.6686	750	10.2	19.5003	95.9844	240.56
2019/8/16 0:07	499	10.2801	25.1	6.90656	50.0743	750	10.2	19.5603	96.1245	241.11
2019/8/16 0:07	579	10.2901	25	6.90656	51.4116	750	10.2	19.5803	96.2645	241.61
2019/8/16 0:08	670	10.3001	25.1	6.90656	52.1545	750	10.2	19.6203	96.4066	242.21
2019/8/16 0:08	757	10.3101	25.2	6.90656	52.0059	750	10.2	19.6603	96.5466	242.71
2019/8/16 0:09	841	10.3201	25.1	6.90656	51.5602	750	10.2	19.7803	96.6867	243.31
2019/8/16 0:10	928	10.3301	25.1	6.90656	52.6003	750	10.2	19.7803	96.8268	243.91
2019/8/16 0:10	16	10.3401	25.2	6.90656	52.7489	750	10.2	19.9003	96.963	244.36
2019/8/16 0:11	100	10.3501	25.2	6.90656	52.4517	750	10.2	20.0203	97.1031	244.96
2019/8/16 0:11	190	10.3601	25.1	6.90656	53.0461	750	10.2	20.0203	97.2451	245.41
2019/8/16 0:12	273	10.3701	25.1	6.90656	53.789	750	10.2	20.0403	97.3851	246.01
2019/8/16 0:13	357	10.3801	25.2	6.90656	53.3432	750	10.2	20.0603	97.5252	246.46
2019/8/16 0:13	439	10.3901	25.1	6.90656	53.0461	750	10.2	20.2403	97.6653	247.06
2019/8/16 0:14	520	10.4001	25.1	6.90656	53.9376	750	10.2	20.2803	97.8054	247.661
2019/8/16 0:14	602	10.4101	25.2	6.90656	54.3834	750	10.2	20.3403	97.9474	248.111
2019/8/16 0:15	687	10.4201	25.3	6.90656	53.9376	750	10.2	20.4803	98.0875	248.711
2019/8/16 0:16	769	10.4301	25.1	6.90656	54.2348	750	10.2	20.5003	98.2275	249.161
2019/8/16 0:16	853	10.4401	25.1	6.90656	54.5319	750	10.2	20.5403	98.3676	249.811
2019/8/16 0:17	936	10.4501	25.2	6.89618	54.8291	750	10.2	20.5803	98.5077	250.411

2019/8/16 0:17	27	10.4601	25.1	6.90656	55.2749	750	10.2	20.6403	98.6439	250.861
2019/8/16 0:18	109	10.4701	25.1	6.90656	56.0178	750	10.2	20.6403	98.7859	251.461
2019/8/16 0:19	344	10.4801	25.2	6.90656	56.1664	750	10.2	20.6403	98.926	251.911
2019/8/16 0:19	428	10.4901	25.2	6.90656	55.4235	750	10.2	20.7203	99.0661	252.511
2019/8/16 0:20	516	10.5001	25	6.90656	56.315	750	10.2	20.7803	99.2061	253.161
2019/8/16 0:20	599	10.5101	25.1	6.90656	56.9094	750	10.2	20.7803	99.3482	253.611
2019/8/16 0:21	687	10.5201	25.2	6.90656	57.2065	750	10.2	20.8203	99.4882	254.211
2019/8/16 0:22	769	10.5301	25.2	6.91694	56.6122	750	10.2	21.0403	99.6283	254.711
2019/8/16 0:22	851	10.5401	25.1	6.91694	56.9094	750	10.2	21.0403	99.7684	255.311
2019/8/16 0:23	935	10.5501	25.1	6.91694	57.5037	750	10.2	21.0403	99.9085	255.911
2019/8/16 0:23	14	10.5601	25.2	6.90656	57.5037	750	10.2	21.0403	100.045	256.361
2019/8/16 0:24	100	10.5701	25.2	6.90656	56.9094	750	10.2	21.0403	100.187	256.961
2019/8/16 0:25	185	10.5801	25	6.90656	57.9495	750	10.2	21.0403	100.327	257.411
2019/8/16 0:25	269	10.5901	25.1	6.90656	58.3952	750	10.2	21.0603	100.467	258.011
2019/8/16 0:26	354	10.6001	25.2	6.90656	58.5438	750	10.2	21.0603	100.607	258.51
2019/8/16 0:26	442	10.6101	25.2	6.90656	57.8009	750	10.2	21.1203	100.747	259.06
2019/8/16 0:27	526	10.6201	25.1	6.90656	58.6924	750	10.2	21.1203	100.887	259.71
2019/8/16 0:28	608	10.6301	25.1	6.90656	58.9896	750	10.2	21.3003	101.029	260.16
2019/8/16 0:28	691	10.6401	25.1	6.90656	58.841	750	10.2	21.3003	101.169	260.76
2019/8/16 0:29	776	10.6501	25.2	6.90656	57.8009	750	10.2	21.3003	101.309	261.26
2019/8/16 0:29	858	10.6601	25	6.90656	58.9896	750	10.2	21.3003	101.449	261.81
2019/8/16 0:30	945	10.6701	25.1	6.90656	59.7325	750	10.2	21.3003	101.589	262.409
2019/8/16 0:31	26	10.6801	25.2	6.90656	59.584	750	10.2	21.5803	101.737	262.859
2019/8/16 0:31	111	10.6901	25.1	6.90656	58.9896	750	10.2	21.5803	101.899	263.459
2019/8/16 0:32	200	10.7001	25.1	6.90656	60.0297	750	10.2	21.5803	102.059	263.909
2019/8/16 0:32	285	10.7101	25.2	6.90656	60.1783	750	10.2	21.6803	102.219	264.509
2019/8/16 0:33	367	10.7201	25.2	6.90656	56.4636	700	10.2	21.7603	102.378	265.109
2019/8/16 0:34	450	10.7302	25.1	6.90656	52.3031	700	10.2	21.7803	102.538	265.559
2019/8/16 0:34	535	10.7402	25.1	6.90656	52.0059	700	10.2	21.9203	102.698	266.159
2019/8/16 0:35	617	10.7502	25.1	6.90656	51.7088	700	10.2	21.9203	102.86	266.608
2019/8/16 0:35	697	10.7602	25.1	6.90656	51.1144	700	10.2	21.9203	103.02	267.208
2019/8/16 0:36	785	10.7702	25	6.90656	52.3031	700	10.2	21.9203	103.18	267.808
2019/8/16 0:37	868	10.7802	25.1	6.90656	52.7489	700	10.2	21.9403	103.34	268.258
2019/8/16 0:37	955	10.7902	25.2	6.90656	52.0059	700	10.2	21.9403	103.5	268.858
2019/8/16 0:38	37	10.8002	25.3	6.90656	51.7088	700	10.2	22.1003	103.655	269.308

2019/8/16 0:38	124	10.8102	25.1	6.90656	51.7088	700	10.2	22.1803	103.817	269.908
2019/8/16 0:39	213	10.8202	25.1	6.90656	52.0059	700	10.2	22.1803	103.977	270.507
2019/8/16 0:40	294	10.8302	25.2	6.90656	52.3031	700	10.2	22.1803	104.137	270.957
2019/8/16 0:40	376	10.8402	25.2	6.90656	52.0059	700	10.2	22.3203	104.297	271.557
2019/8/16 0:41	468	10.8502	25.1	6.90656	52.6003	700	10.2	22.3203	104.456	272.007
2019/8/16 0:41	550	10.8602	25.1	6.90656	53.1946	700	10.2	22.3203	104.616	272.607
2019/8/16 0:42	632	10.8702	25.2	6.90656	52.6003	700	10.2	22.3203	104.778	273.207
2019/8/16 0:43	718	10.8802	25.2	6.90656	52.0059	700	10.2	22.4003	104.938	273.657
2019/8/16 0:43	799	10.8902	25	6.90656	53.9376	700	10.2	22.4203	105.098	274.257
2019/8/16 0:44	879	10.9002	25	6.90656	54.5319	700	10.2	22.5803	105.258	274.706
2019/8/16 0:44	967	10.9102	25.1	6.90656	53.9376	700	10.2	22.6603	105.418	275.306
2019/8/16 0:45	47	10.9202	25.2	6.90656	53.6404	700	10.2	22.6603	105.575	275.756
2019/8/16 0:46	133	10.9302	25.2	6.90656	53.0461	700	10.2	22.7603	105.735	276.356
2019/8/16 0:46	214	10.9402	25.1	6.90656	53.3432	700	10.2	22.8403	105.895	276.956
2019/8/16 0:47	296	10.9502	25.1	6.90656	54.3834	700	10.2	22.9003	106.055	277.406
2019/8/16 0:47	380	10.9602	25.2	6.90656	53.9376	700	10.2	22.9403	106.215	278.006
2019/8/16 0:48	461	10.9702	25.2	6.90656	52.8975	650	10.2	22.9803	106.375	278.456
2019/8/16 0:49	547	10.9802	25.1	6.90656	46.9539	650	10.2	23.0803	106.537	279.055
2019/8/16 0:49	631	10.9902	25.1	6.90656	45.0223	650	10.2	23.1403	106.697	279.655
2019/8/16 0:50	719	11.0002	25.2	6.90656	43.3878	650	10.2	23.2403	106.856	280.105
2019/8/16 0:50	803	11.0102	25.2	6.90656	42.1991	650	10.2	23.4003	107.016	280.755
2019/8/16 0:51	883	11.0202	25	6.90656	42.4963	650	10.2	23.4603	107.176	281.205
2019/8/16 0:52	970	11.0302	24.9	6.90656	43.3878	650	10.2	23.4603	107.336	281.805
2019/8/16 0:52	56	11.0402	25.1	6.90656	43.9822	650	10.2	23.4603	107.494	282.405
2019/8/16 0:53	140	11.0502	25.2	6.90656	43.2392	650	10.2	23.4603	107.654	282.854
2019/8/16 0:53	223	11.0602	25.2	6.90656	42.6449	650	10.2	23.4603	107.813	283.454
2019/8/16 0:54	313	11.0702	25.2	6.90656	42.6449	650	10.2	23.6604	107.973	283.904
2019/8/16 0:55	401	11.0802	25.1	6.90656	43.3878	650	10.2	23.6604	108.133	284.504
2019/8/16 0:55	484	11.0902	25.1	6.90656	43.9822	650	10.2	23.6604	108.293	285.104
2019/8/16 0:56	567	11.1002	25.1	6.90656	43.8336	650	10.2	23.6804	108.455	285.554
2019/8/16 0:56	652	11.1102	25.2	6.90656	42.7935	650	10.2	23.8004	108.615	286.204
2019/8/16 0:57	745	11.1202	25.1	6.90656	42.1991	650	10.2	23.8004	108.775	286.654
2019/8/16 0:58	833	11.1302	25	6.90656	43.8336	650	10.2	23.8004	108.935	287.253
2019/8/16 0:58	920	11.1402	25	6.90656	44.5765	650	10.2	23.8004	109.094	287.903
2019/8/16 0:59	1	11.1502	25.1	6.90656	44.1308	650	10.2	23.8204	109.252	288.353

2019/8/16 0:59	86	11.1602	25.2	6.90656	43.685	650	10.2	23.9604	109.412	288.953
2019/8/16 1:00	167	11.1702	25.2	6.90656	43.3878	650	10.2	23.9804	109.572	289.453
2019/8/16 1:01	253	11.1802	25	6.90656	43.3878	650	10.2	24.0804	109.732	290.053
2019/8/16 1:01	339	11.1902	25	6.90656	44.4279	650	10.2	24.0804	109.891	290.603
2019/8/16 1:02	420	11.2002	25.1	6.90656	44.1308	650	10.2	24.2204	110.051	291.102
2019/8/16 1:02	509	11.2102	25.2	6.90656	43.5364	650	10.2	24.2204	110.211	291.702
2019/8/16 1:03	590	11.2202	25.1	6.90656	42.4963	650	10.2	24.2404	110.373	292.152
2019/8/16 1:04	671	11.2302	25.1	6.90656	43.0906	650	10.2	24.3004	110.533	292.802
2019/8/16 1:04	755	11.2402	25.1	6.90656	43.5364	650	10.2	24.3604	110.693	293.402
2019/8/16 1:05	837	11.2502	25.2	6.90656	43.3878	650	10.2	24.5004	110.853	293.852
2019/8/16 1:05	925	11.2602	25.2	6.90656	42.4963	650	10.2	24.6204	111.013	294.502
2019/8/16 1:06	8	11.2602	25	6.90656	42.7935	650	10.2	24.6604	111.17	294.952
2019/8/16 1:07	98	11.2702	25.1	6.90656	43.0906	650	10.2	24.6604	111.33	295.551
2019/8/16 1:07	184	11.2802	25.2	6.90656	43.0906	650	10.2	24.7404	111.49	296.001
2019/8/16 1:08	266	11.3002	25.2	6.90656	42.1991	650	10.2	24.7404	111.65	296.651
2019/8/16 1:08	350	11.3102	25.1	6.90656	42.7935	650	10.2	24.8204	111.81	297.251
2019/8/16 1:09	437	11.3202	25.1	6.90656	43.0906	650	10.2	24.8404	111.969	297.701
2019/8/16 1:10	525	11.3302	25.1	6.90656	43.3878	650	10.2	24.9804	112.132	298.301
2019/8/16 1:10	607	11.3402	25.2	6.90656	42.4963	650	10.2	25.0204	112.291	298.751
2019/8/16 1:11	691	11.3502	25.1	6.90656	42.4963	650	10.2	25.1004	112.451	299.35
2019/8/16 1:11	772	11.3602	25.1	6.90656	43.2392	650	10.2	25.1604	112.611	299.95
2019/8/16 1:12	854	11.3702	25.2	6.90656	42.7935	650	10.2	25.1604	112.771	300.4
2019/8/16 1:13	938	11.3802	25.2	6.90656	41.9019	650	10.2	25.3004	112.933	301.05
2019/8/16 1:13	22	11.3802	25.1	6.90656	42.4963	650	10.2	25.3004	113.088	301.5
2019/8/16 1:14	107	11.3902	25.1	6.90656	43.8336	650	10.2	25.5204	113.248	302.1
2019/8/16 1:14	185	11.4002	25.1	6.90656	42.7935	650	10.2	25.5204	113.408	302.7
2019/8/16 1:15	265	11.4102	25.2	6.90656	41.6048	650	10.2	25.5404	113.568	303.15
2019/8/16 1:16	352	11.4202	25.1	6.90656	41.4562	650	10.2	25.6004	113.728	303.749
2019/8/16 1:16	438	11.4302	25.1	6.90656	41.9019	650	10.2	25.6204	113.888	304.199
2019/8/16 1:17	524	11.4402	25.1	6.90656	42.1991	650	10.2	25.7204	114.05	304.799
2019/8/16 1:17	610	11.4502	25.2	6.90656	41.6048	650	10.2	25.8004	114.21	305.399
2019/8/16 1:18	693	11.4602	25.2	6.90656	41.0104	650	10.2	25.8804	114.369	305.849
2019/8/16 1:19	776	11.4802	25	6.90656	40.416	650	10.2	25.9004	114.529	306.449
2019/8/16 1:19	0	11.4902	25	6.90656	41.6048	650	10.2	25.9804	114.689	306.899
2019/8/16 1:20	582	11.4902	25.1	6.90656	41.6048	650	10.2	25.9804	114.849	307.498

2019/8/16 1:20	337	11.5002	25.2	6.90656	41.4562	650	10.2	26.0804	115.009	308.098
2019/8/16 1:21	330	11.5102	25.2	6.90656	40.416	650	10.2	26.1004	115.169	308.548
2019/8/16 1:22	704	11.5202	25	6.90656	40.1189	650	10.2	26.1604	115.329	309.148
2019/8/16 1:22	781	11.5302	25	6.90656	41.6048	650	10.2	26.3604	115.488	309.598
2019/8/16 1:23	862	11.5402	25	6.90656	41.3076	650	10.2	26.3604	115.648	310.198
2019/8/16 1:23	947	11.5502	25.2	6.90656	40.5646	650	10.2	26.3604	115.81	310.848
2019/8/16 1:24	31	11.5602	25.3	6.90656	39.6731	650	10.2	26.3604	115.966	311.298
2019/8/16 1:25	114	11.5702	25.1	6.90656	39.6731	650	10.2	26.3604	116.126	311.897
2019/8/16 1:25	193	11.5802	25	6.90656	40.5646	650	10.2	26.4804	116.285	312.347
2019/8/16 1:26	277	11.5902	25.1	6.90656	40.5646	650	10.2	26.5004	116.445	312.947
2019/8/16 1:26	360	11.6002	25.2	6.90656	40.2675	650	10.2	26.5804	116.605	313.397
2019/8/16 1:27	442	11.6102	25.2	6.90656	39.5245	650	10.2	26.6604	116.767	313.997
2019/8/16 1:28	523	11.6202	25.1	6.90656	39.0788	650	10.2	26.7404	116.927	314.597
2019/8/16 1:28	602	11.6302	25	6.90656	39.6731	650	10.2	26.8204	117.087	315.047
2019/8/16 1:29	682	11.6402	25.1	6.90656	39.3759	650	10.2	26.8404	117.247	315.646
2019/8/16 1:29	765	11.6502	25.2	6.90656	39.2273	650	10.2	26.9004	117.407	316.096
2019/8/16 1:30	851	11.6602	25.2	6.90656	37.5929	650	10.2	26.9204	117.567	316.696
2019/8/16 1:31	934	11.6702	25	6.90656	38.1872	650	10.2	27.1004	117.726	317.296
2019/8/16 1:31	17	11.6802	25.1	6.90656	38.1872	650	10.2	27.1004	117.884	317.746
2019/8/16 1:32	100	11.6902	25.2	6.90656	38.1872	650	10.2	27.1004	118.044	318.346
2019/8/16 1:32	185	11.7002	25.2	6.90656	37.2957	650	10.2	27.2204	118.204	318.796
2019/8/16 1:33	268	11.7102	25	6.90656	37.2957	650	10.2	27.2804	118.364	319.396
2019/8/16 1:34	353	11.7202	25	6.90656	38.1872	650	10.2	27.3604	118.523	319.995
2019/8/16 1:34	432	11.7302	25.1	6.90656	38.4844	650	10.2	27.3804	118.683	320.445
2019/8/16 1:35	516	11.7402	25.2	6.90656	37.89	650	10.2	27.4404	118.845	321.045
2019/8/16 1:35	598	11.7502	25.2	6.90656	36.9985	650	10.2	27.4604	119.005	321.495
2019/8/16 1:36	680	11.7602	25	6.90656	36.4042	650	10.2	27.4604	119.165	322.095
2019/8/16 1:37	764	11.7702	25	6.90656	37.5929	650	10.2	27.5204	119.325	322.695
2019/8/16 1:37	851	11.7802	25.1	6.90656	36.9985	650	10.2	27.6404	119.485	323.145
2019/8/16 1:38	941	11.7902	25.1	6.90656	36.107	650	10.2	27.7204	119.647	323.744
2019/8/16 1:38	30	11.8002	25.2	6.90656	36.2556	650	10.2	27.7404	119.802	324.194
2019/8/16 1:39	117	11.8102	25.1	6.90656	35.8098	650	10.2	27.9804	119.962	324.794
2019/8/16 1:40	202	11.8202	25.1	6.90656	36.2556	650	10.2	27.9804	120.122	325.294
2019/8/16 1:40	283	11.8302	25.2	6.90656	35.2155	650	10.2	27.9804	120.282	325.844
2019/8/16 1:41	365	11.8402	25.1	6.90656	34.0267	650	10.2	27.9804	120.442	326.444

2019/8/16 1:41	449	11.8502	25	6.90656	34.9183	650	10.2	28.1605	120.604	326.894
2019/8/16 1:42	532	11.8602	25	6.90656	35.5126	650	10.2	28.1605	120.764	327.494
2019/8/16 1:43	805	11.8702	25.1	6.90656	34.9183	650	10.2	28.1605	120.923	328.043
2019/8/16 1:43	891	11.8802	25.2	6.90656	33.7296	650	10.2	28.3205	121.085	328.543
2019/8/16 1:44	980	11.8902	25	6.90656	33.8782	650	10.2	28.3205	121.245	329.143
2019/8/16 1:44	64	11.9002	25.1	6.90656	34.3239	650	10.2	28.3205	121.401	329.593
2019/8/16 1:45	149	11.9102	25.2	6.90656	34.0267	650	10.2	28.3805	121.561	330.193
2019/8/16 1:46	237	11.9202	25.3	6.90656	33.8782	650	10.2	28.5405	121.72	330.643
2019/8/16 1:46	320	11.9302	25.1	6.90656	32.9866	650	10.2	28.5405	121.88	331.243
2019/8/16 1:47	406	11.9402	25.1	6.90656	33.4324	650	10.2	28.6605	122.042	331.842
2019/8/16 1:47	490	11.9502	25.2	6.90656	33.1352	650	10.2	28.7005	122.202	332.342
2019/8/16 1:48	572	11.9602	25.2	6.90656	31.6493	650	10.2	28.8405	122.362	332.942
2019/8/16 1:49	661	11.9702	25.1	6.90656	32.5409	650	10.2	28.8405	122.522	333.392
2019/8/16 1:49	743	11.9802	25.1	6.90656	33.1352	650	10.2	28.9005	122.682	333.992
2019/8/16 1:50	823	11.9902	25.2	6.90656	32.5409	650	10.2	28.9805	122.842	334.592
2019/8/16 1:50	911	12.0002	25.1	6.90656	31.055	650	10.2	29.0205	123.004	335.042
2019/8/16 1:51	995	12.0102	25.1	6.90656	31.3522	650	10.2	29.1005	123.164	335.642
2019/8/16 1:52	77	12.0202	25.1	6.90656	31.3522	650	10.2	29.1805	123.319	336.091
2019/8/16 1:52	160	12.0302	25.2	6.90656	31.3522	650	10.2	29.2205	123.479	336.691
2019/8/16 1:53	242	12.0402	25.3	6.90656	30.4606	650	10.2	29.5405	123.639	337.341
2019/8/16 1:53	329	12.0502	25.1	6.90656	29.5691	650	10.2	29.5405	123.798	337.791
2019/8/16 1:54	414	12.0602	25.1	6.90656	30.1634	650	10.2	29.5405	123.961	338.391
2019/8/16 1:55	503	12.0702	25.2	6.90656	29.8663	650	10.2	29.5405	124.12	338.841
2019/8/16 1:55	595	12.0802	25.3	6.90656	28.9747	650	10.2	29.5605	124.28	339.441
2019/8/16 1:56	679	12.0902	25.1	6.90656	28.3804	650	10.2	29.6805	124.44	340.04
2019/8/16 1:56	761	12.1002	25.1	6.90656	28.9747	650	10.2	29.7405	124.6	340.49
2019/8/16 1:57	846	12.1102	25.1	6.90656	28.9747	650	10.2	29.8405	124.762	341.09
2019/8/16 1:58	931	12.1202	25.2	6.90656	27.4889	650	10.2	29.8405	124.922	341.54
2019/8/16 1:58	14	12.1302	25	6.90656	26.8945	650	10.2	29.8605	125.077	342.14
2019/8/16 1:59	95	12.1402	25	6.90656	28.0832	650	10.2	29.9605	125.237	342.59
2019/8/16 1:59	181	12.1502	25.1	6.90656	27.786	650	10.2	30.0205	125.397	343.19
2019/8/16 2:00	265	12.1602	25.1	6.90656	27.3403	650	10.2	30.0405	125.557	343.74
2019/8/16 2:01	346	12.1702	25.2	6.90656	26.8945	650	10.2	30.1605	125.717	344.189
2019/8/16 2:01	434	12.1802	25.1	6.90656	26.3001	650	10.2	30.1605	125.879	344.789
2019/8/16 2:02	521	12.1902	25.1	6.90656	26.8945	650	10.2	30.2405	126.039	345.239

2019/8/16 2:02	601	12.2002	25.2	6.90656	27.1917	650	10.2	30.2605	126.198	345.839
2019/8/16 2:03	684	12.2102	25.2	6.90656	26.003	650	10.2	30.2605	126.358	346.439
2019/8/16 2:04	776	12.2202	25.1	6.90656	25.4086	650	10.2	30.3005	126.518	346.889
2019/8/16 2:04	863	12.2302	25.1	6.90656	25.7058	650	10.2	30.4205	126.678	347.539
2019/8/16 2:05	946	12.2402	25.1	6.90656	24.8143	650	10.2	30.5005	126.84	348.039
2019/8/16 2:05	31	12.2502	25.2	6.90656	24.5171	650	10.2	30.5205	126.995	348.588
2019/8/16 2:06	114	12.2602	25.1	6.90656	23.3284	650	10.2	30.6605	127.155	349.188
2019/8/16 2:07	198	12.2702	25	6.90656	24.8143	650	10.2	30.7605	127.315	349.638
2019/8/16 2:07	286	12.2802	25.1	6.90656	24.2199	650	10.2	30.7605	127.475	350.238
2019/8/16 2:08	374	12.2902	25.1	6.90656	23.3284	650	10.2	30.8005	127.637	350.688
2019/8/16 2:08	455	12.3002	25.3	6.90656	22.1397	650	10.2	30.8005	127.797	351.288
2019/8/16 2:09	536	12.3102	25.1	6.90656	21.5453	650	10.2	30.9805	127.957	351.888
2019/8/16 2:10	626	12.3202	25.1	6.90656	21.8425	650	10.2	30.9805	128.117	352.387
2019/8/16 2:10	712	12.3302	25.1	6.90656	21.2481	650	10.2	30.9805	128.277	352.987
2019/8/16 2:11	793	12.3402	25.2	6.90656	20.3566	650	10.2	31.0405	128.438	353.437
2019/8/16 2:11	880	12.3502	25	6.90656	20.3566	650	10.2	31.0605	128.601	354.037
2019/8/16 2:12	965	12.3602	25.1	6.90656	21.5453	650	10.2	31.1205	128.761	354.637
2019/8/16 2:13	49	12.3702	25.1	6.90656	20.6538	650	10.2	31.1205	128.917	355.087
2019/8/16 2:13	137	12.3802	25.2	6.90656	20.0594	650	10.2	31.2605	129.077	355.687
2019/8/16 2:14	216	12.3902	25.2	6.90656	17.9792	650	10.2	31.2605	129.238	356.137
2019/8/16 2:14	305	12.4002	25	6.90656	19.1679	650	10.2	31.2605	129.398	356.736
2019/8/16 2:15	392	12.4102	25.2	6.90656	19.4651	650	10.2	31.2805	129.561	357.336
2019/8/16 2:16	473	12.4202	25.2	6.90656	19.4651	650	10.2	31.3405	129.721	357.786
2019/8/16 2:16	558	12.4302	25.2	6.90656	18.2764	650	10.2	31.3605	129.881	358.386
2019/8/16 2:17	640	12.4402	25.1	6.90656	18.5736	650	10.2	31.4805	130.042	358.836
2019/8/16 2:17	732	12.4502	25.1	6.90656	18.2764	650	10.2	31.4805	130.202	359.436
2019/8/16 2:18	817	12.4602	25.2	6.90656	17.3848	650	10.2	31.5005	130.365	360.036
2019/8/16 2:19	904	12.4702	25.2	6.90656	17.0877	650	10.2	31.6405	130.525	360.486
2019/8/16 2:19	986	12.4802	25.1	6.90656	17.0877	650	10.2	31.6605	130.686	361.085
2019/8/16 2:20	64	12.4902	25.1	6.90656	17.0877	650	10.2	31.7005	130.842	361.535
2019/8/16 2:20	150	12.5002	25.3	6.90656	16.0475	650	10.2	31.7405	131.002	362.135
2019/8/16 2:21	234	12.5102	25.1	6.90656	14.7103	650	10.2	31.8005	131.162	362.635
2019/8/16 2:22	315	12.5202	25	6.90656	15.4532	650	10.2	31.8805	131.325	363.235
2019/8/16 2:22	402	12.5302	25.1	6.90656	15.156	650	10.2	31.9205	131.485	363.835
2019/8/16 2:23	486	12.5402	25.2	6.90656	15.156	650	10.2	32.0005	131.646	364.285

2019/8/16 2:23	567	12.5502	25.2	6.90656	13.2244	650	10.2	32.0605	131.806	364.884
2019/8/16 2:24	651	12.5602	25.1	6.90656	13.6701	650	10.2	32.0605	131.967	365.334
2019/8/16 2:25	739	12.5702	25.2	6.90656	13.373	650	10.2	32.1405	132.129	365.934
2019/8/16 2:25	821	12.5802	25.2	6.90656	13.6701	650	10.2	32.1805	132.29	366.534
2019/8/16 2:26	907	12.5902	25.2	6.90656	11.8871	650	10.2	32.2005	132.45	366.984
2019/8/16 2:26	991	12.6002	25	6.90656	12.7786	650	10.2	32.3405	132.611	367.584
2019/8/16 2:27	79	12.6102	25.1	6.90656	13.2244	650	10.2	32.3405	132.766	368.034
2019/8/16 2:28	165	12.6202	25.2	6.90656	12.1842	650	10.2	32.3605	132.927	368.634
2019/8/16 2:28	250	12.6302	25.2	6.90656	10.6984	650	10.2	32.4606	133.087	369.233
2019/8/16 2:29	333	12.6402	25	6.90656	9.80684	650	10.2	32.4606	133.25	369.683
2019/8/16 2:29	416	12.6502	25	6.90656	10.6984	650	10.2	32.4806	133.41	370.283
2019/8/16 2:30	498	12.6602	25.1	6.90656	10.9955	650	10.2	32.5406	133.571	370.783
2019/8/16 2:31	579	12.6702	25.2	6.90656	9.80684	650	10.2	32.6206	133.731	371.383
2019/8/16 2:31	668	12.6802	25.1	6.90656	8.46954	650	10.2	32.6406	133.891	371.983
2019/8/16 2:32	749	12.6902	25.1	6.90656	8.46954	650	10.2	32.7806	134.052	372.433
2019/8/16 2:32	832	12.7002	25.1	6.90656	8.61813	650	10.2	32.7806	134.215	373.032
2019/8/16 2:33	915	12.7102	25.2	6.90656	7.13224	650	10.2	32.8206	134.375	373.482
2019/8/16 2:34	0	12.7202	25.1	6.90656	5.64636	650	10.2	32.9006	134.535	374.082
2019/8/16 2:34	86	12.7302	25.1	6.90656	6.09212	650	10.2	32.9206	134.691	374.582
2019/8/16 2:35	176	12.7402	25.2	6.90656	6.24071	650	10.2	33.1006	134.852	375.132
2019/8/16 2:35	258	12.7502	25.3	6.90656	4.90342	650	10.2	33.1006	135.012	375.732
2019/8/16 2:36	344	12.7602	25.1	6.90656	4.60624	650	10.2	33.1406	135.175	376.232
2019/8/16 2:37	429	12.7702	25	6.90656	5.79495	650	10.2	33.2006	135.335	376.832
2019/8/16 2:37	517	12.7802	25.1	6.90656	5.79495	650	10.2	33.3206	135.495	377.381
2019/8/16 2:38	596	12.7902	25.2	6.90656	4.45765	650	10.2	33.3206	135.656	377.881
2019/8/16 2:38	681	12.8002	25.2	6.90656	2.526	650	10.2	33.4006	135.816	378.481
2019/8/16 2:39	763	12.8102	25.1	6.90656	2.97177	650	10.2	33.4006	135.979	378.931
2019/8/16 2:40	850	12.8202	25.1	6.90656	3.56612	650	10.2	33.4406	136.139	379.531
2019/8/16 2:40	939	12.8302	25.2	6.90656	2.67459	650	10.2	33.4606	136.3	380.131
2019/8/16 2:41	22	12.8402	25.1	6.90656	1.78306	650	10.2	33.5606	136.456	380.581
2019/8/16 2:41	104	12.8502	25.1	6.90656	2.08024	650	10.2	33.5806	136.616	381.18
2019/8/16 2:42	190	12.8602	25.2	6.90656	1.78306	650	10.2	33.6406	136.776	381.63
2019/8/16 2:43	270	12.8702	25.2	6.90656	1.18871	650	10.2	33.7406	136.939	382.23
2019/8/16 2:43	359	12.8802	25.1	6.90656	1.3373	650	10.2	33.7406	137.1	382.68
2019/8/16 2:44	444	12.8902	25.1	6.90656	1.48588	650	10.2	33.7806	137.26	383.28

2019/8/16 2:44	524	12.9002	25.2	6.90656	0.89153	650	10.2	33.9206	137.42	383.88
2019/8/16 2:45	607	12.9102	25.2	6.90656	0.89153	650	10.2	34.2806	137.581	384.33
2019/8/16 2:46	692	12.9202	25.1	6.90656	0.594354	650	10.2	34.2806	137.741	384.93
2019/8/16 2:46	774	12.9302	25.1	6.90656	0.594354	650	10.2	34.2806	137.904	385.379
2019/8/16 2:47	866	12.9402	25.3	6.90656	0.445765	650	10.2	34.2806	138.064	385.979
2019/8/16 2:47	947	12.9502	25.1	6.90656	0.297177	650	10.2	34.2806	138.225	386.579
2019/8/16 2:48	29	12.9602	25.1	6.90656	0.297177	650	10.2	34.3006	138.38	387.029
2019/8/16 2:49	110	12.9702	25.2	6.90656	0.297177	650	10.2	34.3406	138.541	387.629
2019/8/16 2:49	200	12.9802	25.1	6.90656	0	650	10.2	34.3606	138.704	388.079
2019/8/16 2:50	279	12.9902	25.1	6.90656	0	650	10.2	34.4406	138.864	388.729
2019/8/16 2:50	364	13.0002	25.2	6.90656	0	650	10.2	34.4606	139.024	389.328
2019/8/16 2:51	448	13.0102	25.3	6.90656	0	650	10.2	34.5006	139.185	389.778
2019/8/16 2:52	534	13.0202	25.1	6.90656	0	650	10.2	34.5406	139.345	390.378
2019/8/16 2:52	619	13.0302	25.1	6.90656	0	650	10.2	34.5606	139.506	390.828
2019/8/16 2:53	705	13.0402	25.2	6.90656	0	650	10.2	34.6206	139.668	391.428
2019/8/16 2:53	789	13.0502	25.2	6.90656	0	650	10.2	34.7406	139.829	392.028
2019/8/16 2:54	873	13.0602	25.1	6.90656	0	650	10.2	34.7806	139.989	392.478
2019/8/16 2:55	963	13.0702	25.1	6.90656	0	650	10.2	34.8606	140.149	393.078
2019/8/16 2:55	50	13.0802	25.2	6.90656	0	650	10.2	34.9406	140.305	393.527
2019/8/16 2:56	133	13.0902	25.2	6.90656	0	650	10.2	35.0006	140.466	394.127
2019/8/16 2:56	222	13.1002	25.1	6.90656	0	650	10.2	35.0406	140.628	394.677
2019/8/16 2:57	305	13.1102	25.1	6.90656	0	650	10.2	35.0606	140.789	395.177
2019/8/16 2:58	389	13.1202	25.2	6.90656	0	650	10.2	35.0606	140.949	395.777
2019/8/16 2:58	476	13.1302	25.2	6.90656	0	650	10.2	35.1206	141.11	396.227
2019/8/16 2:59	567	13.1402	25.1	6.90656	0	650	10.2	35.1606	141.27	396.877
2019/8/16 2:59	650	13.1502	25	6.90656	0	650	10.2	35.2206	141.43	397.376
2019/8/16 3:00	738	13.1602	25.2	6.90656	0	650	10.2	35.2406	141.593	397.926
2019/8/16 3:01	819	13.1702	25.2	6.90656	0	650	10.2	35.3806	141.753	398.526
2019/8/16 3:01	903	13.1802	25	6.90656	0	650	10.2	35.3806	141.914	398.976
2019/8/16 3:02	986	13.1902	25.1	6.90656	0	650	10.2	35.4406	142.074	399.576
2019/8/16 3:02	68	13.2002	25.2	6.90656	0	650	10.2	35.4606	142.23	400.026
2019/8/16 3:03	0	13.2102	25.2	6.90656	0	700	10.2	35.4606	142.39	400.626
2019/8/16 3:04	122	13.2202	25.1	6.90656	0.297177	700	10.2	35.5806	142.553	401.226
2019/8/16 3:04	324	13.2302	25.1	6.90656	1.63447	700	10.2	35.5806	142.714	401.226
2019/8/16 3:05	405	13.2402	25.2	6.90656	1.18871	700	10.2	35.5806	142.874	401.226

2019/8/16 3:05	492	13.2502	25.1	6.90656	0.89153	700	10.2	35.6006	143.034	401.226
2019/8/16 3:06	577	13.2602	25.1	6.90656	4.30906	700	10.2	35.6206	143.195	401.226
2019/8/16 3:07	666	13.2702	25.2	6.90656	5.49777	700	10.2	35.6806	143.355	401.226
2019/8/16 3:07	754	13.2802	25.2	6.90656	5.49777	700	10.2	35.6806	143.518	401.226
2019/8/16 3:08	840	13.2902	25	6.90656	6.83507	700	10.2	35.6806	143.678	401.226
2019/8/16 3:08	922	13.3002	25	6.90656	9.80684	700	10.2	35.6806	143.839	401.226
2019/8/16 3:09	9	13.3102	25.1	6.90656	10.6984	700	10.2	35.6806	143.994	401.226
2019/8/16 3:10	89	13.3202	25.3	6.90656	10.104	700	10.2	35.7006	144.155	401.226
2019/8/16 3:10	169	13.3302	25.1	6.90656	10.4012	700	10.2	35.7006	144.315	401.226
2019/8/16 3:11	259	13.3402	25.1	6.90656	11.5899	700	10.2	35.7006	144.478	401.226
2019/8/16 3:11	345	13.3502	25.2	6.90656	11.4413	700	10.2	35.7606	144.638	401.226
2019/8/16 3:12	430	13.3602	25.2	6.90656	9.50966	700	10.2	35.8006	144.799	401.226
2019/8/16 3:13	513	13.3702	25.1	6.90656	10.6984	700	10.2	35.8006	144.959	401.226
2019/8/16 3:13	596	13.3802	25.1	6.90656	11.5899	700	10.2	35.8206	145.12	401.226
2019/8/16 3:14	680	13.3902	25.2	6.90656	10.6984	700	10.2	35.8606	145.282	401.226
2019/8/16 3:14	766	13.4002	25.1	6.90656	9.50966	700	10.2	35.9006	145.443	401.226
2019/8/16 3:15	849	13.4102	25.1	6.90656	10.104	700	10.2	35.9206	145.603	401.226
2019/8/16 3:16	938	13.4202	25.1	6.90656	9.80684	700	10.2	36.0006	145.763	401.226
2019/8/16 3:16	20	13.4302	25.2	6.90656	9.50966	700	10.2	36.0606	145.919	401.226
2019/8/16 3:17	99	13.4402	25	6.90656	10.104	700	10.2	36.0606	146.08	401.226
2019/8/16 3:17	180	13.4502	25.1	6.90656	10.4012	700	10.2	36.0806	146.242	401.226
2019/8/16 3:18	263	13.4602	25.2	6.90656	9.50966	700	10.2	36.1206	146.403	401.226
2019/8/16 3:19	348	13.4702	25.2	6.90656	8.9153	750	10.2	36.1206	146.563	401.226
2019/8/16 3:19	433	13.4802	25	6.90656	16.4933	750	10.2	36.1206	146.724	401.226
2019/8/16 3:20	530	13.4902	25.1	6.90656	21.6939	750	10.2	36.1206	146.884	401.226
2019/8/16 3:20	610	13.5002	25.3	6.90656	22.2883	750	10.2	36.1606	147.044	401.226
2019/8/16 3:21	698	13.5102	25.1	6.90656	21.5453	750	10.2	36.1606	147.207	401.226
2019/8/16 3:22	783	13.5202	25	6.90656	23.1798	750	10.2	36.1606	147.367	401.226
2019/8/16 3:22	867	13.5302	25.1	6.90656	22.5854	750	10.2	36.3006	147.528	401.226
2019/8/16 3:23	958	13.5402	25.2	6.90656	21.0996	750	10.2	36.3006	147.688	401.226
2019/8/16 3:23	38	13.5502	25.1	6.90656	21.3967	750	10.2	36.3006	147.844	401.226
2019/8/16 3:24	119	13.5602	25.1	6.90656	21.9911	750	10.2	36.3206	148.005	401.226
2019/8/16 3:25	200	13.5702	25.2	6.90656	20.5052	750	10.2	36.4206	148.167	401.226
2019/8/16 3:25	286	13.5802	25.1	6.90656	19.3165	750	10.2	36.4206	148.328	401.226
2019/8/16 3:26	370	13.5902	25.1	6.90656	19.9108	750	10.2	36.4206	148.488	401.226

2019/8/16 3:26	458	13.6002	25.2	6.90656	19.9108	750	10.2	36.4406	148.648	401.226
2019/8/16 3:27	545	13.6102	25.2	6.90656	18.7221	750	10.2	36.4806	148.809	401.226
2019/8/16 3:28	630	13.6202	25.1	6.90656	18.425	750	10.2	36.4806	148.971	401.226
2019/8/16 3:28	714	13.6302	25.1	6.90656	19.7623	750	10.2	36.5006	149.132	401.226
2019/8/16 3:29	797	13.6402	25.2	6.90656	18.425	750	10.2	36.6006	149.292	401.226
2019/8/16 3:29	885	13.6502	25.2	6.90656	17.5334	750	10.2	36.6006	149.453	401.226
2019/8/16 3:30	976	13.6602	25	6.90656	18.7221	750	10.2	36.6006	149.613	401.226
2019/8/16 3:31	56	13.6702	25.1	6.90656	19.3165	750	10.2	36.6406	149.769	401.226
2019/8/16 3:31	142	13.6802	25.2	6.90656	17.5334	750	10.2	36.6606	149.932	401.226
2019/8/16 3:32	225	13.6902	25.1	6.90656	17.2363	750	10.2	36.7006	150.092	401.226
2019/8/16 3:32	308	13.7002	25.1	6.90656	17.8306	750	10.2	36.7807	150.252	401.226
2019/8/16 3:33	394	13.7102	25.2	6.90656	17.2363	750	10.2	36.7807	150.413	401.226
2019/8/16 3:34	476	13.7202	25.1	6.90656	16.4933	750	10.2	36.8007	150.573	401.226
2019/8/16 3:34	560	13.7302	25	6.90656	17.5334	750	10.2	36.8007	150.734	401.226
2019/8/16 3:35	645	13.7402	25.1	6.90656	17.5334	750	10.2	36.8407	150.896	401.226
2019/8/16 3:35	731	13.7502	25.2	6.90656	16.4933	750	10.2	36.8407	151.057	401.226
2019/8/16 3:36	814	13.7602	25.1	6.90656	15.6018	750	10.2	36.8807	151.217	401.226
2019/8/16 3:37	897	13.7702	25.1	6.90656	16.9391	750	10.2	36.8807	151.377	401.226
2019/8/16 3:37	983	13.7802	25.2	6.89618	16.4933	750	10.2	36.9407	151.538	401.226
2019/8/16 3:38	64	13.7902	25.1	6.90656	14.7103	750	10.2	37.0607	151.694	401.226
2019/8/16 3:38	147	13.8002	25.1	6.90656	15.0074	750	10.2	37.0607	151.856	401.226
2019/8/16 3:39	234	13.8102	25.1	6.90656	14.1159	750	10.2	37.0607	152.017	401.226
2019/8/16 3:40	315	13.8202	25.1	6.90656	12.9272	750	10.2	37.0807	152.177	401.226
2019/8/16 3:40	404	13.8302	25.1	6.90656	13.2244	750	10.2	37.2607	152.338	401.226
2019/8/16 3:41	484	13.8402	25.2	6.90656	12.63	750	10.2	37.2607	152.498	401.226
2019/8/16 3:41	568	13.8502	25.2	6.90656	11.7385	750	10.2	37.2607	152.658	401.226
2019/8/16 3:42	653	13.8602	25.1	6.90656	12.63	750	10.2	37.2607	152.821	401.226
2019/8/16 3:43	739	13.8702	25.1	6.90656	12.0357	750	10.2	37.2607	152.981	401.226
2019/8/16 3:43	824	13.8802	25.2	6.90656	10.9955	750	10.2	37.3007	153.142	401.226
2019/8/16 3:44	910	13.8902	25.1	6.90656	12.0357	750	10.2	37.3007	153.302	401.226
2019/8/16 3:44	994	13.9002	25.2	6.90656	12.0357	750	10.2	37.3007	153.463	401.226
2019/8/16 3:45	71	13.9102	25.2	6.90656	10.847	750	10.2	37.3607	153.619	401.226
2019/8/16 3:46	157	13.9202	25.1	6.90656	10.2526	750	10.2	37.4007	153.781	401.226
2019/8/16 3:46	236	13.9302	25.2	6.90656	9.95542	750	10.2	37.4007	153.942	401.226
2019/8/16 3:47	320	13.9402	25.2	6.90656	9.36107	750	10.2	37.4007	154.102	401.226

2019/8/16 3:47	400	13.9502	25.1	6.90656	9.95542	750	10.2	37.4207	154.262	401.226
2019/8/16 3:48	488	13.9602	25.1	6.90656	10.5498	750	10.2	37.4207	154.423	401.226
2019/8/16 3:49	567	13.9702	25.3	6.90656	8.76672	750	10.2	37.4607	154.583	401.226
2019/8/16 3:49	647	13.9802	25.1	6.90656	7.57801	750	10.2	37.5607	154.746	401.226
2019/8/16 3:50	735	13.9902	25.1	6.90656	8.46954	750	10.2	37.5607	154.906	401.226
2019/8/16 3:50	818	14.0002	25.2	6.90656	8.02377	750	10.2	37.6007	155.067	401.226
2019/8/16 3:51	907	14.0102	25.2	6.90656	6.24071	750	10.2	37.6007	155.227	401.226
2019/8/16 3:52	993	14.0202	25	6.90656	5.34918	750	10.2	37.6007	155.387	401.226
2019/8/16 3:52	77	14.0302	25	6.90656	6.24071	750	10.2	37.6807	155.546	401.226
2019/8/16 3:53	158	14.0402	25.2	6.90656	5.64636	750	10.2	37.6807	155.706	401.226
2019/8/16 3:53	244	14.0502	25.2	6.90656	3.71471	750	10.2	37.8007	155.832	401.226
2019/8/16 3:54	328	14.0602	25.1	6.90656	3.71471	750	10.2	37.8007	155.952	401.226
2019/8/16 3:55	409	14.0702	25.1	6.90656	4.01189	750	10.2	37.8007	156.072	401.226
2019/8/16 3:55	492	14.0802	25.3	6.90656	3.12036	750	10.2	37.8207	156.172	401.226
2019/8/16 3:56	581	14.0902	25.1	6.90656	2.526	750	10.2	37.8407	156.172	401.226
2019/8/16 3:56	674	14.1002	25	6.90656	4.60624	750	10.2	37.8407	156.172	401.226
2019/8/16 3:57	560	14.1102	25.1	6.90656	6.09212	750	10.2	37.8407	156.172	401.226
2019/8/16 3:58	830	14.1202	25.2	6.90656	6.98366	750	10.2	37.8807	156.172	401.226
2019/8/16 3:58	919	14.1302	25.1	6.90656	9.06389	750	10.2	37.8807	156.172	401.226
2019/8/16 3:59	4	14.1402	25.1	6.90656	11.2927	750	10.2	37.8807	156.172	401.226
2019/8/16 3:59	87	14.1502	25.2	6.90656	11.7385	750	10.2	37.8807	156.172	401.226
2019/8/16 4:00	168	14.1602	25.2	6.90656	12.0357	750	10.2	37.9007	156.172	401.226
2019/8/16 4:01	252	14.1702	25.1	6.90656	14.1159	750	10.2	37.9007	156.172	401.226
2019/8/16 4:01	337	14.1802	25.2	6.90656	16.0475	750	10.2	37.9007	156.172	401.226
2019/8/16 4:02	420	14.1902	25.2	6.90656	16.7905	750	10.2	37.9007	156.172	401.226
2019/8/16 4:02	501	14.2002	25.1	6.90656	16.3447	750	10.2	37.9007	156.172	401.226
2019/8/16 4:03	583	14.2102	25	6.90656	18.2764	750	10.2	37.9007	156.172	401.226
2019/8/16 4:04	668	14.2202	25.2	6.90656	18.5736	750	10.2	37.9007	156.172	401.226
2019/8/16 4:04	755	14.2302	25.2	6.90656	17.682	750	10.2	37.9207	156.172	401.226
2019/8/16 4:05	836	14.2402	25	6.90656	17.8306	750	10.2	37.9407	156.172	401.226
2019/8/16 4:05	921	14.2502	25	6.90656	19.1679	750	10.2	37.9407	156.172	401.226
2019/8/16 4:06	9	14.2602	25.2	6.90656	18.8707	750	10.2	37.9407	156.172	401.226
2019/8/16 4:07	347	14.2702	25.2	6.90656	18.1278	750	10.2	37.9407	156.172	401.226
2019/8/16 4:07	428	14.2802	25	6.90656	17.9792	750	10.2	37.9607	156.172	401.226
2019/8/16 4:08	511	14.2902	25.1	6.90656	19.7623	750	10.2	37.9607	156.172	401.226

2019/8/16 4:08	596	14.3002	25.2	6.90656	19.1679	750	10.2	38.0007	156.172	401.226
2019/8/16 4:09	685	14.3102	25.2	6.90656	17.9792	750	10.2	38.0007	156.172	401.226
2019/8/16 4:10	767	14.3202	25	6.90656	18.5736	750	10.2	38.0007	156.172	401.226
2019/8/16 4:10	848	14.3302	25.1	6.90656	18.8707	750	10.2	38.0007	156.172	401.226
2019/8/16 4:11	928	14.3402	25.2	6.90656	18.8707	750	10.2	38.0007	156.172	401.226
2019/8/16 4:11	11	14.3502	25.1	6.90656	17.682	750	10.2	38.0207	156.172	401.226
2019/8/16 4:12	94	14.3602	25.1	6.90656	18.7221	750	10.2	38.0207	156.172	401.226
2019/8/16 4:13	178	14.3702	25.1	6.90656	17.9792	750	10.2	38.0207	156.172	401.226
2019/8/16 4:13	261	14.3802	25.3	6.90656	16.3447	750	10.2	38.0207	156.172	401.226
2019/8/16 4:14	347	14.3902	25.1	6.90656	16.1961	750	10.2	38.0207	156.172	401.226
2019/8/16 4:14	435	14.4002	25.1	6.90656	16.0475	750	10.2	38.0207	156.172	401.226
2019/8/16 4:15	521	14.4102	25.3	6.90656	15.156	750	10.2	38.0607	156.172	401.226
2019/8/16 4:16	607	14.4202	25.1	6.90656	14.2645	750	10.2	38.0607	156.172	401.226
2019/8/16 4:16	696	14.4302	25.1	6.90656	13.6701	750	10.2	38.1007	156.172	401.226
2019/8/16 4:17	779	14.4402	25.2	6.90656	12.63	750	10.2	38.1407	156.172	401.226
2019/8/16 4:17	861	14.4502	25.1	6.90656	11.1441	750	10.2	38.1607	156.172	401.226
2019/8/16 4:18	947	14.4602	25	6.90656	11.8871	750	10.2	38.1607	156.172	401.226
2019/8/16 4:19	26	14.4702	25.2	6.90656	11.8871	750	10.2	38.1607	156.172	401.226
2019/8/16 4:19	111	14.4802	25.2	6.90656	9.50966	750	10.2	38.1607	156.172	401.226
2019/8/16 4:20	193	14.4902	25	6.90656	8.46954	750	10.2	38.2407	156.172	401.226
2019/8/16 4:20	276	14.5002	25.1	6.90656	9.21248	750	10.2	38.2407	156.172	401.226
2019/8/16 4:21	369	14.5102	25.2	6.90656	8.46954	750	10.2	38.2407	156.172	401.226
2019/8/16 4:22	454	14.5202	25.1	6.90656	6.83507	750	10.2	38.2407	156.172	401.226
2019/8/16 4:22	539	14.5302	25.1	6.90656	7.42942	750	10.2	38.2407	156.172	401.226
2019/8/16 4:23	625	14.5402	25.2	6.90656	6.3893	750	10.2	38.2607	156.172	401.226
2019/8/16 4:23	710	14.5502	25.2	6.90656	5.05201	750	10.2	38.2807	156.172	401.226
2019/8/16 4:24	794	14.5602	25.1	6.90656	5.05201	750	10.2	38.3207	156.172	401.226
2019/8/16 4:25	883	14.5702	25.2	6.90656	4.75483	750	10.2	38.3207	156.172	401.226
2019/8/16 4:25	968	14.5802	25.2	6.90656	3.56612	750	10.2	38.3207	156.172	401.226
2019/8/16 4:26	52	14.5902	25.1	6.90656	3.26894	750	10.2	38.3607	156.172	401.226
2019/8/16 4:26	139	14.6002	25.1	6.90656	3.26894	750	10.2	38.3607	156.172	401.226
2019/8/16 4:27	222	14.6102	25.2	6.90656	2.22883	750	10.2	38.3607	156.172	401.226
2019/8/16 4:28	306	14.6202	25.1	6.90656	1.48588	750	10.2	38.3807	156.172	401.226
2019/8/16 4:28	393	14.6302	25.1	6.90656	1.78306	750	10.2	38.4007	156.172	401.226
2019/8/16 4:29	476	14.6402	25.2	6.90656	0.742942	750	10.2	38.4007	156.172	401.226

2019/8/16 4:29	560	14.6502	25.2	6.90656	0.297177	750	10.2	38.4407	156.172	401.226
2019/8/16 4:30	645	14.6602	25.1	6.90656	0	750	10.2	38.5207	156.172	401.226
2019/8/16 4:31	692	14.6702	25.2	6.90656	0.742942	800	10.2	38.8407	156.172	401.226
2019/8/16 4:31	770	14.6802	25.2	6.90656	6.24071	800	10.2	38.8407	156.172	401.226
2019/8/16 4:32	860	14.6902	25.1	6.90656	9.36107	800	10.2	38.8407	156.172	401.226
2019/8/16 4:32	944	14.7002	25.2	6.90656	10.2526	800	10.2	38.8407	156.172	401.226
2019/8/16 4:33	66	14.7102	25.3	6.90656	9.95542	800	10.2	38.8407	156.172	401.226
2019/8/16 4:34	147	14.7202	25	6.90656	10.2526	800	10.2	38.8407	156.172	401.226
2019/8/16 4:34	228	14.7302	25.1	6.90656	10.847	800	10.2	38.8407	156.172	401.226
2019/8/16 4:35	306	14.7402	25.2	6.90656	9.95542	800	10.2	38.8407	156.172	401.226
2019/8/16 4:35	390	14.7502	25.2	6.90656	9.06389	800	10.2	38.8407	156.172	401.226
2019/8/16 4:36	477	14.7602	25.1	6.90656	9.80684	800	10.2	38.8407	156.172	401.226
2019/8/16 4:37	570	14.7702	25.2	6.90656	9.65825	800	10.2	38.8407	156.172	401.226
2019/8/16 4:37	650	14.7802	25.2	6.90656	8.76672	800	10.2	38.8407	156.172	401.226
2019/8/16 4:38	732	14.7902	25.1	6.90656	9.36107	800	10.2	38.8807	156.172	401.226
2019/8/16 4:38	817	14.8002	25.2	6.90656	9.06389	800	10.2	38.8807	156.172	401.226
2019/8/16 4:39	897	14.8102	25.2	6.90656	8.17236	800	10.2	38.9207	156.172	401.226
2019/8/16 4:40	985	14.8202	25	6.90656	9.06389	800	10.2	38.9607	156.172	401.226
2019/8/16 4:40	69	14.8302	25.2	6.90656	9.36107	800	10.2	39.0007	156.172	401.226
2019/8/16 4:41	151	14.8402	25.2	6.90656	7.87519	800	10.2	39.0207	156.172	401.226
2019/8/16 4:41	233	14.8502	25.1	6.90656	7.13224	800	10.2	39.0607	156.172	401.226
2019/8/16 4:42	320	14.8602	25.1	6.90656	8.02377	800	10.2	39.0607	156.172	401.226
2019/8/16 4:43	405	14.8702	25.2	6.90656	6.53789	800	10.2	39.0607	156.172	401.226
2019/8/16 4:43	489	14.8802	25.1	6.90656	5.20059	800	10.2	39.0607	156.172	401.226
2019/8/16 4:44	576	14.8902	25.1	6.90656	5.49777	800	10.2	39.0607	156.172	401.226
2019/8/16 4:44	666	14.9002	25.3	6.90656	4.60624	800	10.2	39.0807	156.172	401.226
2019/8/16 4:45	752	14.9102	25.1	6.90656	4.30906	800	10.2	39.0807	156.172	401.226
2019/8/16 4:46	837	14.9202	25.1	6.90656	4.90342	800	10.2	39.0807	156.172	401.226
2019/8/16 4:46	928	14.9302	25.3	6.90656	4.60624	800	10.2	39.1407	156.172	401.226
2019/8/16 4:47	13	14.9402	25.1	6.90656	4.30906	800	10.2	39.1407	156.172	401.226
2019/8/16 4:47	97	14.9502	25.2	6.90656	4.90342	800	10.2	39.1407	156.172	401.226
2019/8/16 4:48	186	14.9602	25.2	6.90656	4.01189	800	10.2	39.1407	156.172	401.226
2019/8/16 4:49	273	14.9702	25	6.90656	4.90342	800	10.2	39.1407	156.172	401.226
2019/8/16 4:49	363	14.9802	25.1	6.90656	4.60624	800	10.2	39.1407	156.172	401.226
2019/8/16 4:50	447	14.9902	25.2	6.90656	3.26894	800	10.2	39.1407	156.172	401.226

2019/8/16 4:50	528	15.0002	25.1	6.90656	3.41753	800	10.2	39.1407	156.172	401.226
2019/8/16 4:51	613	15.0102	25.2	6.90656	3.71471	800	10.2	39.1407	156.172	401.226
2019/8/16 4:52	693	15.0202	25.1	6.90656	3.41753	800	10.2	39.1407	156.172	401.226
2019/8/16 4:52	779	15.0302	25.1	6.90656	3.41753	800	10.2	39.1407	156.172	401.226
2019/8/16 4:53	863	15.0402	25.1	6.90656	2.82318	800	10.2	39.1607	156.172	401.226
2019/8/16 4:53	953	15.0502	25.1	6.90656	3.26894	800	10.2	39.1607	156.172	401.226
2019/8/16 4:54	38	15.0602	25.3	6.90656	4.30906	800	10.2	39.1607	156.172	401.226
2019/8/16 4:55	121	15.0702	25.1	6.90656	5.34918	800	10.2	39.1607	156.172	401.226
2019/8/16 4:55	207	15.0802	25.1	6.90656	7.57801	800	10.2	39.1607	156.172	401.226
2019/8/16 4:56	289	15.0903	25.2	6.90656	7.7266	800	10.2	39.1607	156.172	401.226
2019/8/16 4:56	381	15.1003	25.2	6.90656	6.98366	800	10.2	39.1607	156.172	401.226
2019/8/16 4:57	460	15.1103	25	6.90656	25.5572	800	10.2	39.1607	156.172	401.226
2019/8/16 4:58	541	15.1203	25.1	6.90656	35.9584	800	10.2	39.1607	156.172	401.226
2019/8/16 4:58	627	15.1303	25.2	6.91694	38.3358	800	10.2	39.1607	156.172	401.226
2019/8/16 4:59	713	15.1403	25.1	6.91694	38.7816	800	10.2	39.1607	156.172	401.226
2019/8/16 4:59	795	15.1503	25.1	6.92733	36.9985	800	10.2	39.1607	156.275	401.226
2019/8/16 5:00	886	15.1603	25.2	6.92733	24.5171	800	10.2	39.1607	156.382	401.226
2019/8/16 5:01	967	15.1703	25.2	6.93771	23.9227	800	10.2	39.1607	156.483	401.226
2019/8/16 5:01	56	15.1803	25.1	6.93771	24.8143	800	10.2	39.1607	156.581	401.226
2019/8/16 5:02	142	15.1903	25.1	6.93771	25.8544	800	10.2	39.1607	156.681	401.226
2019/8/16 5:02	229	15.2003	25.2	6.93771	27.9346	800	10.2	39.1607	156.781	401.226
2019/8/16 5:03	311	15.2103	25	6.93771	27.1917	800	10.22	39.1607	156.88	401.226
2019/8/16 5:04	401	15.2203	25	6.93771	28.8262	800	10.34	39.1607	156.982	401.226
2019/8/16 5:04	487	15.2303	25.1	6.93771	29.4205	800	10.34	39.1607	157.082	401.226
2019/8/16 5:05	570	15.2403	25.2	6.93771	27.9346	800	10.34	39.1607	157.182	401.226
2019/8/16 5:05	832	15.2503	25.1	6.94809	28.529	800	10.5001	39.1607	157.282	401.226
2019/8/16 5:06	917	15.2603	25.1	6.92733	29.5691	800	10.5001	39.1607	157.383	401.226
2019/8/16 5:07	2	15.2703	25.2	6.93771	29.8663	800	10.5001	39.1607	157.483	401.226
2019/8/16 5:07	85	15.2803	25.1	6.93771	30.7578	800	10.5001	39.1607	157.583	401.226
2019/8/16 5:08	171	15.2903	25	6.93771	30.4606	800	10.5001	39.1607	157.683	401.226
2019/8/16 5:08	260	15.3003	25.2	6.93771	29.5691	800	10.5001	39.1607	157.783	401.226
2019/8/16 5:09	346	15.3103	25.2	6.93771	27.9346	800	10.5001	39.1607	157.883	401.226
2019/8/16 5:10	430	15.3203	25.1	6.93771	28.529	800	10.5001	39.1607	157.984	401.226
2019/8/16 5:10	517	15.3303	25.1	6.93771	28.6776	800	10.5001	39.1607	158.084	401.226
2019/8/16 5:11	601	15.3403	25.2	6.93771	28.3804	800	10.5001	39.1607	158.184	401.226

2019/8/16 5:11	685	15.3503	25.1	6.93771	29.8663	800	10.6001	39.1607	158.284	401.226
2019/8/16 5:12	770	15.3603	25.1	6.92733	30.0149	800	10.6001	39.1607	158.384	401.226
2019/8/16 5:13	855	15.3703	25.3	6.93771	28.9747	800	10.6001	39.1607	158.484	401.226
2019/8/16 5:13	942	15.3803	25.1	6.93771	28.529	800	10.6001	39.1607	158.586	401.226
2019/8/16 5:14	23	15.3903	25.2	6.93771	28.0832	800	10.6001	39.1607	158.683	401.226
2019/8/16 5:14	110	15.4003	25.3	6.93771	29.5691	800	10.6001	39.1607	158.783	401.226
2019/8/16 5:15	191	15.4103	25.1	6.93771	29.1233	800	10.6001	39.1607	158.883	401.226
2019/8/16 5:16	278	15.4203	25.1	6.93771	29.4205	800	10.6001	39.1607	158.983	401.226
2019/8/16 5:16	367	15.4303	25.2	6.93771	29.2719	800	10.6001	39.1607	159.083	401.226
2019/8/16 5:17	447	15.4403	25.1	6.93771	28.3804	800	10.6601	39.1607	159.184	401.226
2019/8/16 5:17	531	15.4503	25.2	6.93771	27.1917	800	10.6601	39.1607	159.284	401.226
2019/8/16 5:18	620	15.4603	25.2	6.94809	25.5572	800	10.6601	39.1607	159.384	401.226
2019/8/16 5:19	708	15.4703	25.1	6.92733	28.6776	800	10.7601	39.1607	159.484	401.226
2019/8/16 5:19	791	15.4803	25.2	6.93771	28.3804	800	10.7601	39.1607	159.584	401.226
2019/8/16 5:20	878	15.4903	25.2	6.92733	28.529	800	10.7601	39.1607	159.685	401.226
2019/8/16 5:20	960	15.5003	25.1	6.93771	27.9346	800	10.7601	39.1607	159.785	401.226
2019/8/16 5:21	1	15.5103	25.3	6.93771	26.4487	800	10.7601	39.1607	159.883	401.226
2019/8/16 5:22	85	15.5203	25.1	6.93771	25.8544	800	10.7601	39.1607	159.983	401.226
2019/8/16 5:22	169	15.5303	25.1	6.94809	25.7058	800	10.7801	39.1607	160.082	401.226
2019/8/16 5:23	293	15.5403	25.2	6.93771	24.6657	800	10.7801	39.1607	160.182	401.226
2019/8/16 5:23	379	15.5503	25.1	6.93771	26.4487	800	10.8401	39.1607	160.284	401.226
2019/8/16 5:24	466	15.5603	25.1	6.93771	26.1516	800	10.8401	39.1607	160.384	401.226
2019/8/16 5:25	546	15.5703	25.2	6.93771	24.9629	800	10.8401	39.1607	160.484	401.226
2019/8/16 5:25	633	15.5803	25.1	6.93771	24.5171	800	10.9001	39.1607	160.584	401.226
2019/8/16 5:26	716	15.5903	25.1	6.93771	24.2199	800	10.9001	39.1607	160.684	401.226
2019/8/16 5:26	794	15.6003	25.2	6.93771	22.8826	800	10.9001	39.1607	160.785	401.226
2019/8/16 5:27	884	15.6103	25.1	6.93771	22.5854	800	10.9001	39.1607	160.885	401.226
2019/8/16 5:28	969	15.6203	25.1	6.93771	24.8143	800	10.9201	39.1607	160.985	401.226
2019/8/16 5:28	55	15.6303	25.2	6.93771	24.3685	800	11.0201	39.1607	161.082	401.226
2019/8/16 5:29	146	15.6403	25.1	6.93771	23.1798	800	11.0401	39.1607	161.182	401.226
2019/8/16 5:29	232	15.6503	25.2	6.93771	23.3284	800	11.0401	39.1607	161.282	401.226
2019/8/16 5:30	320	15.6603	25.3	6.93771	21.8425	800	11.0401	39.1607	161.384	401.226
2019/8/16 5:31	402	15.6703	25.1	6.93771	21.5453	800	11.1601	39.1607	161.484	401.226
2019/8/16 5:31	483	15.6803	25.1	6.93771	22.5854	800	11.1601	39.1607	161.584	401.226
2019/8/16 5:32	567	15.6903	25.2	6.93771	24.0713	800	11.1601	39.1607	161.683	401.226

2019/8/16 5:32	651	15.7003	25.1	6.93771	23.0312	800	11.1601	39.1607	161.783	401.226
2019/8/16 5:33	738	15.7103	25.1	6.93771	22.5854	800	11.1601	39.1607	161.883	401.226
2019/8/16 5:34	827	15.7203	25.2	6.93771	22.1397	800	11.2001	39.1607	161.985	401.226
2019/8/16 5:34	915	15.7303	25.1	6.93771	20.951	800	11.2001	39.1607	162.085	401.226
2019/8/16 5:35	2	15.7403	25.2	6.93771	21.5453	800	11.2001	39.1607	162.185	401.226
2019/8/16 5:36	90	15.7503	25.3	6.93771	21.0996	800	11.2001	39.1607	162.285	401.226
2019/8/16 5:36	176	15.7603	25	6.93771	22.734	800	11.2601	39.1607	162.385	401.226
2019/8/16 5:37	256	15.7703	25.1	6.93771	22.8826	800	11.2601	39.1607	162.485	401.226
2019/8/16 5:37	343	15.7803	25.2	6.93771	21.8425	800	11.2601	39.1607	162.586	401.226
2019/8/16 5:38	425	15.7903	25.1	6.93771	21.0996	800	11.2601	39.1607	162.686	401.226
2019/8/16 5:39	507	15.8003	25.1	6.93771	21.8425	800	11.2801	39.1607	162.786	401.226
2019/8/16 5:39	588	15.8103	25.2	6.93771	21.9911	800	11.3201	39.1607	162.886	401.226
2019/8/16 5:40	673	15.8203	25.2	6.93771	21.9911	800	11.3601	39.1607	162.986	401.226
2019/8/16 5:40	759	15.8303	25.1	6.93771	23.477	800	11.3601	39.1607	163.087	401.226
2019/8/16 5:41	842	15.8403	25.2	6.93771	24.9629	800	11.3801	39.1607	163.187	401.226
2019/8/16 5:42	928	15.8503	25.2	6.93771	23.3284	800	11.4001	39.1607	163.287	401.226
2019/8/16 5:42	10	15.8603	25.1	6.93771	23.7741	800	11.5201	39.1607	163.384	401.226
2019/8/16 5:43	90	15.8703	25.2	6.93771	22.8826	800	11.5201	39.1607	163.484	401.226
2019/8/16 5:43	176	15.8803	25.2	6.93771	24.2199	800	11.5201	39.1607	163.584	401.226
2019/8/16 5:44	263	15.8903	25	6.93771	24.6657	800	11.5201	39.1607	163.684	401.226
2019/8/16 5:45	349	15.9003	25	6.93771	25.4086	800	11.5201	39.1607	163.786	401.226
2019/8/16 5:45	433	15.9103	25.2	6.93771	26.3001	800	11.5401	39.1607	163.886	401.226
2019/8/16 5:46	516	15.9203	25.1	6.93771	24.5171	800	11.5401	39.1607	163.986	401.226
2019/8/16 5:46	603	15.9303	25.1	6.93771	24.3685	800	11.5601	39.1607	164.086	401.226
2019/8/16 5:47	690	15.9403	25.2	6.93771	22.8826	800	11.6201	39.1607	164.186	401.226
2019/8/16 5:48	771	15.9503	25.2	6.93771	23.3284	800	11.6601	39.1607	164.286	401.226
2019/8/16 5:48	854	15.9603	25.1	6.93771	23.477	800	11.6601	39.1607	164.387	401.226
2019/8/16 5:49	935	15.9703	25.1	6.93771	24.8143	800	11.6601	39.1607	164.487	401.226
2019/8/16 5:49	18	15.9803	25.2	6.93771	24.0713	800	11.7001	39.1607	164.584	401.226
2019/8/16 5:50	103	15.9903	25.1	6.93771	24.0713	800	11.7801	39.1607	164.684	401.226
2019/8/16 5:51	195	16.0003	25	6.93771	23.477	800	11.8001	39.1607	164.784	401.226
2019/8/16 5:51	284	16.0103	25.2	6.94809	22.2883	800	11.9201	39.1607	164.885	401.226
2019/8/16 5:52	365	16.0203	25.1	6.92733	22.4368	800	11.9201	39.1607	164.985	401.226
2019/8/16 5:52	453	16.0303	25	6.93771	24.0713	800	11.9201	39.1607	165.085	401.226
2019/8/16 5:53	543	16.0403	25.2	6.93771	25.26	800	11.9201	39.1607	165.185	401.226

2019/8/16 5:54	634	16.0503	25.2	6.93771	24.9629	800	11.9201	39.1607	165.285	401.226
2019/8/16 5:54	712	16.0603	25.1	6.93771	23.9227	800	11.9201	39.1607	165.385	401.226
2019/8/16 5:55	798	16.0703	25.1	6.93771	23.477	800	11.9201	39.1607	165.487	401.226
2019/8/16 5:55	885	16.0803	25.3	6.93771	22.1397	800	11.9201	39.1607	165.587	401.226
2019/8/16 5:56	972	16.0903	25.1	6.93771	20.951	800	11.9401	39.1607	165.687	401.226
2019/8/16 5:57	53	16.1003	25.1	6.93771	24.0713	800	12.0001	39.1607	165.784	401.226
2019/8/16 5:57	144	16.1103	25.3	6.93771	23.3284	800	12.0001	39.1607	165.884	401.226
2019/8/16 5:58	229	16.1203	25.1	6.93771	23.7741	800	12.0201	39.1607	165.984	401.226
2019/8/16 5:58	310	16.1303	25.2	6.93771	22.1397	800	12.1201	39.1607	166.085	401.226
2019/8/16 5:59	393	16.1403	25.1	6.93771	20.951	800	12.1201	39.1607	166.185	401.226
2019/8/16 6:00	476	16.1503	25.1	6.93771	20.6538	800	12.1201	39.1607	166.285	401.226
2019/8/16 6:00	560	16.1603	25.3	6.93771	20.951	800	12.1201	39.1607	166.385	401.226
2019/8/16 6:01	642	16.1703	25.1	6.93771	20.3566	800	12.1401	39.1607	166.485	401.226
2019/8/16 6:01	730	16.1803	25.1	6.93771	22.2883	800	12.2801	39.1607	166.586	401.226
2019/8/16 6:02	814	16.1903	25.2	6.92733	21.2481	800	12.2801	39.1607	166.686	401.226
2019/8/16 6:03	903	16.2003	25.1	6.93771	19.9108	800	12.2801	39.1607	166.786	401.226
2019/8/16 6:03	985	16.2103	25.1	6.93771	19.4651	800	12.2801	39.1607	166.886	401.226
2019/8/16 6:04	76	16.2203	25.3	6.93771	18.2764	800	12.2801	39.1607	166.984	401.226
2019/8/16 6:04	158	16.2303	25.1	6.93771	17.2363	800	12.2801	39.1607	167.084	401.226
2019/8/16 6:05	245	16.2403	25.1	6.93771	19.9108	800	12.2801	39.1607	167.185	401.226
2019/8/16 6:06	326	16.2503	25.3	6.93771	19.7623	800	12.3201	39.1607	167.285	401.226
2019/8/16 6:06	410	16.2603	25.1	6.93771	20.208	800	12.3201	39.1607	167.385	401.226
2019/8/16 6:07	492	16.2703	25.2	6.93771	19.7623	800	12.3201	39.1607	167.485	401.226
2019/8/16 6:07	575	16.2803	25.2	6.93771	18.5736	800	12.3401	39.1607	167.585	401.226
2019/8/16 6:08	658	16.2903	25	6.93771	17.2363	800	12.4201	39.1607	167.685	401.226
2019/8/16 6:09	749	16.3003	25.1	6.93771	17.8306	800	12.4401	39.1607	167.786	401.226
2019/8/16 6:09	832	16.3103	25.2	6.93771	19.7623	800	12.5001	39.1607	167.886	401.226
2019/8/16 6:10	916	16.3203	25.1	6.93771	18.8707	800	12.5001	39.1607	167.986	401.226
2019/8/16 6:10	0	16.3303	25.1	6.93771	20.5052	800	12.5601	39.1607	168.086	401.226
2019/8/16 6:11	84	16.3403	25.2	6.93771	18.7221	800	12.5601	39.1607	168.186	401.226
2019/8/16 6:12	169	16.3503	25.2	6.93771	16.9391	800	12.5601	39.1607	168.286	401.226
2019/8/16 6:12	255	16.3603	25.1	6.94809	16.6419	800	12.6601	39.1607	168.387	401.226
2019/8/16 6:13	338	16.3703	25.2	6.93771	17.8306	800	12.6601	39.1607	168.487	401.226
2019/8/16 6:13	418	16.3803	25.1	6.93771	17.682	800	12.6601	39.1607	168.587	401.226
2019/8/16 6:14	503	16.3903	25.1	6.93771	18.8707	800	12.6601	39.1607	168.687	401.226

2019/8/16 6:15	592	16.4003	25.3	6.93771	17.3848	800	12.7001	39.1607	168.787	401.226
2019/8/16 6:15	687	16.4103	25.1	6.93771	16.1961	800	12.7001	39.1607	168.887	401.226
2019/8/16 6:16	770	16.4203	25.1	6.93771	16.1961	800	12.8201	39.1607	168.989	401.226
2019/8/16 6:16	858	16.4303	25.3	6.93771	14.8588	800	12.8201	39.1607	169.089	401.226
2019/8/16 6:17	943	16.4403	25.1	6.93771	15.899	800	12.8201	39.1607	169.189	401.226
2019/8/16 6:18	28	16.4503	25.2	6.93771	16.4933	800	12.8401	39.1607	169.286	401.226
2019/8/16 6:18	115	16.4603	25.3	6.93771	17.8306	800	12.8401	39.1607	169.386	401.226
2019/8/16 6:19	207	16.4703	25.1	6.93771	16.9391	800	12.8801	39.1607	169.486	401.226
2019/8/16 6:19	287	16.4803	25.1	6.93771	16.1961	800	12.8801	39.1607	169.587	401.226
2019/8/16 6:20	373	16.4903	25.2	6.93771	14.8588	800	13.0201	39.1607	169.687	401.226
2019/8/16 6:21	455	16.5003	25.1	6.93771	15.3046	800	13.0201	39.1607	169.787	401.525
2019/8/16 6:21	337	16.5103	25.1	6.93771	15.899	800	13.0201	39.1607	169.883	402.125
2019/8/16 6:22	600	16.5203	25.3	6.93771	17.3848	800	13.0201	39.1607	169.963	402.575
2019/8/16 6:22	724	16.5303	25.1	6.93771	17.2363	800	13.0201	39.1607	170.044	403.175
2019/8/16 6:23	806	16.5403	25.2	6.93771	17.5334	800	13.1401	39.1607	170.124	403.675
2019/8/16 6:24	889	16.5503	25.3	6.93771	16.4933	800	13.1401	39.1607	170.205	404.275
2019/8/16 6:24	971	16.5603	25.1	6.93771	17.5334	800	13.1401	39.1607	170.285	404.875
2019/8/16 6:25	56	16.5703	25.2	6.93771	17.8306	800	13.1401	39.1607	170.363	405.325
2019/8/16 6:25	136	16.5803	25.2	6.93771	17.8306	800	13.1801	39.1607	170.443	405.924
2019/8/16 6:26	119	16.5903	25.1	6.93771	17.5334	800	13.1801	39.1607	170.524	406.374
2019/8/16 6:27	204	16.6003	25.2	6.93771	17.682	800	13.1801	39.1607	170.604	406.524
2019/8/16 6:27	395	16.6103	25.1	6.93771	17.2363	800	13.3001	39.1607	170.685	406.524
2019/8/16 6:28	481	16.6203	25.3	6.93771	17.3848	800	13.3001	39.1607	170.765	406.524
2019/8/16 6:28	578	16.6303	25.1	6.93771	13.9673	800	13.3001	39.1607	170.845	406.524
2019/8/16 6:29	665	16.6403	25	6.93771	15.6018	800	13.3001	39.1607	170.926	406.524
2019/8/16 6:30	753	16.6503	25.2	6.93771	13.2244	800	13.3201	39.1607	171.007	406.524
2019/8/16 6:30	836	16.6603	25.3	6.93771	12.3328	800	13.3201	39.1607	171.087	406.524
2019/8/16 6:31	922	16.6703	25.1	6.93771	13.9673	800	13.4201	39.1607	171.167	406.524
2019/8/16 6:31	0	16.6803	25.2	6.93771	15.156	800	13.4201	39.1607	171.247	406.524
2019/8/16 6:32	80	16.6903	25.3	6.93771	14.7103	800	13.4201	39.1607	171.325	406.524
2019/8/16 6:33	163	16.7003	25.1	6.93771	15.899	800	13.5001	39.1607	171.406	406.524
2019/8/16 6:33	246	16.7103	25.2	6.93771	16.0475	800	13.5401	39.1607	171.487	406.524
2019/8/16 6:34	331	16.7203	25.2	6.93771	16.1961	800	13.5401	39.1607	171.567	406.524
2019/8/16 6:34	414	16.7303	25.1	6.93771	16.0475	800	13.6801	39.1607	171.647	406.524
2019/8/16 6:35	497	16.7403	25.1	6.93771	16.4933	800	13.6801	39.1607	171.727	406.524

2019/8/16 6:36	583	16.7503	25.2	6.93771	13.9673	800	13.7401	39.1607	171.807	406.524
2019/8/16 6:36	670	16.7603	25.1	6.93771	15.899	800	13.7401	39.1607	171.889	406.524
2019/8/16 6:37	752	16.7703	25.2	6.93771	15.3046	800	13.7801	39.1607	171.969	406.524
2019/8/16 6:37	839	16.7803	25.1	6.93771	15.899	800	13.8401	39.1607	172.049	406.524
2019/8/16 6:38	927	16.7903	25.1	6.93771	17.2363	800	13.8401	39.1607	172.129	406.524
2019/8/16 6:39	4	16.8003	25.2	6.93771	18.1278	800	13.9001	39.1607	172.207	406.524
2019/8/16 6:39	95	16.8103	25.3	6.93771	18.1278	800	13.9001	39.1607	172.288	406.524
2019/8/16 6:40	178	16.8203	25.1	6.93771	18.8707	800	13.9001	39.1607	172.369	406.524
2019/8/16 6:40	263	16.8303	25.2	6.93771	19.1679	800	13.9601	39.1607	172.449	406.524
2019/8/16 6:41	346	16.8403	25.3	6.94809	19.0193	800	13.9601	39.1607	172.529	406.524
2019/8/16 6:42	427	16.8503	25.1	6.93771	17.9792	800	14.0801	39.1607	172.609	406.524
2019/8/16 6:42	510	16.8603	25.2	6.93771	18.7221	800	14.1001	39.1607	172.69	406.524
2019/8/16 6:43	594	16.8703	25.2	6.93771	15.899	800	14.1401	39.1607	172.771	406.524
2019/8/16 6:43	687	16.8803	25	6.93771	17.2363	800	14.2401	39.1607	172.851	406.524
2019/8/16 6:44	767	16.8903	25.1	6.93771	16.7905	800	14.2401	39.1607	172.931	406.524
2019/8/16 6:45	857	16.9003	25.2	6.93771	17.3848	800	14.2401	39.1607	173.012	406.524
2019/8/16 6:45	941	16.9103	25.1	6.93771	17.3848	800	14.2401	39.1607	173.092	406.524
2019/8/16 6:46	23	16.9203	25	6.93771	19.6137	800	14.2401	39.1607	173.17	406.524
2019/8/16 6:46	106	16.9303	25.1	6.94809	19.9108	800	14.4001	39.1607	173.25	406.524
2019/8/16 6:47	187	16.9403	25.2	6.93771	20.8024	800	14.4001	39.1607	173.331	406.524
2019/8/16 6:48	271	16.9503	25.1	6.93771	20.3566	800	14.4001	39.1607	173.411	406.524
2019/8/16 6:48	356	16.9603	25.1	6.93771	20.5052	800	14.4401	39.1607	173.492	406.524
2019/8/16 6:49	439	16.9703	25.3	6.93771	20.8024	800	14.4801	39.1607	173.572	406.524
2019/8/16 6:49	520	16.9803	25.1	6.93771	17.9792	800	14.6201	39.1607	173.652	406.524
2019/8/16 6:50	601	16.9903	25.1	6.93771	17.8306	800	14.6201	39.1607	173.732	406.524
2019/8/16 6:51	685	17.0003	25.3	6.93771	18.2764	800	14.6201	39.1607	173.814	406.524
2019/8/16 6:51	772	17.0103	25.1	6.93771	17.0877	800	14.6401	39.1607	173.894	406.524
2019/8/16 6:52	851	17.0203	25.1	6.93771	18.8707	800	14.7601	39.1607	173.974	406.524
2019/8/16 6:52	635	17.0303	25.2	6.93771	18.5736	800	14.7601	39.1607	174.054	406.524
2019/8/16 6:53	65	17.0403	25.1	6.93771	18.2764	800	14.7601	39.1607	174.132	406.524
2019/8/16 6:54	143	17.0503	25	6.93771	19.9108	800	14.8001	39.1607	174.212	406.524
2019/8/16 6:54	0	17.0603	25.2	6.93771	19.6137	800	14.9002	39.1607	174.294	406.524
2019/8/16 6:55	268	17.0703	25.1	6.93771	18.7221	800	14.9002	39.1607	174.374	406.524
2019/8/16 6:55	353	17.0803	25.1	6.93771	19.9108	800	14.9002	39.1607	174.454	406.524
2019/8/16 6:56	437	17.0903	25.2	6.94809	17.682	800	15.0602	39.1607	174.534	406.524

2019/8/16 6:57	518	17.1003	25.2	6.93771	18.7221	800	15.0602	39.1607	174.614	406.524
2019/8/16 6:57	603	17.1103	25.1	6.93771	17.2363	800	15.0602	39.1607	174.696	406.524
2019/8/16 6:58	684	17.1203	25.2	6.93771	16.0475	800	15.0602	39.1607	174.776	406.524
2019/8/16 6:58	772	17.1303	25.2	6.93771	16.0475	800	15.1002	39.1607	174.856	406.524
2019/8/16 6:59	854	17.1403	25	6.93771	18.2764	800	15.1202	39.1607	174.936	406.524
2019/8/16 7:00	935	17.1503	25.2	6.93771	18.425	800	15.1602	39.1607	175.017	406.524
2019/8/16 7:00	15	17.1603	25.2	6.93771	18.5736	800	15.2802	39.1607	175.095	406.524
2019/8/16 7:01	100	17.1703	25	6.93771	19.9108	800	15.2802	39.1607	175.176	406.524
2019/8/16 7:01	185	17.1803	25	6.93771	21.0996	800	15.2802	39.1607	175.256	406.524
2019/8/16 7:02	268	17.1903	25.2	6.93771	20.5052	800	15.2802	39.1607	175.336	406.524
2019/8/16 7:03	354	17.2003	25.2	6.93771	18.1278	800	15.4002	39.1607	175.416	406.524
2019/8/16 7:03	441	17.2103	25.1	6.93771	20.0594	800	15.4002	39.1607	175.497	406.524
2019/8/16 7:04	529	17.2203	25.2	6.93771	18.2764	800	15.4002	39.1607	175.577	406.524
2019/8/16 7:04	614	17.2303	25.1	6.93771	18.7221	800	15.4002	39.1607	175.658	406.524
2019/8/16 7:05	699	17.2403	25.1	6.93771	18.2764	800	15.5002	39.1607	175.738	406.524
2019/8/16 7:06	778	17.2503	25.2	6.93771	17.3848	800	15.5202	39.1607	175.819	406.524
2019/8/16 7:06	863	17.2603	25.2	6.93771	17.0877	800	15.5802	39.1607	175.899	406.524
2019/8/16 7:07	943	17.2703	25	6.93771	18.7221	800	15.5802	39.1607	175.979	406.524
2019/8/16 7:07	23	17.2803	25	6.93771	19.9108	800	15.5802	39.1607	176.057	406.524
2019/8/16 7:08	114	17.2903	25.2	6.93771	19.6137	800	15.7002	39.1607	176.138	406.524
2019/8/16 7:09	201	17.3003	25.1	6.93771	19.4651	800	15.7002	39.1607	176.218	406.524
2019/8/16 7:09	294	17.3103	25.1	6.92733	20.208	800	15.7002	39.1607	176.299	406.524
2019/8/16 7:10	375	17.3203	25.2	6.93771	20.6538	800	15.7002	39.1607	176.379	406.524
2019/8/16 7:10	463	17.3303	25.2	6.93771	18.425	800	15.7002	39.1607	176.459	406.524
2019/8/16 7:11	548	17.3403	25	6.93771	17.9792	800	15.7802	39.1607	176.54	406.524
2019/8/16 7:12	632	17.3503	25.1	6.93771	19.1679	800	15.7802	39.1607	176.621	406.524
2019/8/16 7:12	716	17.3603	25.3	6.93771	17.5334	800	15.8602	39.1607	176.701	406.524
2019/8/16 7:13	798	17.3703	25.1	6.93771	18.1278	800	15.8602	39.1607	176.781	406.524
2019/8/16 7:13	885	17.3803	25.1	6.93771	19.9108	800	15.8602	39.1607	176.861	406.524
2019/8/16 7:14	967	17.3903	25.2	6.93771	20.3566	800	15.9602	39.1607	176.941	406.524
2019/8/16 7:15	49	17.4003	25.2	6.93771	20.951	800	15.9602	39.1607	177.02	406.524
2019/8/16 7:15	142	17.4103	25.1	6.93771	22.4368	800	16.0002	39.1607	177.101	406.524
2019/8/16 7:16	220	17.4203	25.1	6.93771	22.4368	800	16.0002	39.1607	177.181	406.524
2019/8/16 7:16	302	17.4303	25.2	6.93771	21.6939	800	16.0002	39.1607	177.261	406.524
2019/8/16 7:17	387	17.4403	25.1	6.93771	21.6939	800	16.1402	39.1607	177.341	406.524

2019/8/16 7:18	468	17.4503	25.2	6.93771	20.8024	800	16.1402	39.1607	177.421	406.524
2019/8/16 7:18	549	17.4603	25.2	6.93771	19.3165	800	16.1402	39.1607	177.502	406.524
2019/8/16 7:19	635	17.4703	25.1	6.93771	19.3165	800	16.1802	39.1607	177.583	406.524
2019/8/16 7:19	725	17.4803	25.2	6.93771	20.208	800	16.1802	39.1607	177.663	406.524
2019/8/16 7:20	812	17.4903	25.1	6.93771	19.0193	800	16.1802	39.1607	177.743	406.524
2019/8/16 7:21	897	17.5003	25.1	6.93771	20.6538	800	16.2602	39.1607	177.824	406.524
2019/8/16 7:21	980	17.5103	25.2	6.93771	21.3967	800	16.2602	39.1607	177.904	406.524
2019/8/16 7:22	65	17.5203	25.2	6.93771	22.2883	800	16.2602	39.1607	177.983	406.524
2019/8/16 7:22	154	17.5303	25.1	6.93771	24.0713	800	16.4002	39.1607	178.063	406.524
2019/8/16 7:23	235	17.5403	25.2	6.93771	24.6657	800	16.4002	39.1607	178.143	406.524
2019/8/16 7:24	319	17.5503	25.2	6.93771	23.9227	800	16.4002	39.1607	178.223	406.524
2019/8/16 7:24	406	17.5603	25.1	6.93771	24.8143	800	16.4002	39.1607	178.304	406.524
2019/8/16 7:25	494	17.5703	25.2	6.93771	23.9227	800	16.4002	39.1607	178.384	406.524
2019/8/16 7:25	580	17.5803	25.2	6.93771	22.1397	800	16.5602	39.1607	178.465	406.524
2019/8/16 7:26	668	17.5903	25.1	6.93771	23.0312	800	16.5602	39.1607	178.545	406.524
2019/8/16 7:27	753	17.6003	25.1	6.93771	23.6256	800	16.5602	39.1607	178.626	406.524
2019/8/16 7:27	830	17.6103	25.3	6.93771	23.9227	800	16.5602	39.1607	178.706	406.524
2019/8/16 7:28	907	17.6203	25.1	6.93771	25.8544	800	16.5802	39.1607	178.786	406.524
2019/8/16 7:28	991	17.6303	25.2	6.93771	27.1917	800	16.7002	39.1607	178.866	406.524
2019/8/16 7:29	75	17.6403	25.2	6.93771	28.6776	800	16.7002	39.1607	178.945	406.524
2019/8/16 7:30	157	17.6503	25.1	6.93771	29.2719	800	16.7002	39.1607	179.025	406.524
2019/8/16 7:30	243	17.6603	25.1	6.93771	30.1634	800	16.7002	39.1607	179.106	406.524
2019/8/16 7:31	328	17.6703	25.3	6.93771	29.5691	800	16.8202	39.1607	179.186	406.524
2019/8/16 7:31	412	17.6803	25.1	6.93771	29.4205	800	16.8202	39.1607	179.266	406.524
2019/8/16 7:32	500	17.6903	25.1	6.93771	27.9346	800	16.8202	39.1607	179.347	406.524
2019/8/16 7:33	586	17.7003	25.2	6.93771	27.6374	800	16.8202	39.1607	179.428	406.524
2019/8/16 7:33	673	17.7103	25.2	6.93771	28.3804	800	16.8202	39.1607	179.508	406.524
2019/8/16 7:34	756	17.7203	25.1	6.93771	30.312	800	16.8402	39.1607	179.588	406.524
2019/8/16 7:34	844	17.7303	25.1	6.93771	31.9465	800	16.8602	39.1607	179.668	406.524
2019/8/16 7:35	429	17.7403	25.2	6.94809	32.5409	800	16.9002	39.1607	179.747	406.524
2019/8/16 7:36	510	17.7503	25.1	6.93771	33.8782	800	17.0002	39.1607	179.829	406.524
2019/8/16 7:36	590	17.7603	25	6.93771	36.9985	800	17.0002	39.1607	179.909	406.524
2019/8/16 7:37	674	17.7703	25.1	6.93771	36.8499	800	17.1002	39.1607	179.989	406.524
2019/8/16 7:37	756	17.7803	25.3	6.93771	37.5929	800	17.1002	39.1607	180.069	406.524
2019/8/16 7:38	843	17.7903	25.1	6.93771	37.1471	800	17.1002	39.1607	180.149	406.524

2019/8/16 7:39	929	17.8003	25.1	6.93771	38.7816	800	17.1002	39.1607	180.23	406.524
2019/8/16 7:39	7	17.8103	25.2	6.93771	39.3759	800	17.1602	39.1607	180.309	406.524
2019/8/16 7:40	91	17.8203	25.2	6.93771	38.3358	800	17.2002	39.1607	180.389	406.524
2019/8/16 7:40	175	17.8303	25.1	6.93771	40.7132	800	17.2202	39.1607	180.469	406.524
2019/8/16 7:41	257	17.8403	25.2	6.93771	42.6449	800	17.2602	39.1607	180.549	406.524
2019/8/16 7:42	345	17.8503	25.2	6.93771	43.3878	800	17.3002	39.1607	180.629	406.524
2019/8/16 7:42	430	17.8603	25	6.93771	46.0624	800	17.3002	39.1607	180.71	406.524
2019/8/16 7:43	509	17.8703	25.1	6.93771	48.1426	800	17.3802	39.1607	180.791	406.524
2019/8/16 7:43	593	17.8803	25.1	6.93771	49.7771	800	17.4002	39.1607	180.871	406.524
2019/8/16 7:44	681	17.8903	25.3	6.93771	49.3313	800	17.4802	39.1607	180.951	406.524
2019/8/16 7:45	762	17.9003	25.1	6.93771	50.2229	800	17.5002	39.1607	181.032	406.524
2019/8/16 7:45	843	17.9103	25	6.93771	50.3715	800	17.5202	39.1607	181.112	406.524
2019/8/16 7:46	932	17.9203	25.2	6.93771	49.7771	800	17.5202	39.1607	181.192	406.524
2019/8/16 7:46	20	17.9303	25.2	6.93771	48.5884	800	17.5202	39.1607	181.271	406.524
2019/8/16 7:47	102	17.9403	25.1	6.93771	48.5884	800	17.6002	39.1607	181.351	406.524
2019/8/16 7:48	184	17.9503	25.1	6.93771	51.1144	800	17.6002	39.1607	181.431	406.524
2019/8/16 7:48	264	17.9603	25.1	6.93771	52.4517	800	17.6002	39.1607	181.512	406.524
2019/8/16 7:49	344	17.9703	25.2	6.93771	53.3432	800	17.6802	39.1607	181.592	406.524
2019/8/16 7:49	425	17.9803	25.1	6.93771	54.0862	800	17.6802	39.1607	181.672	406.524
2019/8/16 7:50	512	17.9903	25.1	6.94809	55.7206	800	17.6802	39.1607	181.753	406.524
2019/8/16 7:51	603	18.0003	25.2	6.93771	55.8692	800	17.6802	39.1607	181.834	406.524
2019/8/16 7:51	692	18.0103	25.2	6.93771	55.5721	800	17.6802	39.1607	181.914	406.524
2019/8/16 7:52	777	18.0203	25.1	6.93771	57.2065	800	17.6802	39.1607	181.994	406.524
2019/8/16 7:52	857	18.0303	25.1	6.93771	55.2749	800	17.6802	39.1607	182.074	406.524
2019/8/16 7:53	939	18.0403	25.2	6.93771	54.8291	800	17.7802	39.1607	182.156	406.524
2019/8/16 7:54	25	18.0503	25.2	6.93771	53.1946	800	17.8002	39.1607	182.233	406.524
2019/8/16 7:54	104	18.0603	25.1	6.93771	54.2348	800	17.8002	39.1607	182.314	406.524
2019/8/16 7:55	192	18.0703	25.1	6.93771	55.4235	800	17.8002	39.1607	182.394	406.524
2019/8/16 7:55	276	18.0803	25.2	6.93771	55.4235	800	17.8002	39.1607	182.474	406.524
2019/8/16 7:56	363	18.0903	25.1	6.93771	55.8692	800	17.8002	39.1607	182.554	406.524
2019/8/16 7:57	444	18.1003	25	6.93771	57.9495	800	17.8202	39.1607	182.634	406.524
2019/8/16 7:57	526	18.1103	25.1	6.93771	57.8009	800	17.8402	39.1607	182.716	406.524
2019/8/16 7:58	611	18.1203	25.2	6.93771	57.5037	800	17.8402	39.1607	182.796	406.524
2019/8/16 7:58	698	18.1303	25.1	6.93771	56.4636	800	17.9002	39.1607	182.876	406.524
2019/8/16 7:59	780	18.1403	25.1	6.93771	57.6523	800	17.9002	39.1607	182.956	406.524

2019/8/16 8:00	861	18.1503	25.1	6.93771	55.8692	800	17.9002	39.1607	183.037	406.524
2019/8/16 8:00	946	18.1603	25.2	6.93771	55.2749	800	17.9202	39.1607	183.118	406.524
2019/8/16 8:01	532	18.1703	25.2	6.93771	55.1263	800	17.9202	39.1607	183.197	406.524
2019/8/16 8:01	617	18.1803	25	6.93771	56.1664	800	17.9202	39.1607	183.277	406.524
2019/8/16 8:02	699	18.1903	25.1	6.93771	56.4636	800	17.9202	39.1607	183.357	406.524
2019/8/16 8:03	783	18.2003	25.2	6.93771	57.0579	800	17.9202	39.1607	183.438	406.524
2019/8/16 8:03	870	18.2103	25.2	6.93771	57.5037	100	17.9202	39.1607	183.518	406.524
2019/8/16 8:04	743	18.2203	25.1	6.93771	26.7459	0	17.9202	39.1607	183.585	406.524
